# Author Correction: Platinum-induced upregulation of ITGA6 promotes chemoresistance and spreading in ovarian cancer

**DOI:** 10.1038/s44321-024-00099-x

**Published:** 2024-07-12

**Authors:** Alice Gambelli, Anna Nespolo, Gian Luca Rampioni Vinciguerra, Eliana Pivetta, Ilenia Pellarin, Milena S Nicoloso, Chiara Scapin, Linda Stefenatti, Ilenia Segatto, Andrea Favero, Sara D’Andrea, Maria Teresa Mucignat, Michele Bartoletti, Emilio Lucia, Monica Schiappacassi, Paola Spessotto, Vincenzo Canzonieri, Giorgio Giorda, Fabio Puglisi, Andrea Vecchione, Barbara Belletti, Maura Sonego, Gustavo Baldassarre

**Affiliations:** 1 Molecular Oncology Unit, Centro di Riferimento Oncologico di Aviano (CRO) IRCCS, National Cancer Institute, Aviano, PN Italy; 2 Deparment of Medical Oncology, Centro di Riferimento Oncologico di Aviano (CRO) IRCCS, National Cancer Institute, Aviano, PN Italy; 3 Gynecological Surgery Unit, Centro di Riferimento Oncologico di Aviano (CRO) IRCCS, National Cancer Institute, Aviano, PN Italy; 4 Pathology Unit, Centro di Riferimento Oncologico di Aviano (CRO) IRCCS, National Cancer Institute, Aviano, PN Italy; 5 Department of Medical, Surgical and Health Sciences, University of Trieste, Trieste, TS Italy; 6 Department of Medicine, University of Udine, Udine, UD Italy; 7 Department of Clinical and Molecular Medicine, Faculty of Medicine and Psychology, Sant’Andrea Hospital, University of Rome “Sapienza”, Rome, Italy

## Abstract

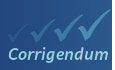

**Correction to:** *EMBO Molecular Medicine* (2024) 16:1162–1192. 10.1038/s44321-024-00069-3 | Published online 24 April 2024

In this article, the author’s name ‘Linda Stefenatti’ was incorrectly written as ‘Linda Stefanatti’. The original article has been corrected.

