# Supplementary material for: Platinum-induced upregulation of ITGA6 promotes chemoresistance and spreading in ovarian cancer
Source: EMBO Mol Med. 2024 Apr 24;16(5):7. doi: 10.1038/s44321-024-00069-3 (PMC11099142; doi:10.1038/s44321-024-00069-3)
Supplement: Supplementary file 1 — Appendix [file 44321_2024_69_MOESM1_ESM.pdf]

**Appendix Information**

# Platinum-induced upregulation of ITGA6 promotes chemoresistance and spreading in ovarian cancer

Alice Gambelli, Anna Nespolo, Gian Luca Rampioni Vinciguerra, Eliana Pivetta, Ilenia Pellarin, Milena S. Nicoloso, Chiara Scapin, Linda Stefanatti, Ilenia Segatto, Andrea Favero, Sara D'Andrea, Maria Teresa Mucignat, Michele Bartoletti, Emilio Lucia, Monica Schiappacassi, Paola Spessotto, Vincenzo Canzonieri, Giorgio Giorda, Fabio Puglisi, Andrea Vecchione, Barbara Belletti, Maura Sonego and Gustavo Baldassarre
[1]

## Table of Contents

| | | |
|---|---|---|
| **Appendix Figure S1** | related to Figure 3 | Page 2 |
| **Appendix Figure S2** | related to Figure 4 | Page 4 |
| **Appendix Figure S3** | related to Figure 4 | Page 5 |
| **Appendix Figure S4** | related to Figure 5 | Page 6 |
| **Appendix Figure S5** | related to Figure 6 | Page 8 |
| **Appendix Figure S6** | related to Figure 7 | Page 10 |
| **Appendix Table S1** | Clinic-pathological features of ovarian cancer patients | Page 12 |
| **Appendix Table S2** | List of phospho-RTKs evaluated by antibody array | Page 13 |
| **Appendix Table S3** | List of cytokines evaluated by antibody array | Page 14 |
| **Appendix Table S4** | List of antibodies utilized in the study | Page 16 |
| **Appendix Table S5** | List of Oligonucleotides used | Page 18 |

[1]

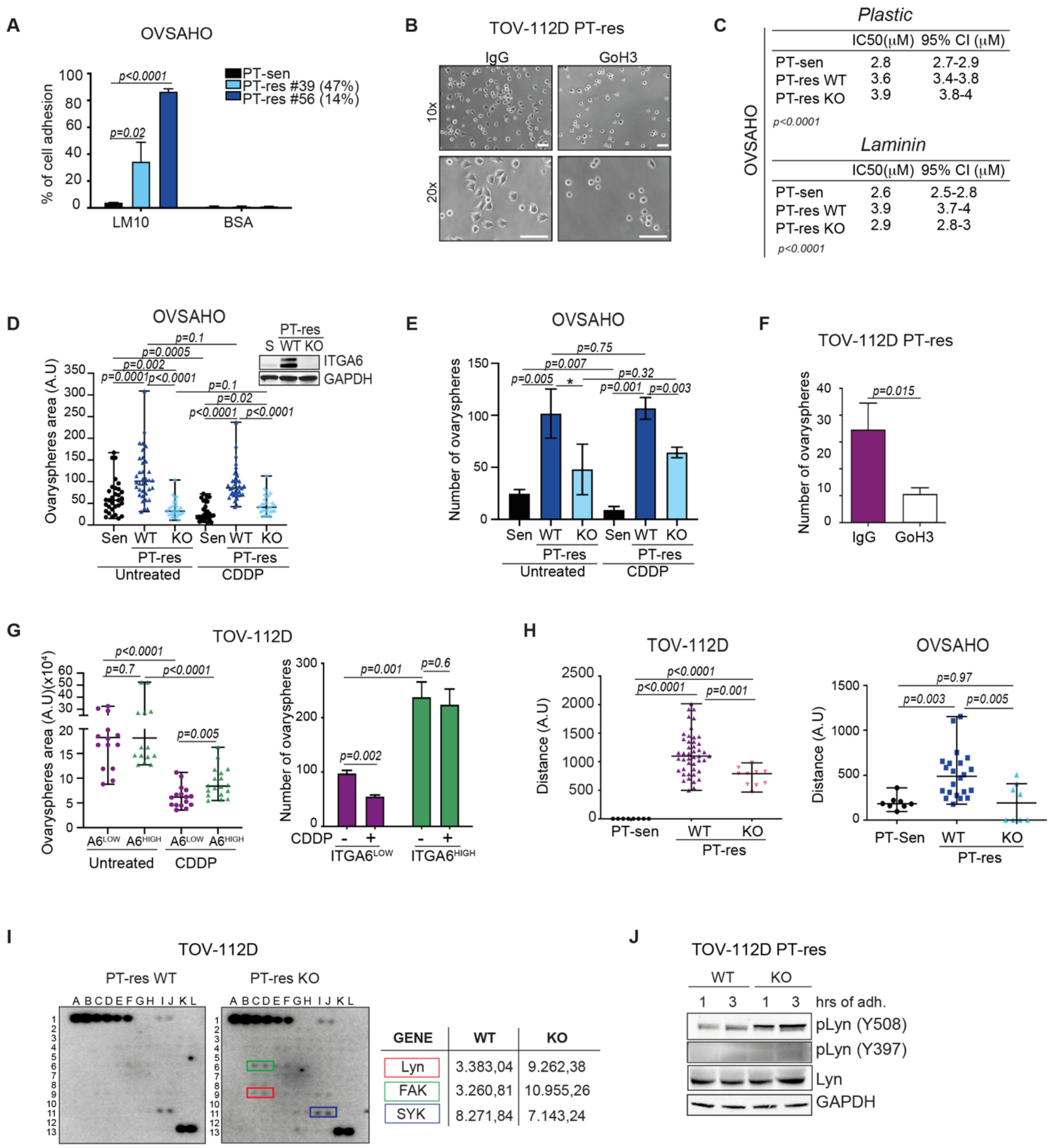


| | IC50(μM) | 95% CI (μM) |
|---|---|---|
| **Plastic** | | |
| PT-sen | 2.8 | 2.7-2.9 |
| PT-res WT | 3.6 | 3.4-3.8 |
| PT-res KO | 3.9 | 3.8-4 |
| p<0.0001 | | |
| **Laminin** | | |
| PT-sen | 2.6 | 2.5-2.8 |
| PT-res WT | 3.9 | 3.7-4 |
| PT-res KO | 2.9 | 2.8-3 |
| p<0.0001 | | |

| GENE | WT | KO |
|---|---|---|
| Lyn | 3.383,04 | 9.262,38 |
| FAK | 3.260,81 | 10.955,26 |
| SYK | 8.271,84 | 7.143,24 |

**Appendix Figure S1 (related to Figure 3). ITGA6 mediates higher metastatic capability of EOC PT-res cells.**

A) Expression of Graph reporting the percentage of cell adhesion on laminin 10 (LM10) coating of OVSAHO PT-sen and PT-res clones measured by CAFCA assay. In the legend is reported the percentage of ITGA6 positive cells for each PT-res clone. B) Representative phase-contrast images, taken with 10X or 20X objectives as indicated, of TOV-112D PT-res cells plated on LM10 coated dishes in the presence of IgG (as control) or of the specific anti-ITGA6 blocking antibody (GoH3 Ab). Scale bars, 50 μm. C) Tables reporting the IC50 and the confidence interval (CI) of OVSAHO

PT-sen, PT-res WT and PT-res ITGA6KO cells plated on plastic or in Laminin and treated with increasing doses of CDDP for 72 hours. Fisher's exact test was used to calculate the global p value reported under the tables. D-E) Graph reporting the area (D) and the number (C) of ovaryspheres formed by OVSAHO PT-sen, PT-res ITGA6WT and ITGA6KO cells treated or not with CDDP (2μM) for 24 hours. Data reported are the mean (±SD) of two independent experiments performed in triplicate, in which at least 10 randomly selected fields were analyzed. The inset in D reports the ITGA6 expression in the used cells analyzed by western blot (S=PT-sen cells). F) Graph reporting the number of ovaryspheres formed by TOV-112D PT-res in presence of IgG (as control) or of GoH3 Ab. G) Graph reporting the area (left) and the number (right) of ovaryspheres formed by TOV-112D ITGA6$^{HIGH}$ and ITGA6$^{LOW}$ subpopulations treated or not with CDDP. Data represent the mean (±SD) of two independent experiments performed in triplicate, in which at least 10 randomly selected fields were analyzed. H) Graph reporting the distance covered by the individual cells from the edge of the drop (mean ± SD of three independent experiments, in which at least 10 randomly selected fields were analyzed) in matrigel evasion assay of TOV-112D (left) and OVSAHO (right) parental, PT-res WT and KO cells. I) Western blots analysis of RTK array using whole cell lysates extracted from TOV112D PT-res ITGA6WT and KO allowed to adhere 3 hrs on LM10 coated dishes. Colored boxed spots highlight differentially expressed RTKs. On the right, the table reports the expression of the three differentially phosphorylated proteins in WT and KO cells, expressed as normalized arbitrary units. J) Western blot analysis evaluating the expression of the indicated proteins in lysates of TOV-112D PT-res ITGA6WT and ITGA6KO cells plated on LM10 coated dishes for 1 and 3 hours. In D and J, GAPDH was used as loading control. In A and D-H statistical significance was determined by a two-tailed, unpaired Student's t-test (Exact *p* values were reported on graphs).

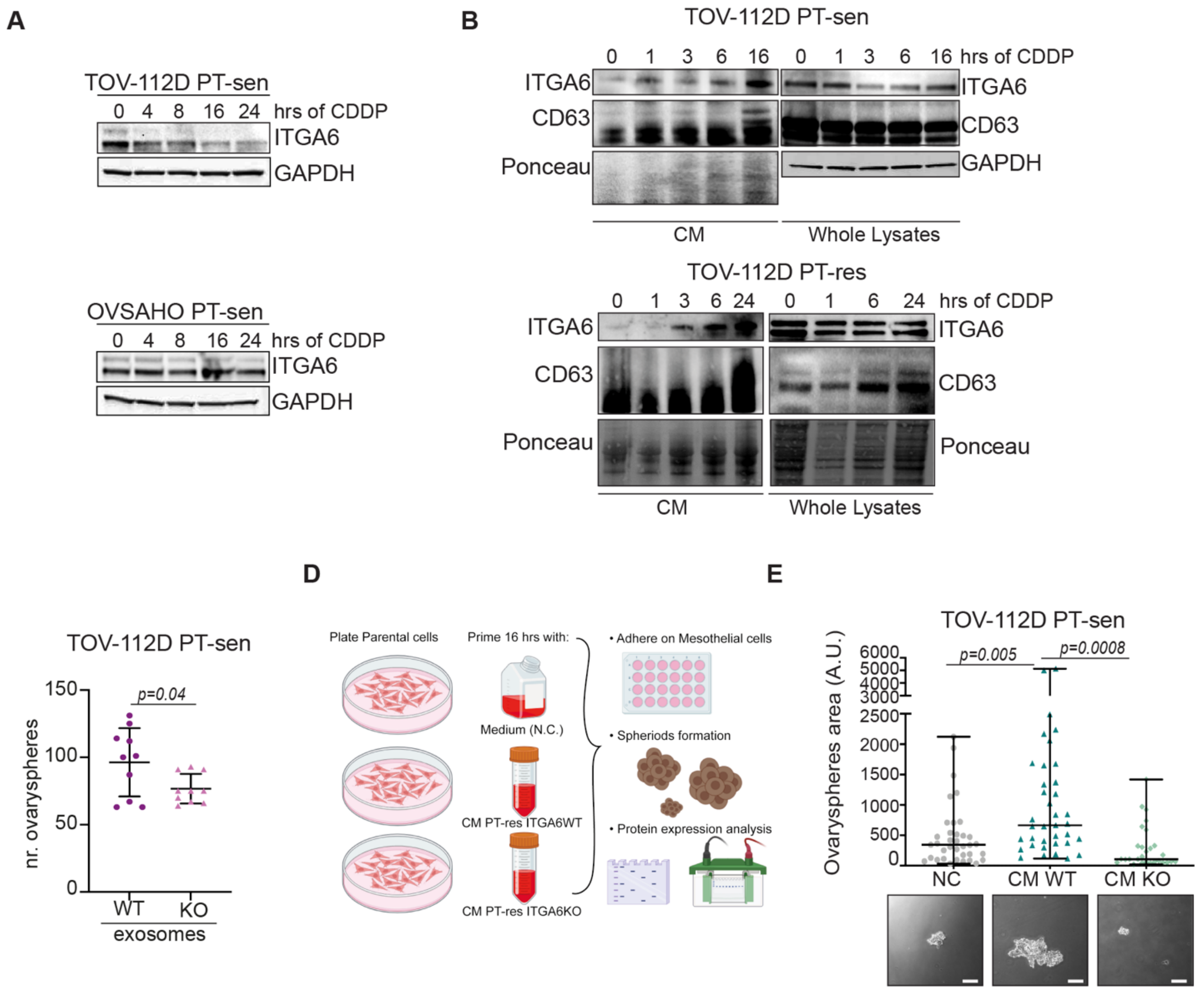


**Appendix Figure S2 (related to Figure 4). Secreted ITGA6 primes PT-sen cells to adhere and grow on mesothelial cells**

A) Western blot analysis of ITGA6 expression in whole lysates of TOV-112D and OVSAHO PT-sen cells treated with CDDP for the indicated time points. B) Western blot analysis of ITGA6 and CD63 expression in conditioned medium (CM) and whole lysates of TOV-112D PT-sen (top) and PT-res (bottom) cells treated with CDDP (25µM) for the indicated time points. In A and B, GAPDH and Ponceau were used as loading control. C) Graphs reporting the number of ovaryspheres formed by TOV-112D parental cells primed with exosomes of TOV-112D PT-res ITGA6WT and KO cells and then plated on a mesothelial cells monolayer. D) Schematic representation of the experimental procedures utilized to test the biological activity of ITGA6 secreted in contitioned medium (CM). TOV-112D PT-sen cells were incubated for 16 hours with the CM from WT or ITGA6KO TOV-112D PT-res cells or control medium and then challenged in adhesion assays on mesothelial cells, ovaryspheres formation assays or processed for western blot analyses. E) Graph (top) and representative phase-contrast images (bottom) reporting the area of ovaryspheres formed by TOV-112D parental cells treated as specified in D then plated on polyhema-coated dishes. Scale bar =

50µM In C and E, statistical significance was determined by a two-tailed, unpaired Student's t-test (Exact *p* values were reported on graphs).

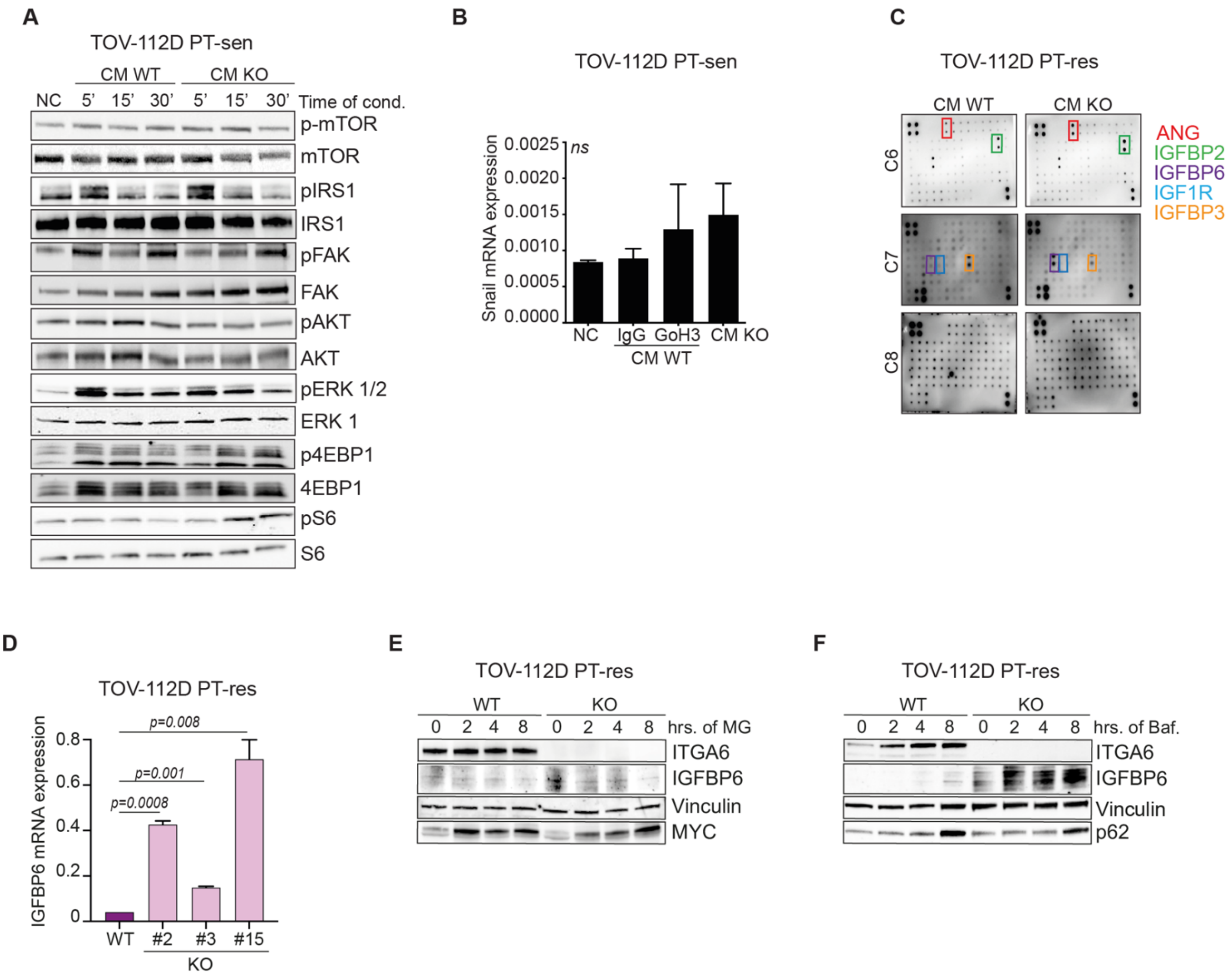


**Appendix Figure S3 (related to Figure 4).**

A) Western blot analysis of the indicated proteins in whole lysates of TOV-112D PT-sen cells challenged or not for the indicated time points with CM of TOV-112D PT-res WT or ITGA6KO cells. B) Graph reporting the mRNA expression of Snail in TOV-112D PT-sen cells challenged with CM of PT-res ITGA6 WT in presence or not of the specific anti-ITGA6 GoH3, or with CM of PT-res ITGA6 KO cells. C) Western blots of cytokine arrays (C6-C8 arrays) using CM of TOV112D PT-res WT and ITGA6KO cells. The most significantly differentially expressed proteins are highlighted in colored boxes. D) qRT-PCR evaluating the mRNA expression of IGFBP6 in TOV-112D PT-res WT and ITGA6KO clones. Statistical significance was determined by a two-tailed, unpaired Student's t-test (Exact *p* values were reported on graphs). E-F) Western Blot analysis of IGFBP6

expression in TOV-112D PT-res exposed to a time-course treatment with the proteasome inhibitor, MG132 (E) or with inhibitor of autophagosome-lysosome fusion, Bafylomicin (F). MYC and p62 were used as positive control of MG132 and Bafylomicin activity, respectively. Vinculin was used as loading control.

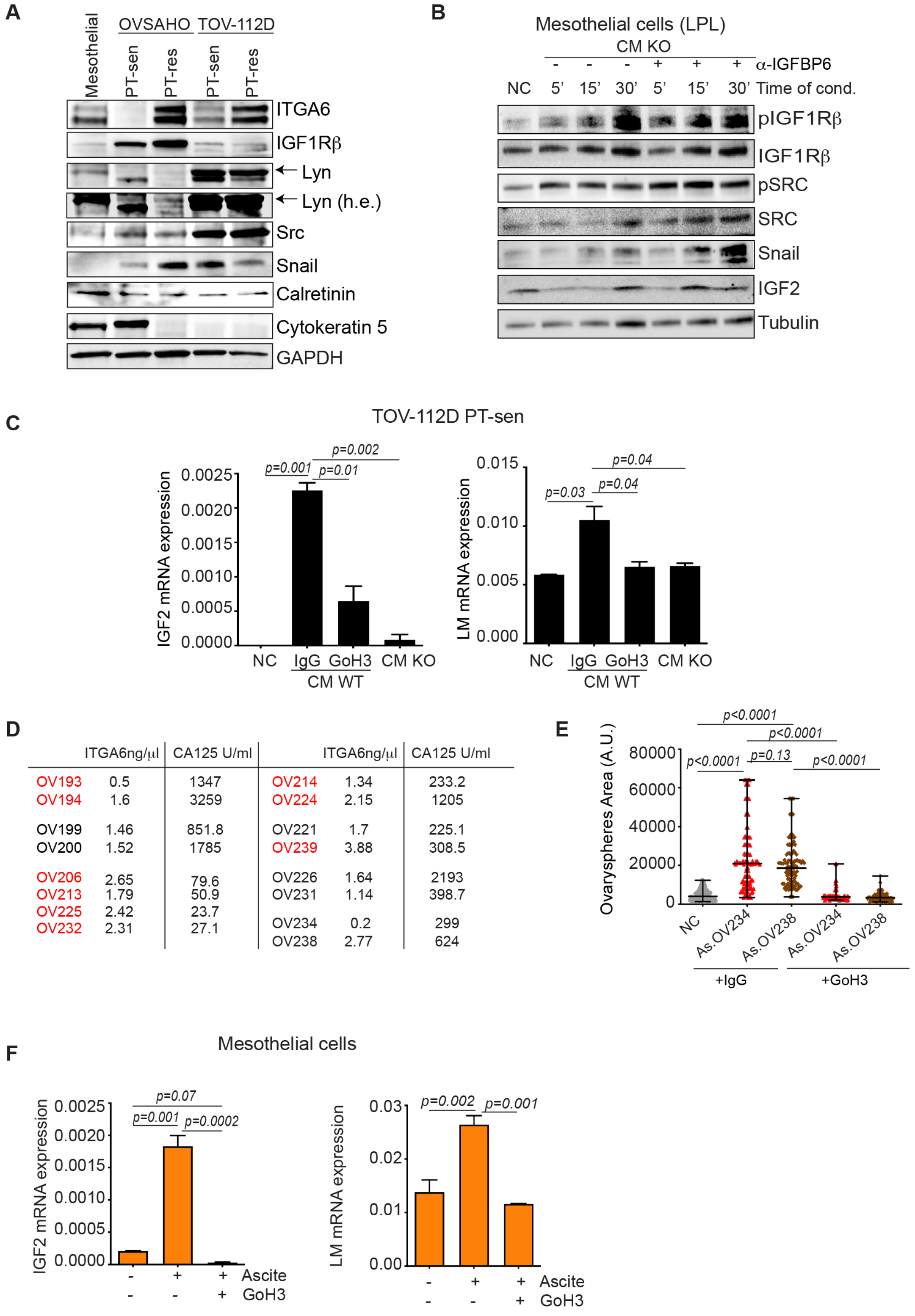

**Appendix Figure S4 (related to Figure 5) Secreted ITGA6 primes mesothelial to form a pro-metastatic microenvironment.**

A) Western blot analysis evaluating the expression of the indicated proteins in whole cell lysates of mesothelial cells, OVSAHO and TOV-112D PT-sen and PT-res. Calretinin and Cytokeratin 5 were used as accepted marker of mesothelial cells and GAPDH as loading control. B) Western blot analysis evaluating the expression of the indicated proteins in whole cell lysates of mesothelial cells conditioned for the indicated times with CM of TOV-112D PT-res ITGA6KO cells in presence or not of an anti-IGFBP6 blocking antibody. Tubulin was used as loading control. C) Graph reporting the mRNA expression of IGF2 and LAMA5 (LM) genes in TOV-112D PT-sen cells challenged with conditioned medium (CM) of PT-res ITGA6 WT in presence or not of the specific anti-ITGA6 GoH3, or with CM of PT-res ITGA6 KO cells. D) Table reporting ITGA6 protein levels in ascitic fluids (measured by ELISA) and CA125 levels in blood samples (measured during clinical follow up) from 7 EOC patients. Samples from PT-res patients are in red. For 6 patients, two samples and for 1 patient, 4 samples, were collected at different times of clinical history. The clinical history of each patient is reported in the Methods section and in Figure 5F. E) Graph reporting the area of ovaryspheres formed by TOV-112D PT-sen cells plated on mesothelial cells monolayer challenged or not with ascites samples of EOC patient n.7 (see Methods) in the presence or not of the specific anti-ITGA6 blocking antibody, GoH3. F) Graph reporting the mRNA expression of IGF2 and LAMA5 (LM) in mesothelial cells incubated with ascites samples described in F in presence or not of the specific anti-ITGA6 blocking antibody, GoH3. In C and F, mRNA expression was analyzed in triplicate and normalized to actin. Statistical significance was determined by a two-tailed, unpaired Student's t-test (Exact $p$ values were reported on graphs).

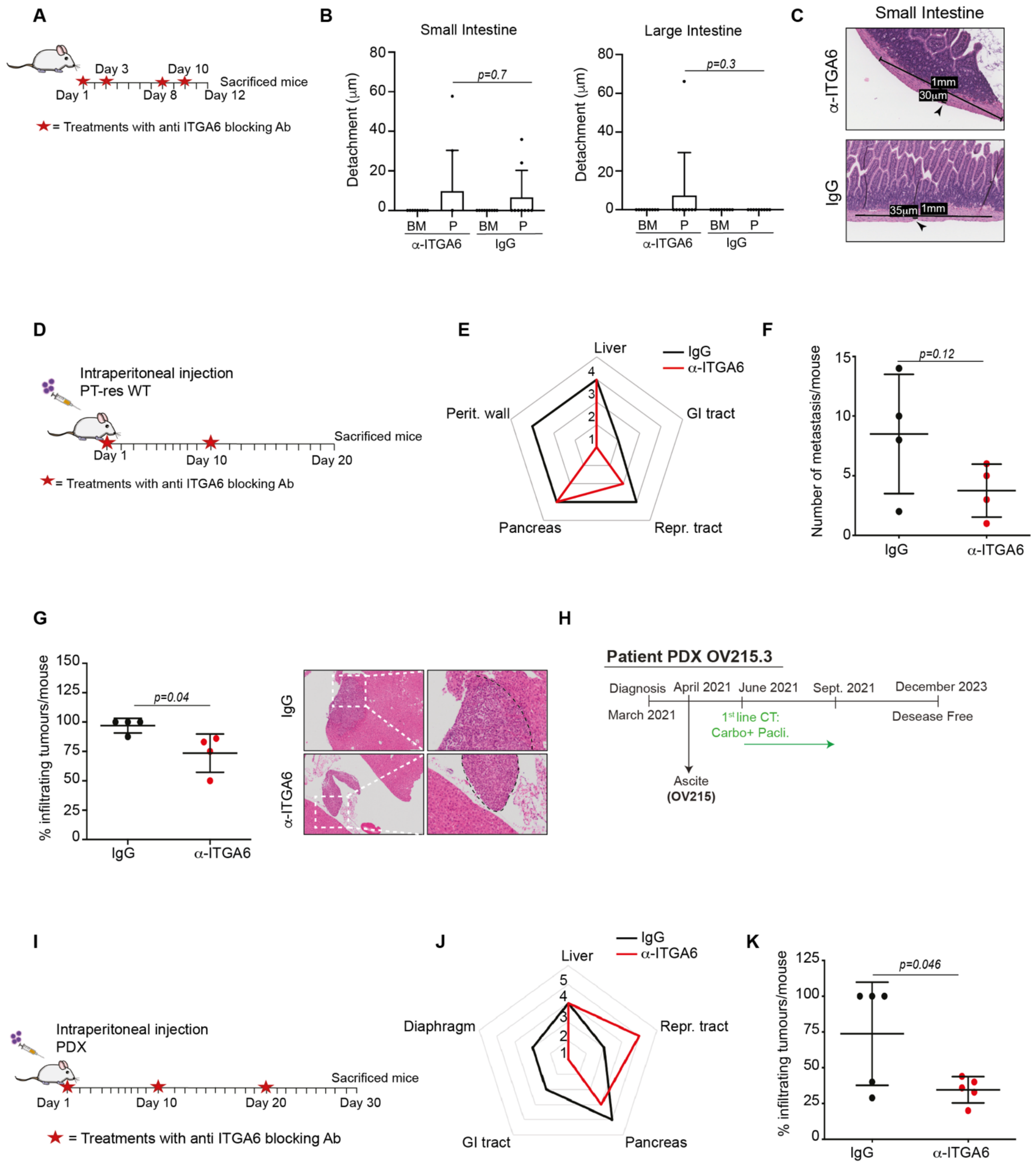


**Appendix figure S5 (related to Figure 6).**

A) Schematic representation of the *in vivo* experimental procedures used. C57BL6 mice were treated with the P5G10 specific anti-ITGA6 blocking antibody (n=3) or with IgG control antibody (n=3) according to the scheme. B-C) Graph (B) and typical H&E images (C) reporting the detachment from basement membrane (BM) or peritoneum (P) of epithelial cells of small and large intestine (see Methods) of mice reported in A. D) Schematic representation of the *in vivo* experimental procedures used. NSG mice injected intraperitoneally (IP) with PT-res ITGA6WT cells (n = 8), were randomly

divided into two group and treated or not with the P5G10 specific anti-ITGA6 blocking antibody, according to the scheme. E-F) Radar (E) and Dot (F) plots reporting the distribution of abdominal metastasis (E) and the total number of metastasis infiltrated in organs of abdomen (F) in mice injected intraperitoneally as in D, as determined by macroscopic and pathological analyses. In E, black and red lines indicate the number (values 0 to 4) of mice affected for each indicated district. J) Graph reporting the percentage of non-infiltrating tumors in mice described in D-F. On the right, typical images of H&E analyses of the liver from mice described in D-F. In P5G10 treated mice PT-res cells after did not infiltrate the liver. For each mouse, 5X and 10X images of the same field are shown. White dashed boxes represent the areas magnified in the right panels. H) Clinical history reporting the timeline of chemotherapy treatments (in green) and ascites collection of EOC patient who donate her ascites to establish PDX OV215.3 (see Methods section) I) Schematic representation of the *in vivo* experimental procedures using EOC PDX model. NSG mice injected IP with PDX OV215.3 (n =10), were randomly divided into two group and treated or not with the specific anti-ITGA6 blocking antibody, P5G according to the scheme. J) Radar plot reporting the distribution of abdominal metastasis in mice injected intraperitoneally as in I, as determined by macroscopic and pathological analyses. Black and red bold lines indicate the number (values 0 to 5) of mice affected for each indicated district. M) Graph reporting the percentage of infiltrating tumors in mice described in I-J. Statistical significance was determined by a two-tailed, unpaired Student's t-test (Exact *p* values were reported on graphs).

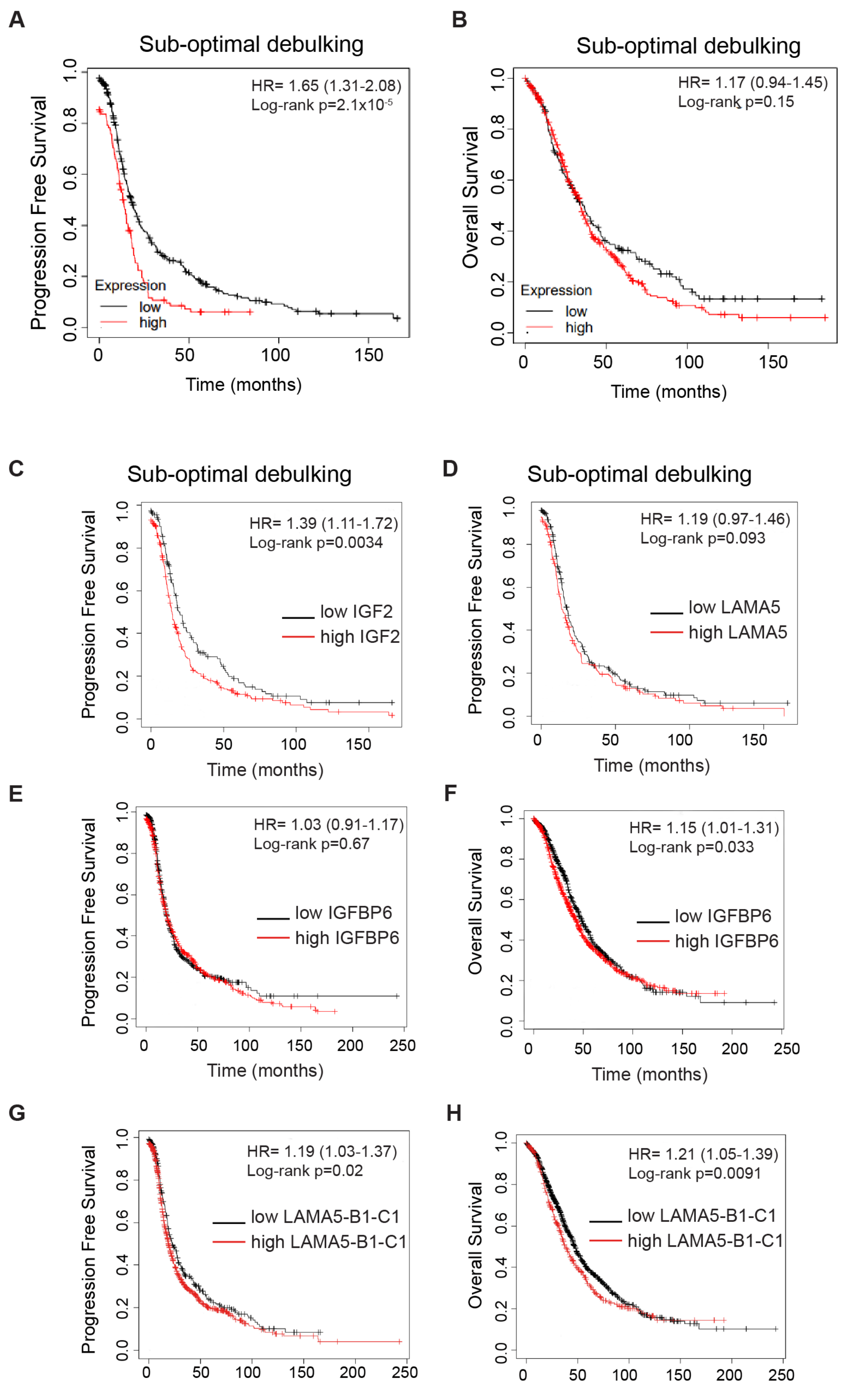


**Appendix figure S6 (related to Figure 7). High ITGA6 expression and activity predict poor prognosis of EOC patients.**

A-B) Kaplan Meyer estimating the progression free (PFS) (n=459) (A) or Overall survival (OS) (n=536) (B) of high risk suboptimal debulked advanced EOC patients, stratified for ITGA6 mRNA expression. C-D) Kaplan Meyer estimating the progression free survival (PFS) of patients described in A and B stratified for IGF2 (C) and LAMA5 (D) expression. E-F) Kaplan Meyer estimating the progression free (PFS) (n=1435) (E) or Overall survival (OS) (n=1656) (F) of EOC patients stratified for IGFBP6 expression. G-H) Kaplan Meyer estimating the progression free (PFS) (n=1435) (E) or Overall survival (OS) (n=1656) (F) of EOC patients stratified for the expression of LM10 components LAMA5B1 and C1 expression.

All Kaplan Meier curves were obtained using the KM Plotter dataset and on-line resource. HR = Hazard Ratio. The Confidence interval is reported between brackets. The p value was calculated using the logrank test.

**Appendix Tables S1. Clinical-pathological features of ovarian cancer patients**

**Appendix Table S1A Clinical-pathological features of 27 ovarian cancer patients who donated their ascites samples (described in Figure 1C)**

| Characteristic | Distribution |
|---|---|
| **Age-Year at diagnosis** | |
| Median | 66 y |
| Range | 44-85 y |
| **Histotype** | |
| Serous | 24 (88.9%) |
| Clear cell | 1 ( 3.7%) |
| Mixed | 1 ( 3.7%) |
| Not Available or Specified | 1 ( 3.7%) |
| **Tumor Stage** | |
| I or II | 0 (0%) |
| III or IV | 25 (92.6%) |
| Not Available or Specified | 2 (7.4%) |
| **Samples Type** | |
| Primary | 16 (47%) |
| Recurrence | 18 (53%) |

**Appendix Table S1B Clinical-pathological features of 30 ovarian cancer patients who donated their tumor samples (described in Figure 2I)**

| Characteristic | Distribution |
|---|---|
| **Age-Year at diagnosis** | |
| Median | 67 y |
| Range | 41-85 y |
| **Histotype** | |
| Serous | 23 (76.7%) |
| Clear cell | 1 (3.3%) |
| Mucinous | 1 (3.3%) |
| Endometrioid | 3 (10%) |
| Not Available or Specified | 2 (6.7%) |
| **Tumor Grade** | |
| G1 or G2 | 3 (10%) |
| G3 | 16 (53.3%) |
| Not Available or Specified | 11 (36.7%) |
| **Samples Type** | |
| Primary | 23 (71.9%) |
| Recurrence | 8 (25%) |
| Not Available or Specified | 1 (3.1%) |

**Appendix Table S2. List of the 71 Human Receptor Tyrosine Kinases (RTKs) evaluated using a phospho-antibody array.**

| Protein | Position 1 in Array | Position 2 in Array | Protein | Position 1 in Array | Position 2 in Array |
|---|---|---|---|---|---|
| ABL1 | G1 | H1 | IGF-I R | K7 | L7 |
| ACK1 | I1 | J1 | Insulin R | A8 | B8 |
| ALK | K1 | L1 | Itk | C8 | D8 |
| Axl | E2 | F2 | JAK1 | E8 | F8 |
| Blk | G2 | H2 | JAK2 | G8 | H8 |
| BMX | I2 | J2 | JAK3 | I8 | J8 |
| Btk | K2 | L2 | LCK | K8 | L8 |
| Csk | A3 | B3 | LTK | A9 | B9 |
| Dtk | C3 | D3 | Lyn | C9 | D9 |
| EGFR | E3 | F3 | MATK | E9 | F9 |
| EphA1 | G3 | H3 | M-CSFR | G9 | H9 |
| EphA2 | I3 | J3 | MUSK | I9 | J9 |
| EphA3 | K3 | L3 | NGFR | K9 | L9 |
| EphA4 | A4 | B4 | PDGFR-α | A10 | B10 |
| EphA5 | C4 | D4 | PDGFR-β | C10 | D10 |
| EphA6 | E4 | F4 | PYK2 | E10 | F10 |
| EphA7 | G4 | H4 | RET | G10 | H10 |
| EphA8 | I4 | J4 | ROR1 | I10 | J10 |
| EphB1 | K4 | L4 | ROR2 | K10 | L10 |
| EphB2 | A5 | B5 | ROS | A11 | B11 |
| EphB3 | C5 | D5 | RYK | C11 | D11 |
| EphB4 | E5 | F5 | SCFR | E11 | F11 |
| EphB6 | G5 | H5 | SRMS | G11 | H11 |
| ErbB2 | I5 | J5 | SYK | I11 | J11 |
| ErbB3 | K5 | L5 | Tec | K11 | L11 |
| ErbB4 | A6 | B6 | Tie-1 | A12 | B12 |
| FAK | C6 | D6 | Tie-2 | C12 | D12 |
| FER | E6 | F6 | TNK1 | E12 | F12 |
| FGFR1 | G6 | H6 | TRKB | G12 | H12 |
| FGFR2 | I6 | J6 | TXK | I12 | J12 |
| FGFR2 (α isoform) | K6 | L6 | Tyk2 | A13 | B13 |
| Fgr | A7 | B7 | TYRO10 | C13 | D13 |
| FRK | C7 | D7 | VEGFR2 | E13 | F13 |
| Fyn | E7 | F7 | VEGFR3 | G13 | H13 |
| Hck | G7 | H7 | ZAP70 | I13 | J13 |
| HGFR | I7 | J7 | | | |

**Appendix Table S3. List of cytokines' expression evaluated using antibodies arrays.**

| Increased in ITGA6KO cells | | | | Decreased in ITGA6KO cells | | |
|---|---|---|---|---|---|---|
| Protein | Mean fold KO/WT | p value | | Protein | Mean fold KO/WT | p value |
| IGFBP6 | 7,60726273 | 0,00259226 | | IGFBP3 | 0,56620282 | 0,03501948 |
| IL-4 | 3,98756299 | 0,03336018 | | ICAM-1 | 0,56868027 | 0,0256188 |
| ANGIOGENIN | 3,16054597 | 0,00013727 | | FLT3 | 0,59413795 | 0,01278056 |
| IGFBP2 | 2,18686592 | 0,01001204 | | ICAM-3 | 0,62159453 | 0,03618497 |
| M-CSF | 1,27102446 | 0,01358263 | | ENA78 | 0,64296381 | 0,03724934 |
| MCP-2 | 1,72252928 | 0,05194512 | | FGF | 0,72552341 | 0,03069529 |
| MCP-4 | 1,40692253 | 0,06405499 | | OPG | 0,73821297 | 0,02964792 |
| SDF1 ALPHA | 3,80355602 | 0,07085209 | | TPO | -0,1998336 | 0,0812738 |
| MDC | 1,23726839 | 0,10236117 | | DTK | 0,4625478 | 0,0991287 |
| IL-2 | 4,29305922 | 0,10908557 | | U PAR | 0,73230461 | 0,11029694 |
| MIP-1 DELTA | 1,22047082 | 0,12563879 | | GRO | 0,5393833 | 0,1142779 |
| RANTES | 1,27908259 | 0,16169678 | | GP130 | 0,68936283 | 0,11514977 |
| CKB | 1,14842809 | 0,1714551 | | IL17A | 0,58166966 | 0,12654988 |
| MIP-3 ALPGA | 1,11217802 | 0,18449984 | | NT4 | 0,78921162 | 0,1288513 |
| CNTF | 1,15477668 | 0,18867736 | | IL2-R ALPHA | 0,53494674 | 0,14121554 |
| FCS | 1,20743884 | 0,20190817 | | BTC | 0,52073699 | 0,15689012 |
| IL-1 RA | 1,14972407 | 0,21368617 | | HCC4 | 0,67579161 | 0,16380249 |
| TARC | 10,1232911 | 0,23359752 | | PLGF | 0,73456174 | 0,1714423 |
| IL-1 BETA | 1,24636819 | 0,28056495 | | EGFR | 0,72194056 | 0,17730985 |
| MCP-3 | 1,54361467 | 0,29283559 | | TNFR II | 0,74950935 | 0,19082892 |
| TNFALPHA | 1,10609835 | 0,30273207 | | TRAIL R3 | 0,59666908 | 0,197282 |
| EOTAXIN 2 | 2,18578346 | 0,3031799 | | IL10 | -0,8809363 | 0,20539987 |
| FRACTALKINE | 1,83099072 | 0,31259744 | | MFP | 0,86170364 | 0,20605745 |
| I 309 | 1,2077951 | 0,37323091 | | FGF7 | 0,50280522 | 0,21779143 |
| TGFB-1 | 1,41431516 | 0,37446202 | | BMP6 | 0,89250222 | 0,25100403 |
| IL12P70 | 2,88069532 | 0,38658551 | | IL1R1 | 0,6543451 | 0,28432238 |
| BDNF | 1,14816513 | 0,42265431 | | FAS | 0,55007343 | 0,28572522 |
| IL-15 | 1,10151066 | 0,46008126 | | IL12 | 0,6095377 | 0,31237351 |
| MIP1-ALPHA | 1,56427029 | 0,52446394 | | GM-CF | 0,64930287 | 0,31241002 |
| TRAILR4 | 1,14515052 | 0,53179388 | | MIP3 BETA | 0,9547971 | 0,33374571 |
| FGF6 | 1,39291529 | 0,55591669 | | TNFR I | 0,88729022 | 0,33922719 |
| EOTAXIN 1 | 1,14568747 | 0,58720008 | | LINFO-TACTIN | 0,71673848 | 0,33968487 |
| IGFBP4 | 1,12148666 | 0,58865929 | | ANGPT2 | 0,73880407 | 0,35520446 |
| GITR LIGAND | 1,30601349 | 0,59351996 | | BLC | 0,88543844 | 0,37097138 |
| VEGFA | 1,23696918 | 0,59520312 | | IL-6 | 0,98026317 | 0,38256769 |
| IL-5 | 1,67547386 | 0,64292036 | | NAP 2 | 0,90334025 | 0,40022551 |
| PARC | 1,24882403 | 0,65785083 | | IL11 | -12,554783 | 0,42385339 |

| | | | | | | |
|---|---|---|---|---|---|---|
| **LIGHT** | 1,22192523 | 0,67209221 | | **IL8** | 0,7202121 | 0,43157637 |
| **MCP-1** | 1,18219289 | 0,76801011 | | **AGRP** | 0,90287898 | 0,43746076 |
| **IL-1 ALPHA** | 4,41377465 | 0,76887339 | | **FGF-9** | 0,61715803 | 0,43919227 |
| **MIF** | 1,36625048 | 0,77971979 | | **GRO ALPHA** | 0,87244127 | 0,4671164 |
| **MIP1 BETA** | 1,45903189 | 0,78277198 | | **HGF** | 0,81689038 | 0,47975854 |
| **OSM** | 1,00771943 | 0,80637878 | | **IL1-R4** | 0,76757434 | 0,51505746 |
| **TGFB-3** | 1,02253113 | 0,81933787 | | **TIMP-2** | -2,3621273 | 0,52452802 |
| **I-TAC** | 1,05211575 | 0,82963791 | | **IGF1** | 0,09618227 | 0,55393744 |
| **ADIPONECTIN** | 1,01142549 | 0,82991505 | | **CTACK** | 0,74240727 | 0,55447907 |
| **TIMP-1** | 1,19550401 | 0,86846005 | | **NT3** | 0,92014971 | 0,56044665 |
| **EGF** | 1,08276751 | 0,87252863 | | **EOTXIN** | 0,64385716 | 0,56760307 |
| **GITR** | 1,02950406 | 0,8879063 | | **AXL** | 0,88849374 | 0,6045616 |
| **IGFBP1** | 1,01018224 | 0,89149755 | | **BETA -NGF** | 0,98074688 | 0,63137257 |
| **IF-GAMMA** | 1,03158786 | 0,90612767 | | **TECK** | 0,62953616 | 0,66818978 |
| **BMP4** | 1,05890457 | 0,90908748 | | **IL-13** | 0,19468073 | 0,71956117 |
| **FGF-4** | 1,09451424 | 0,90964464 | | **IL6-R** | 0,72397634 | 0,73688025 |
| **AR** | 1,05791526 | 0,91340046 | | **IL-7** | 0,87120922 | 0,80539469 |
| **G-CSF** | 1,18503923 | 0,91911604 | | **VEGFD** | -6,5090324 | 0,86187842 |
| **IL-16** | 1,14248323 | 0,92081228 | | **TNF BETA** | 0,97656925 | 0,99057864 |
| **MIG** | 1,04268888 | 0,92688154 | | | | |
| **LEPTIN** | 1,03285899 | 0,92703065 | | | | |
| **PDGF BB** | 1,00911221 | 0,93587066 | | | | |
| **GCP2** | 1,00697823 | 0,93748127 | | | | |
| **GDNF** | 1,02211736 | 0,95240945 | | | | |
| **IGF1R** | 1,0676378 | 0,95495183 | | | | |
| **IL-3** | 1,02049936 | 0,98978723 | | | | |

List of cytokines evaluated in the conditioned medium of ITGA6WT and ITGA6KO TOV112D PT-Res cells.
Data are expressed as the mean ratio of each cytokine levels in the medium of ITGA6KO on its levels in the medium of ITGA6WT cells. The significance ($p<0.05$) of the difference among samples was evaluated by unpaired t-test. Data are the mean of 2 biological replicates each performed in duplicate using an antibody cytokine array (Human Cytokine Array C6-C8 from RayBiotech).

**Appendix Table S4. List and dilutions of Antibodies used.**

| Antibody | Dilution | Sources | Catalog number/identifier |
|---|---|---|---|
| c-Myc | WB 1:1000 | Cell Signaling Technology | 5605; RRID/AB_1903938 |
| c-Myc | WB 1:200 | Santa Cruz Biotechnology | sc-40; RRID/AB_627268 |
| Snail | WB 1:1000 | Cell Signaling Technology | 3879; RRID/AB_2255011 |
| Snail/Slug | IF 1/50 | Abcam | ab180714; RRID/AB_2728773 |
| Slug | WB 1:1000 | Cell Signaling Technology | 9585; RRID/AB_2239535 |
| Phospho p44/42 MAP kinase (T202/Y203) | WB 1:1000 | Cell Signaling Technology | 9101; RRID/AB_331646 |
| ERK1 | WB 1:300 | Santa Cruz Biotechnology | sc-271269; RRID/AB_10611091 |
| Phospho-Akt (S473) | WB 1:2000 | Cell Signaling Technology | 9271; RRID/AB_329825 |
| Akt | WB 1:500 | Cell Signaling Technology | 9272; RRID/AB_329827 |
| Phospho-Lyn (Y507) | WB 1:1000 | Cell Signaling Technology | 2731; RRID/AB_2138262 |
| Phospho Lyn (Y397) | WB 1:1000 | Abcam | ab226778; RRID/AB_2928952 |
| Lyn | WB 1:1000 | Cell Signaling Technology | 2796; RRID/AB_2138391 |
| TCF8/ZEB1 | WB 1:1000 | Cell Signaling Technology | 3396; RRID/AB_1904164 |
| Phospho-IGF-I Receptor beta (Y1135) | IF 1/50; WB 1:1000 | Cell Signaling Technology | 3918; RRID/AB_10548764 |
| IGF-I Receptor beta | WB 1:500 | Cell Signaling Technology | 9750; RRID/AB_10950969 |
| Phospho-mTOR (S2448) | WB 1:1000 | Cell Signaling Technology | 2971; RRID/AB_330970 |
| mTOR | WB 1:500 | Cell Signaling Technology | 2983; RRID/AB_2105622 |
| Phospho-Src Family (Y416) | WB 1:500 | Cell Signaling Technology | 2101; RRID/ AB_331697 |
| c-Src | WB 1:500 | Santa Cruz Biotechnology | sc-5266; RRID/AB_627308 |
| 4E-BP1 | WB 1:1000 | Cell Signaling Technology | 9644; RRID/AB_2097841 |
| Anti-4E-BP1, phospho (T37/T46) | WB 1:1000 | Cell Signaling Technology | 2855; RRID/AB_560835 |
| Vinculin | WB 1:500 | Santa Cruz Biotechnology | sc-7649; RRID/AB_2288413 |
| alpha Tubulin | WB 1:500 | Santa Cruz Biotechnology | sc-32293; RRID/AB_628412 |
| Twist | WB 1:200 | Santa Cruz Biotechnology | sc-81417; RRID/AB_1130910 |
| ITGA6 | WB 1:1000 | Merk-Sigma | APREST78051; RRID/AB_2928950 |
| ITGA6 | WB 1:1000 | Cell Signaling Technology | 3750; RRID/AB_2249263 |
| SP1 | WB 1:500 | Merk-Sigma | SAB1404397; RRID/AB_10759217 |
| HDAC1 | WB 1:1000 | Merk-Sigma | H3284; RRID/AB_260039 |
| GAPDH | WB 1:1000 | Millipore | CB1001, RRID/AB_2107426 |
| IRS1 | WB 1:1000 | Millipore | 05-1085; RRID/AB_1977296 |
| IGF-II | WB 1:1000 | R&D | 792-MG; RRID/AB_2928954 |

| | | | |
|---|---|---|---|
| IGFBP6 | In vitro 10 µg/ml WB 1:500 | R&D | AF876; RRID/AB_355679 |
| Phospho-FAK (Y397) | WB 1:1000 | Thermo Fisher Scientific | 44-624G, RRID/AB_2533701 |
| FAK | WB 1:1000 | BD Biosciences | 610087, RRID/AB_397494 |
| PE Anti-CD49a | FACS 10ml/1x10$^6$cells | BD Biosciences | 559596, RRID/AB_397288 |
| PE Anti-CD49b | FACS 10ml/1x10$^6$cells | BD Biosciences | 553858, RRID/AB_395094 |
| APC Anti-CD49d | FACS 10ml/1x10$^6$cells | BD Biosciences | 561892, RRID/AB_10896134 |
| PE Anti-CD49e | FACS 10ml/1x10$^6$cells | BD Biosciences | 555617, RRID/AB_395984 |
| PE Anti-CD49f | FACS 10ml/1x10$^6$cells | BD Biosciences | 555736, RRID/AB_396079 |
| PE Anti-CD51 | FACS 10ml/1x10$^6$cells | BD Biosciences | 551187, RRID/AB_394088 |
| PE Anti-CD29 | FACS 10ml/1x10$^6$cells | BD Biosciences | 557332, RRID/AB_396646 |
| PE Anti-CD61 | FACS 10ml/1x10$^6$cells | BD Biosciences | 555754, RRID/AB_396095 |
| PE Anti-CD104 | FACS 10ml/1x10$^6$cells | BD Biosciences | 555720, RRID/AB_396063 |
| Goat anti-Mouse IgG2b Antibody HRP-Conj. | WB 1:5000 | Bethyl | A90-109P, RRID/AB_67160 |
| Goat anti-Rabbit IgA Antibody HRP-Conj. | WB 1:5000 | Bethyl | A120-109P, RRID/AB_67251 |
| Purified anti-human/ mouse CD49f (GoH3) | In vitro 10 µg/ml; | BioLegend | 313602, RRID/AB_345296 |
| Purified Rat IgG2a, κ Isotype Ctrl Antibody | FACS 10ml/1x10$^6$cells | BioLegend | 400501, RRID/AB_326523 |
| Integrin alpha-6, human blocking Ab (P5G10) | In Vivo 30mg/kg IF 3mg/ml | DSHB | P5G10, RRID/AB_2619593 |
| Alexa Fluor™ 647 Phalloidin | IF 1:200 | Invitrogen | A22287 |
| TO-PRO-3 Iodide (642/661) | IF 1:500 | Invitrogen | T3605 |
| IRDye® 680RD Goat anti-Mouse IgG Sec. Ab | WB 1:1500 | LI-COR | 926–68070, RRID/ AB_10956588 |
| IRDye® 680RD Goat anti-Rabbit IgG Sec. Ab | WB 1:1500 | LI-COR | 926–68071; RRID/ AB_10956166 |
| IRDye® 800CW Goat anti-Mouse IgG Sec. Ab | WB 1:1500 | LI-COR | 926–32210; RRID/ AB_621842 |
| IRDye® 800CW Goat anti-Rabbit IgG Sec. Ab | WB 1:1500 | LI-COR | 926–32211; RRID/ AB_621843 |

**Appendix Table S5. List of Oligonucleotides used for RT-PCR and ChIP analysis**

| Oligo Name | Sequence |
|---|---|
| ITGA6 promoter (FL) FW | *CTCGAG*TTTTGAGGGTTGTTAGG |
| ITGA6 promoter Rev | *AGATCT*GGGTCGCGCTCTCCGTCCGG |
| ITGA6 promoter (MUT1) FW | *CTCGAG*GGCCACATCTGCAAG |
| gITGA6-3 FW | GGGCTGCAGTTGCCAGTGCA |
| gITGA6-3 Rev | TGCACTGGCAACTGCAGCCC |
| gITGA6-6 FW | AGAAGCCGAAGAGGCTCCCG |
| gITGA6-6 Rev | CGGGAGCCTCTTCGGCTTCT |
| ITGA6 for ChIP FW | TTGTGTACATTATACAGCAC |
| ITGA6 for ChIP Rev | GGAGACTTTACTACCCGTTATAAA |
| ITGA6 for qRT-PCR FW | AAGTCTCGTTCCTGTTCCTGC |
| ITGA6 for qRT-PCR Rev | ACTGTGATTGGCTCTGGGAG |
| ITGA6A for qRT-PCR FW | GAGATCCATGCTCAGCCATC |
| ITGA6A for qRT-PCR Rev | ACTGTCATCGTACCTAGAGCGTTT |
| ITGA6B for qRT-PCR FW | ACTATGGAAGTGTGGATTCTTT |
| ITGA6B for qRT-PCR Rev | CCCCCGCTATGAGTAGCTTT |
| Actin for qRT-PCR FW | CCAGAGGCGTACAGGGATAG |
| Actin for qRT-PCR Rev | CCAACCGCGAGAAGATGA |
| SP1 for qRT-PCR FW | GGTGCCTTTTCACAGGCTC |
| SP1 for qRT-PCR Rev | CATTGGGTGACTCAATTCTGCT |
| MYC for qRT-PCR FW | CTCCTGGCAAAAGGTCAGAG |
| MYC for qRT-PCR Rev | TCGGTTGTTGCTGATCTGTC |
| LAMA5 for qRT-PCR FW | GACTGCCAACAGTGCCAAC |
| LAMA5 for qRT-PCR Rev | CCACCCTGATAGGTGCCAT |
| IGF-II for qRT-PCR FW | GAAGATGCTGCTGTGCTTCC |
| IGF-II for qRT-PCR Rev | AGTGAGCAAAACTGCCGC |
| IGFBP6 for qRT-PCR FW | AATCCAGGCACCTCTACCA |
| IGFBP6 for qRT-PCR Rev | GCCCATCCGATCCACA |
