# Supplementary figures and images for: Platinum-induced upregulation of ITGA6 promotes chemoresistance and spreading in ovarian cancer
Source: EMBO Mol Med. 2024 Apr 24;16(5):7. doi: 10.1038/s44321-024-00069-3 (PMC11099142; doi:10.1038/s44321-024-00069-3)

## Slide 1
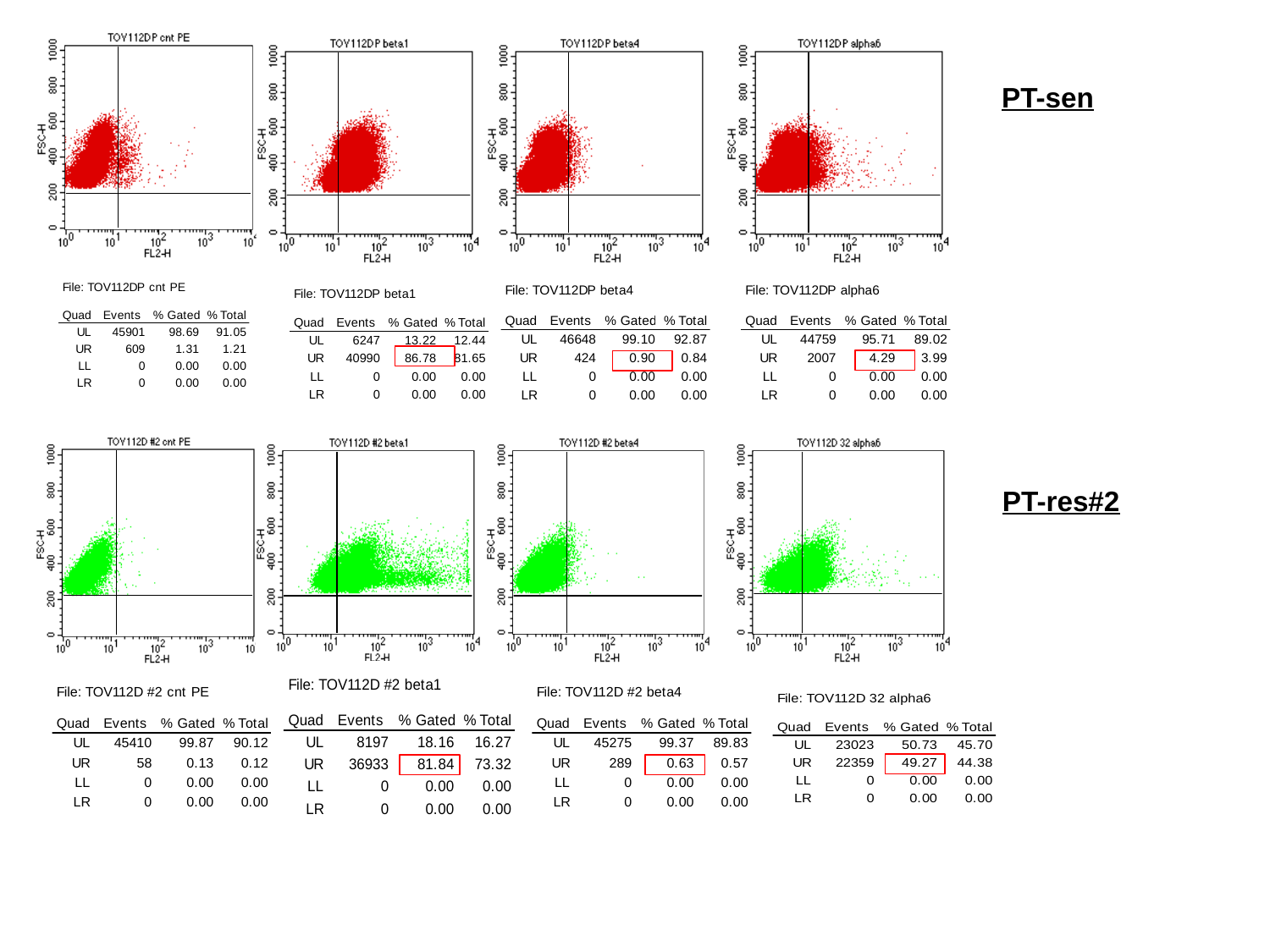

PT-sen
PT-res#2

## Slide 2
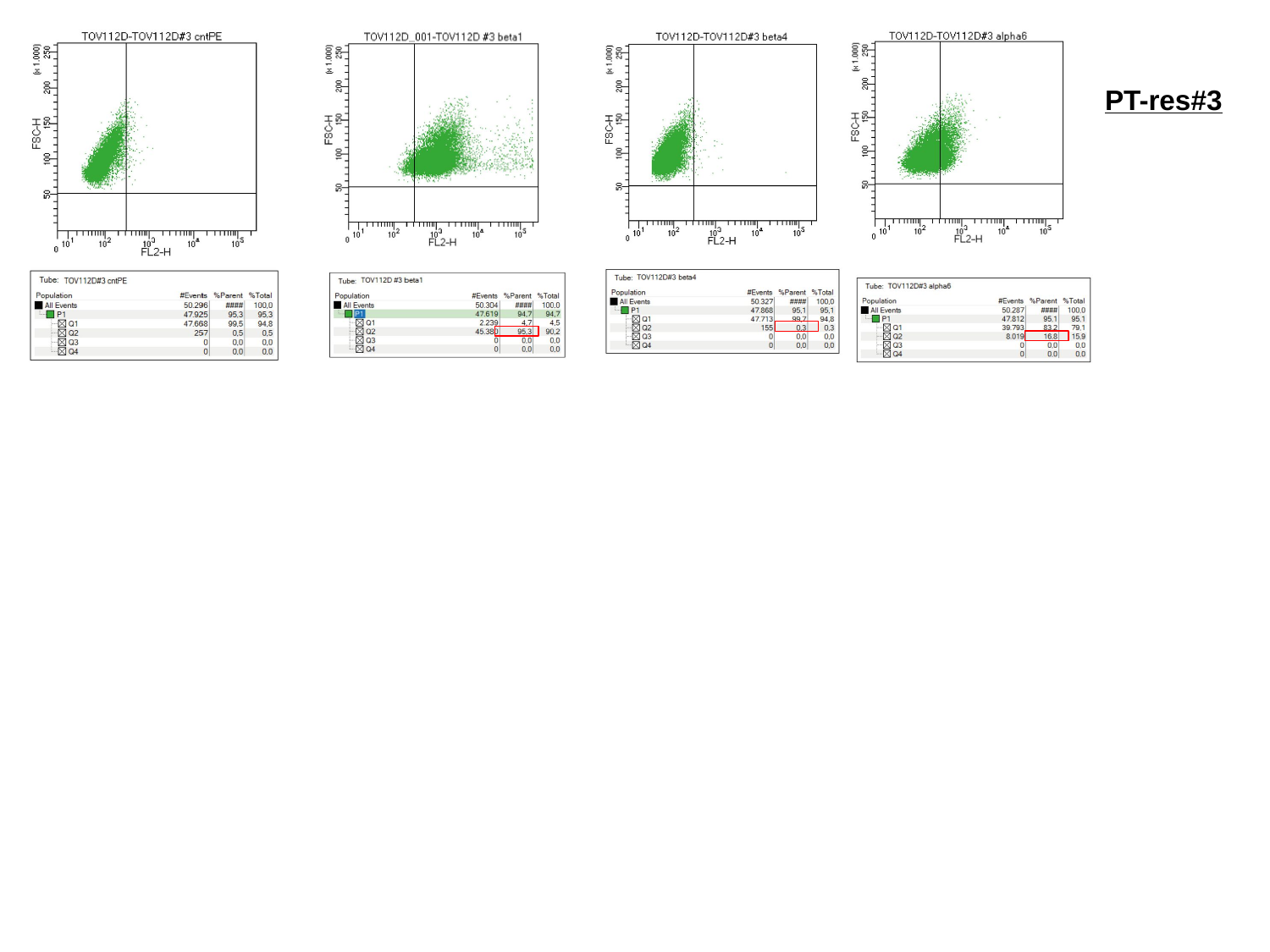

PT-res#3

Supplement: Supplementary file 2 — Source data Fig. 1 [file 44321_2024_69_MOESM2_ESM.zip › Figure 1/FACS Fig.1A.pptx]

**Figure 1**

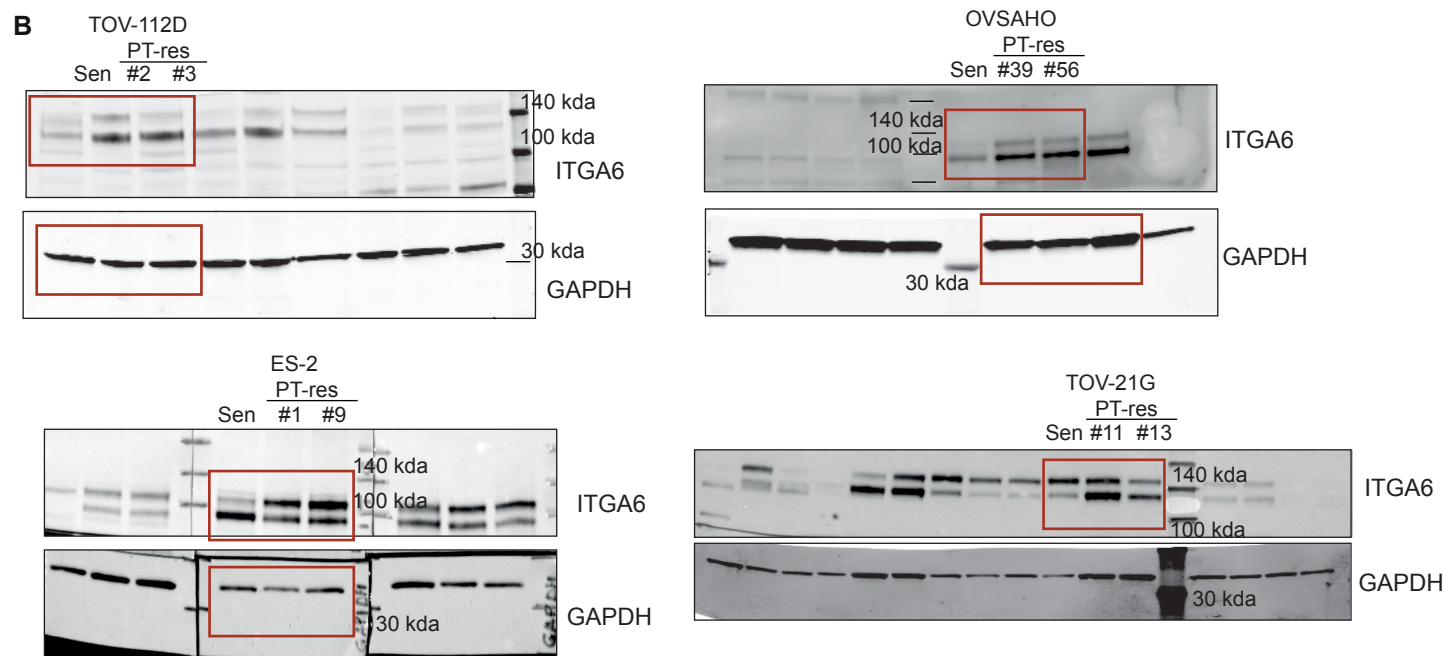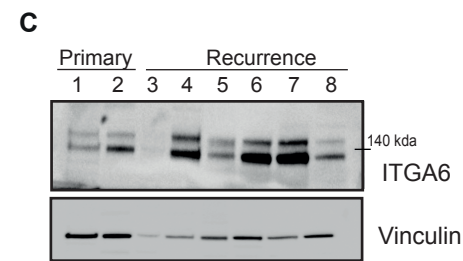

**Figure 2**

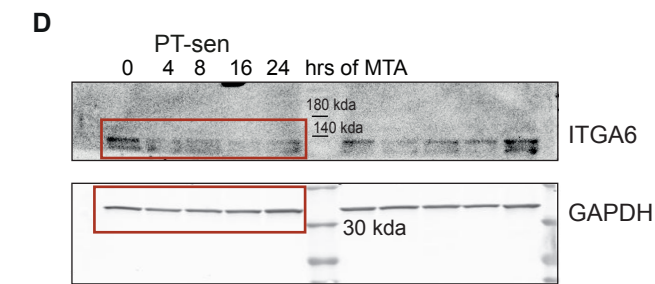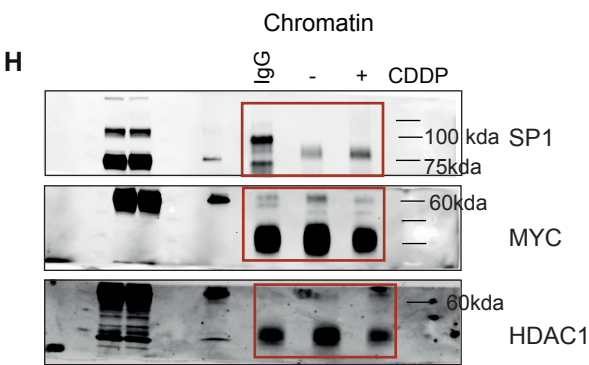

**Figure 3**

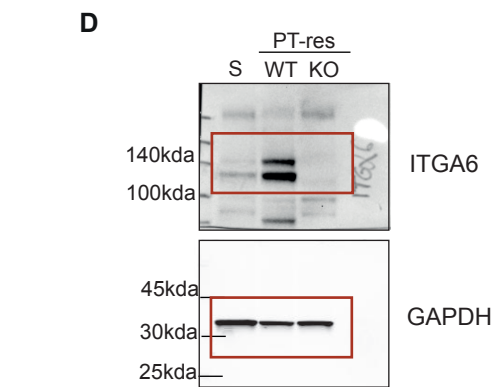

Supplement: Supplementary file 2 — Source data Fig. 1 [file 44321_2024_69_MOESM2_ESM.zip › Figure 1/Figure1-2-3 source data.pdf]

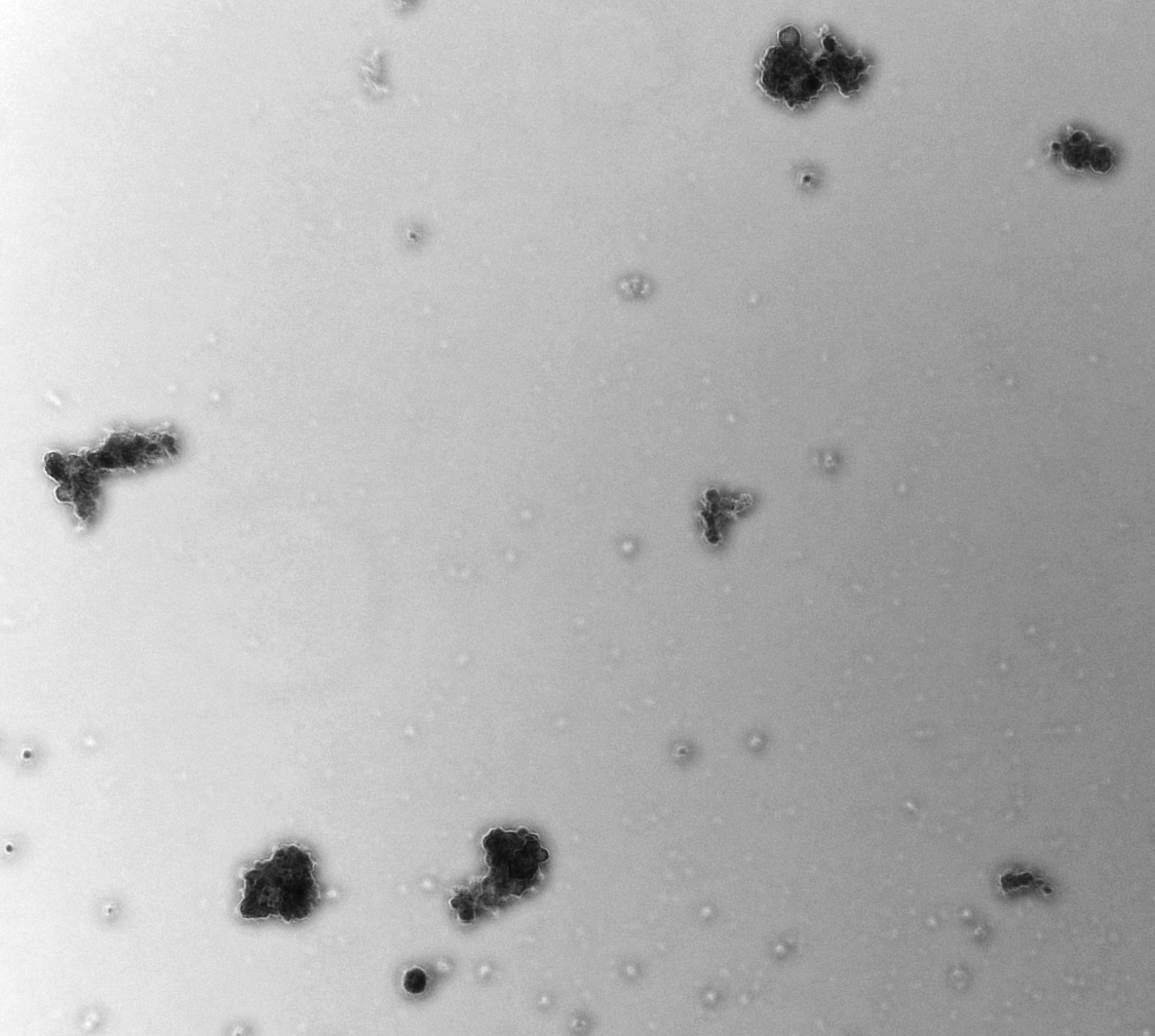

Supplement: Supplementary file 4 — Source data Fig. 3 [file 44321_2024_69_MOESM4_ESM.zip › Figure 3/IMAGES for Fig.3F/PT-res ITGA6KO UNT.tif]

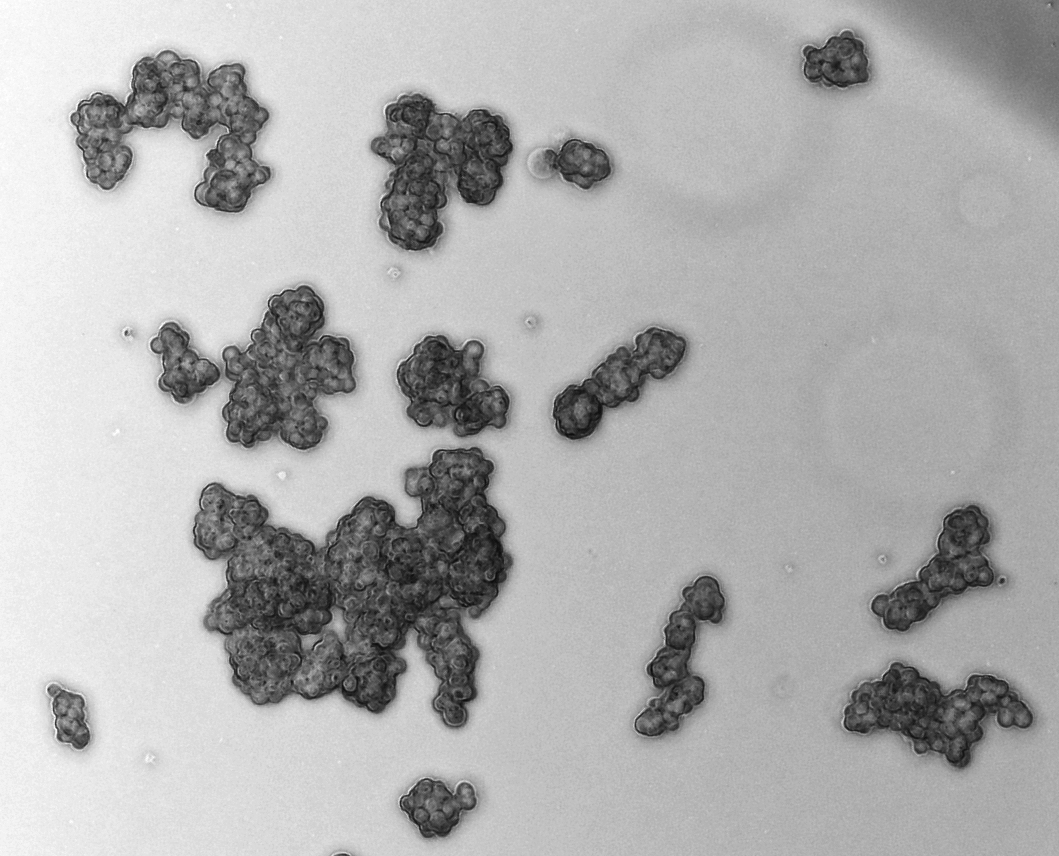

Supplement: Supplementary file 4 — Source data Fig. 3 [file 44321_2024_69_MOESM4_ESM.zip › Figure 3/IMAGES for Fig.3F/PT-res UNT .tif]

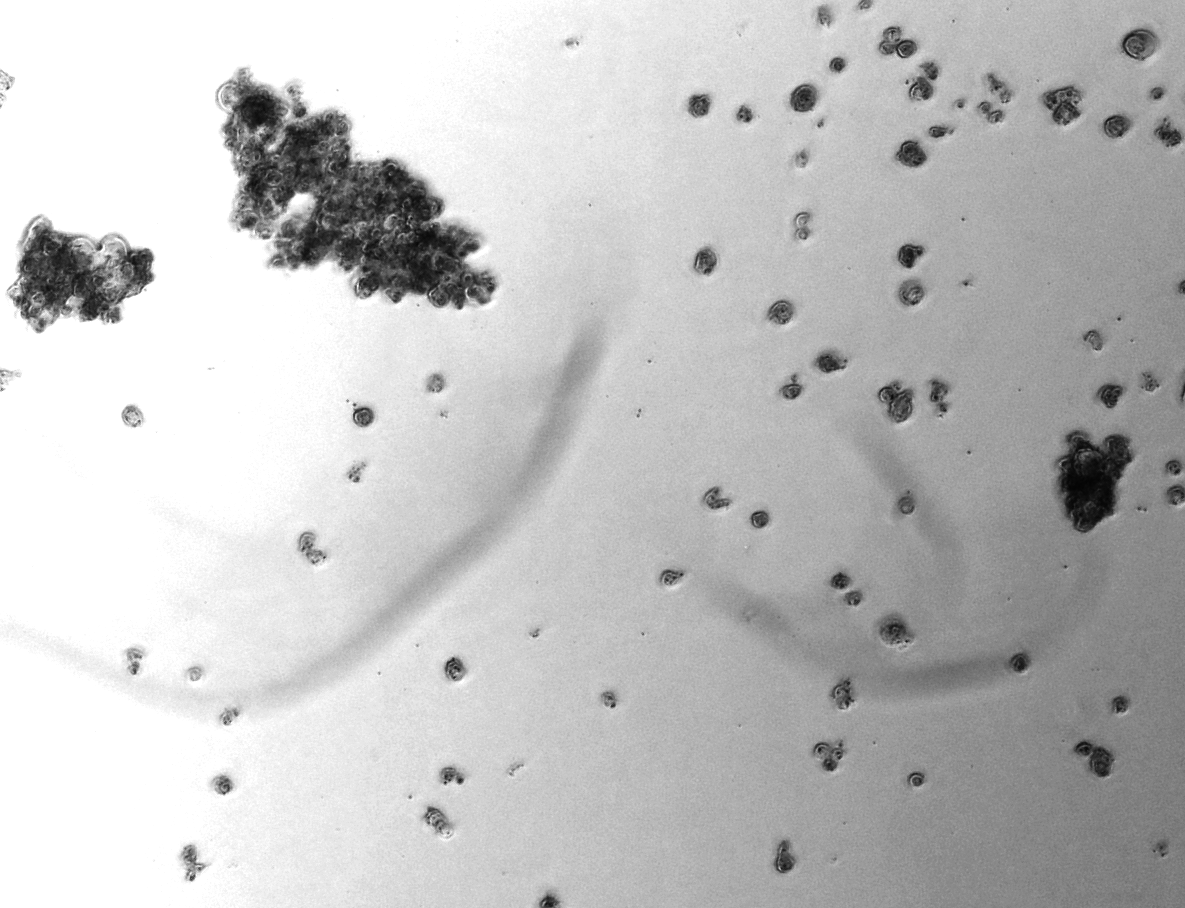

Supplement: Supplementary file 4 — Source data Fig. 3 [file 44321_2024_69_MOESM4_ESM.zip › Figure 3/IMAGES for Fig.3F/PT-sen cddp.tif]

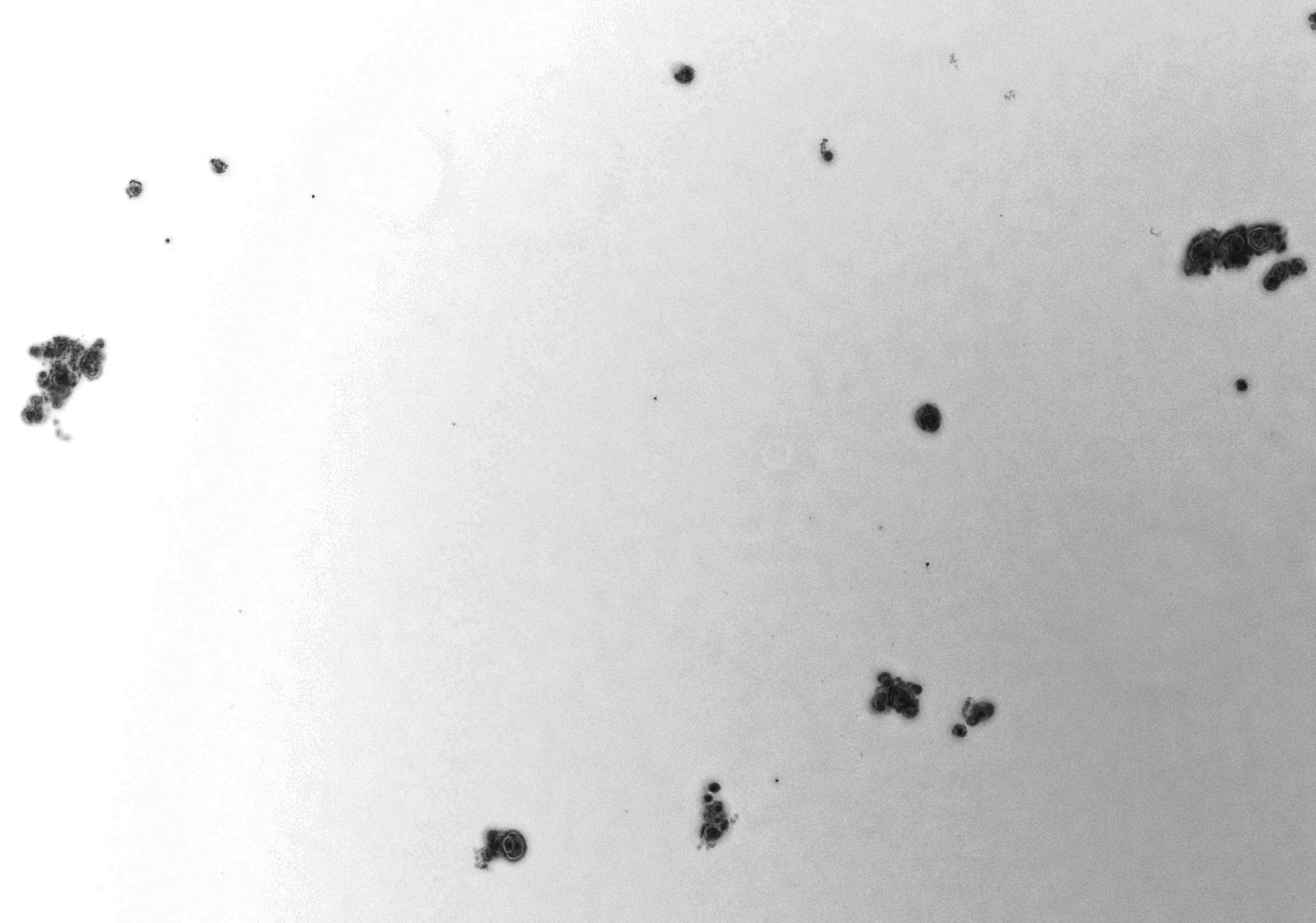

Supplement: Supplementary file 4 — Source data Fig. 3 [file 44321_2024_69_MOESM4_ESM.zip › Figure 3/IMAGES for Fig.3F/PT-res ITGA6KO cddp .tif]

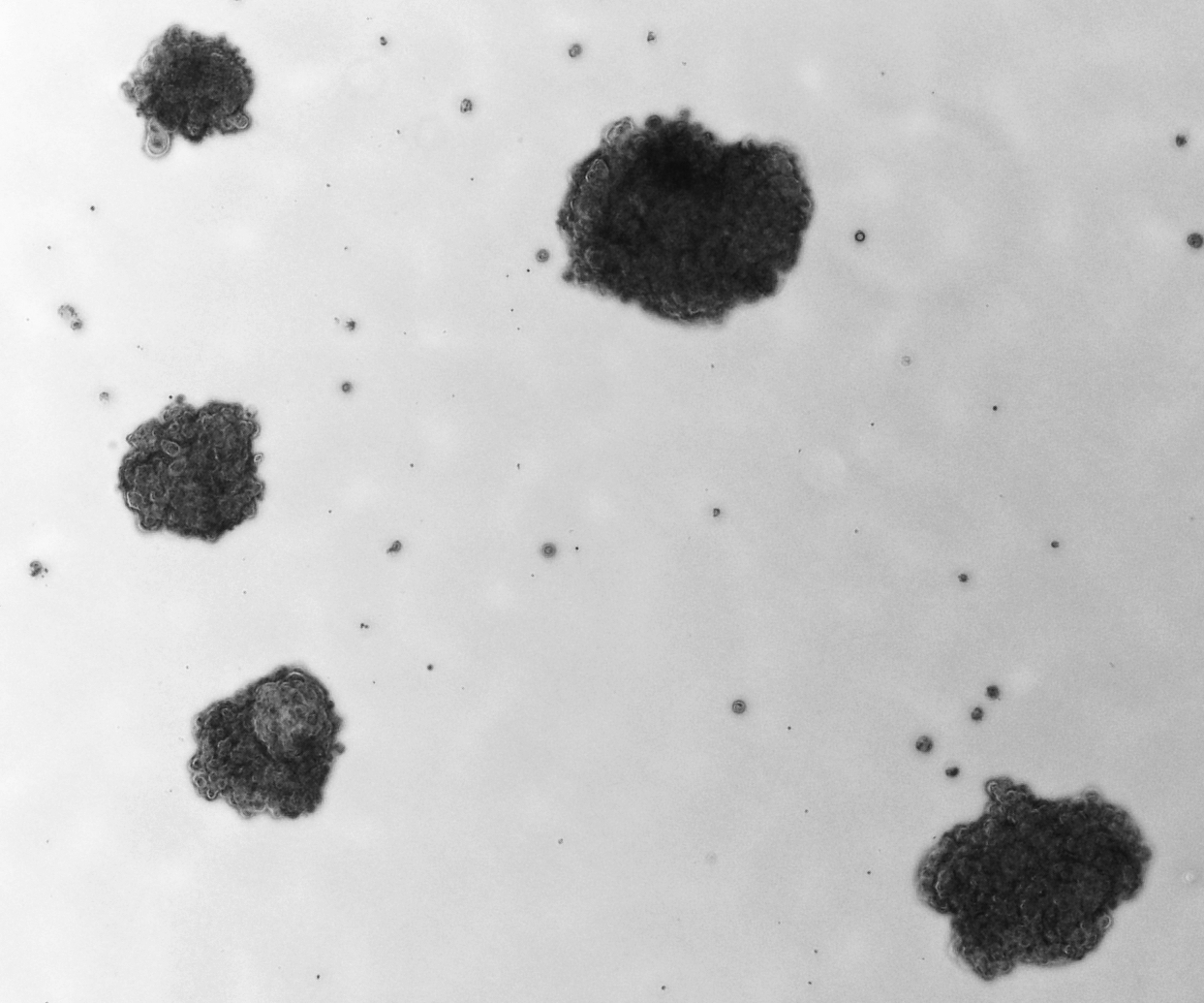

Supplement: Supplementary file 4 — Source data Fig. 3 [file 44321_2024_69_MOESM4_ESM.zip › Figure 3/IMAGES for Fig.3F/PT-sen UNT.tif]

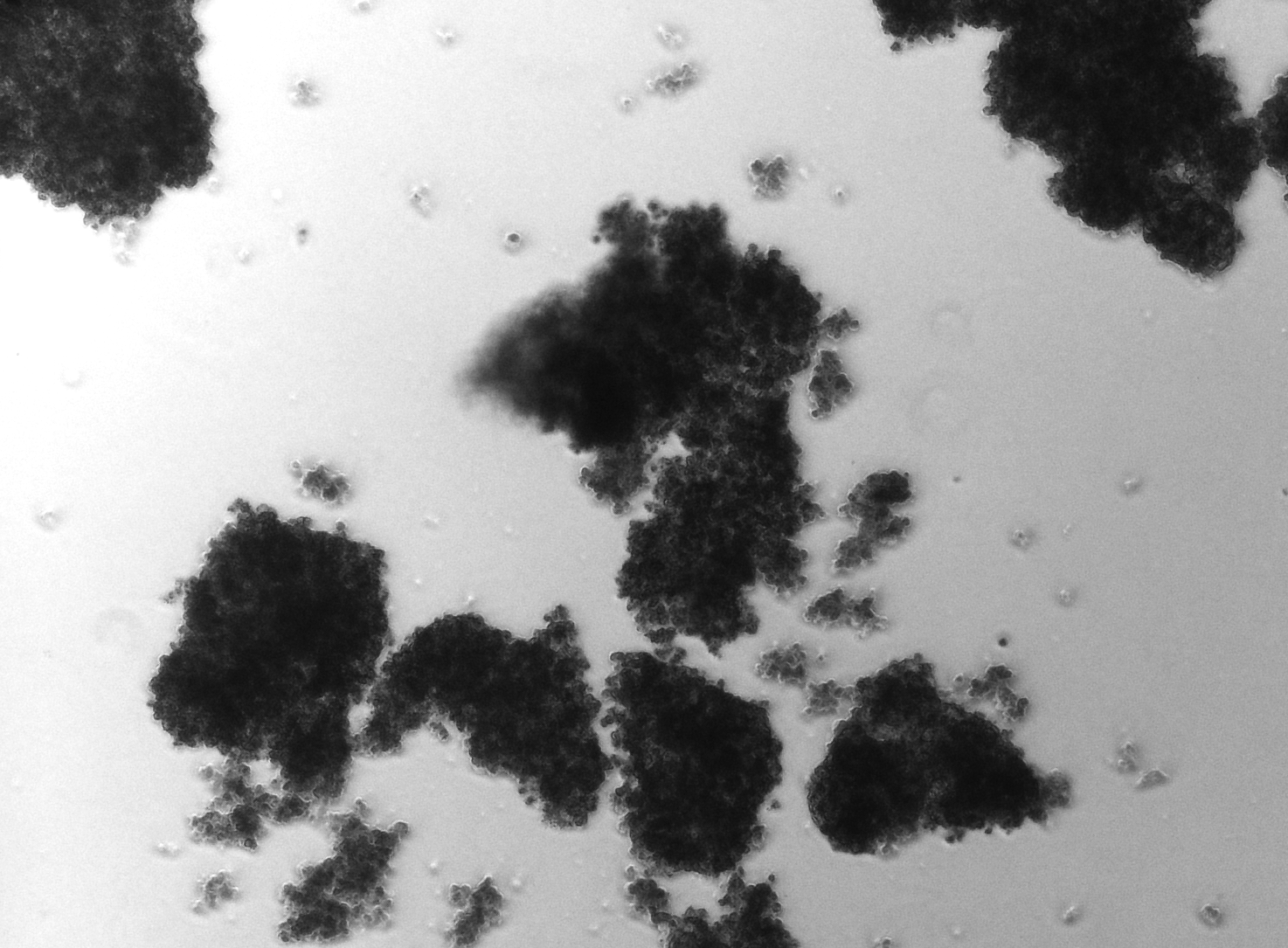

Supplement: Supplementary file 4 — Source data Fig. 3 [file 44321_2024_69_MOESM4_ESM.zip › Figure 3/IMAGES for Fig.3F/PT-res cddp .tif]

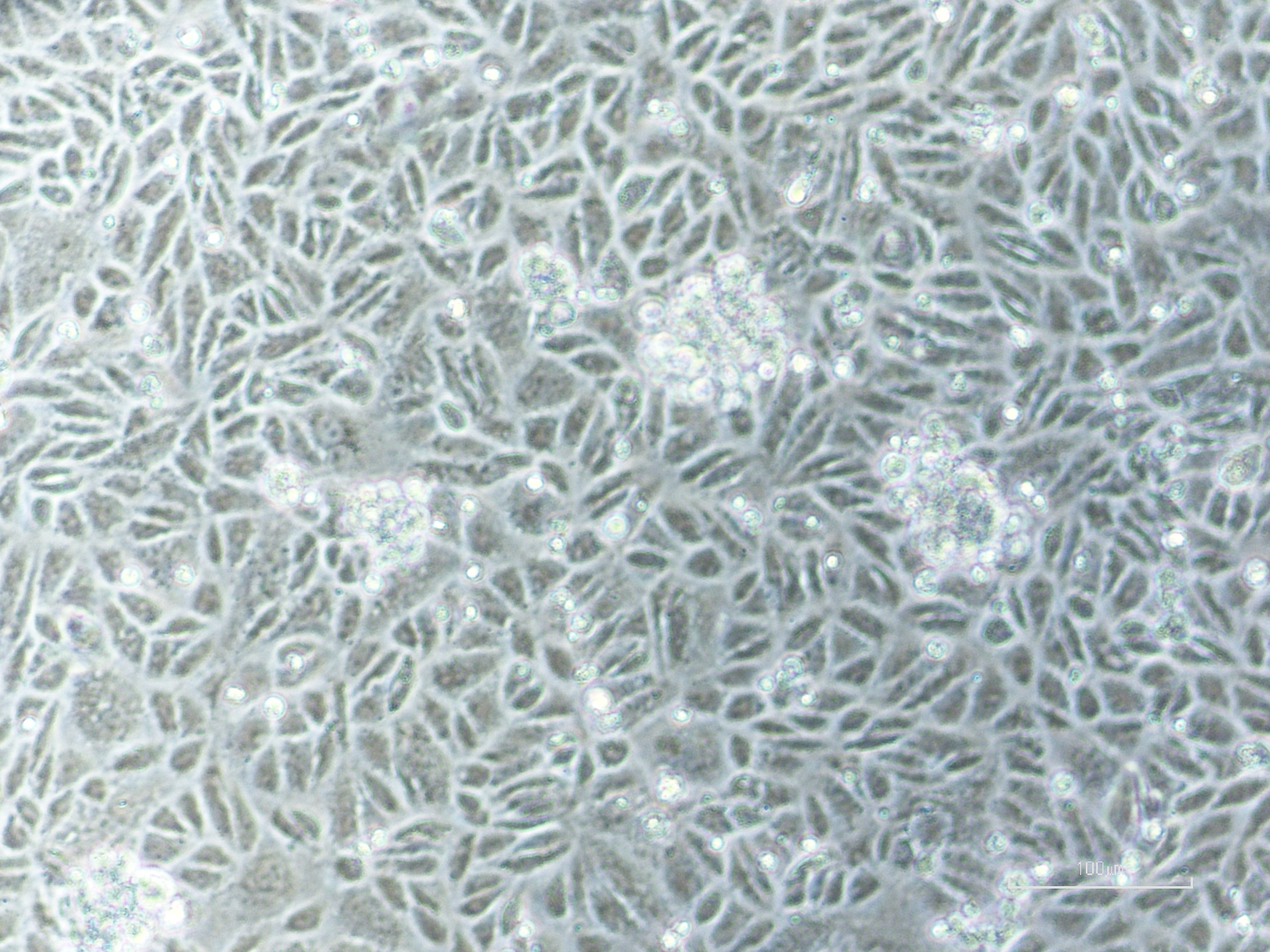

Supplement: Supplementary file 4 — Source data Fig. 3 [file 44321_2024_69_MOESM4_ESM.zip › Figure 3/IMAGES for Fig.3G/PT-sen.TIF]

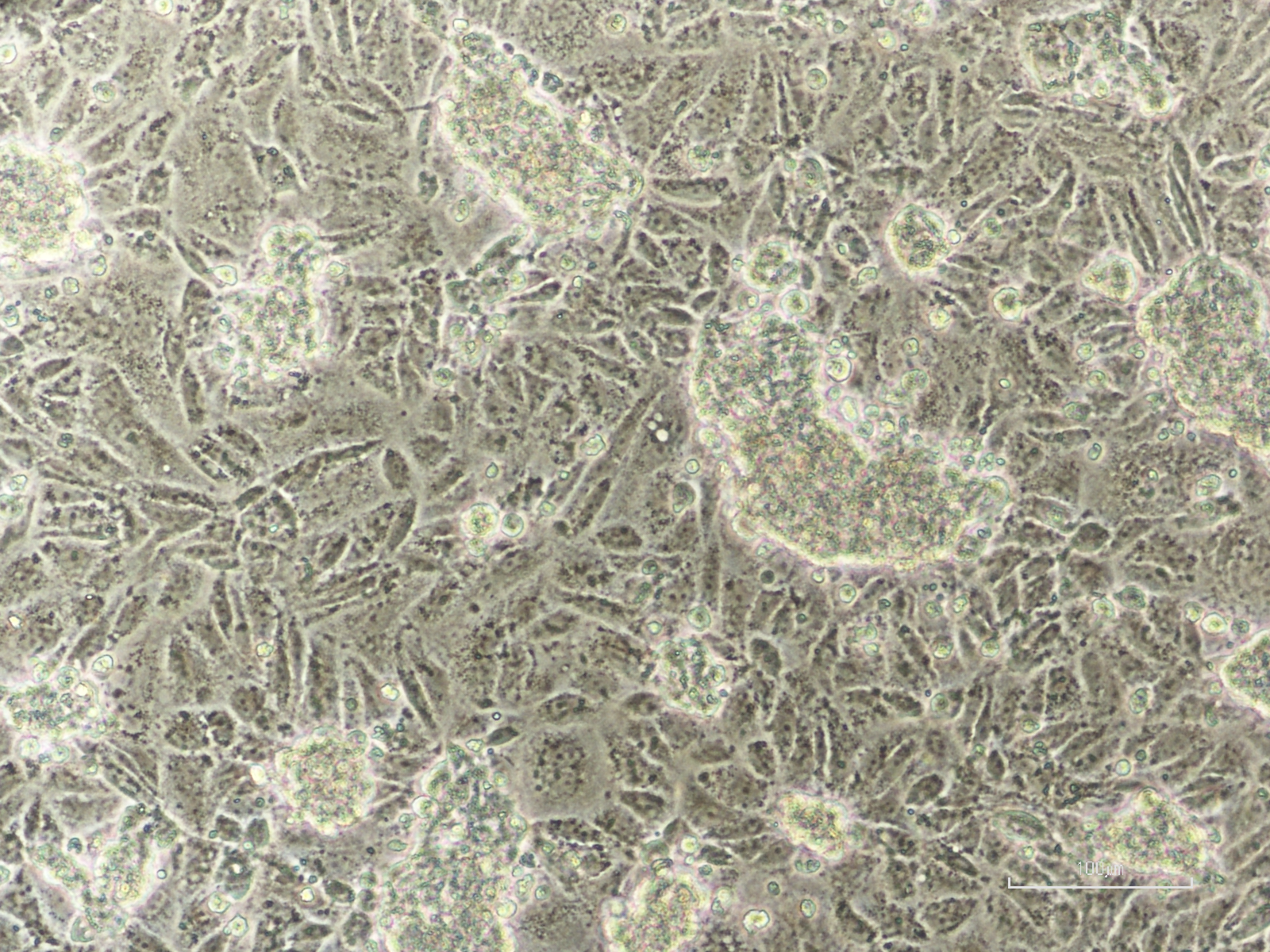

Supplement: Supplementary file 4 — Source data Fig. 3 [file 44321_2024_69_MOESM4_ESM.zip › Figure 3/IMAGES for Fig.3G/PT-res.TIF]

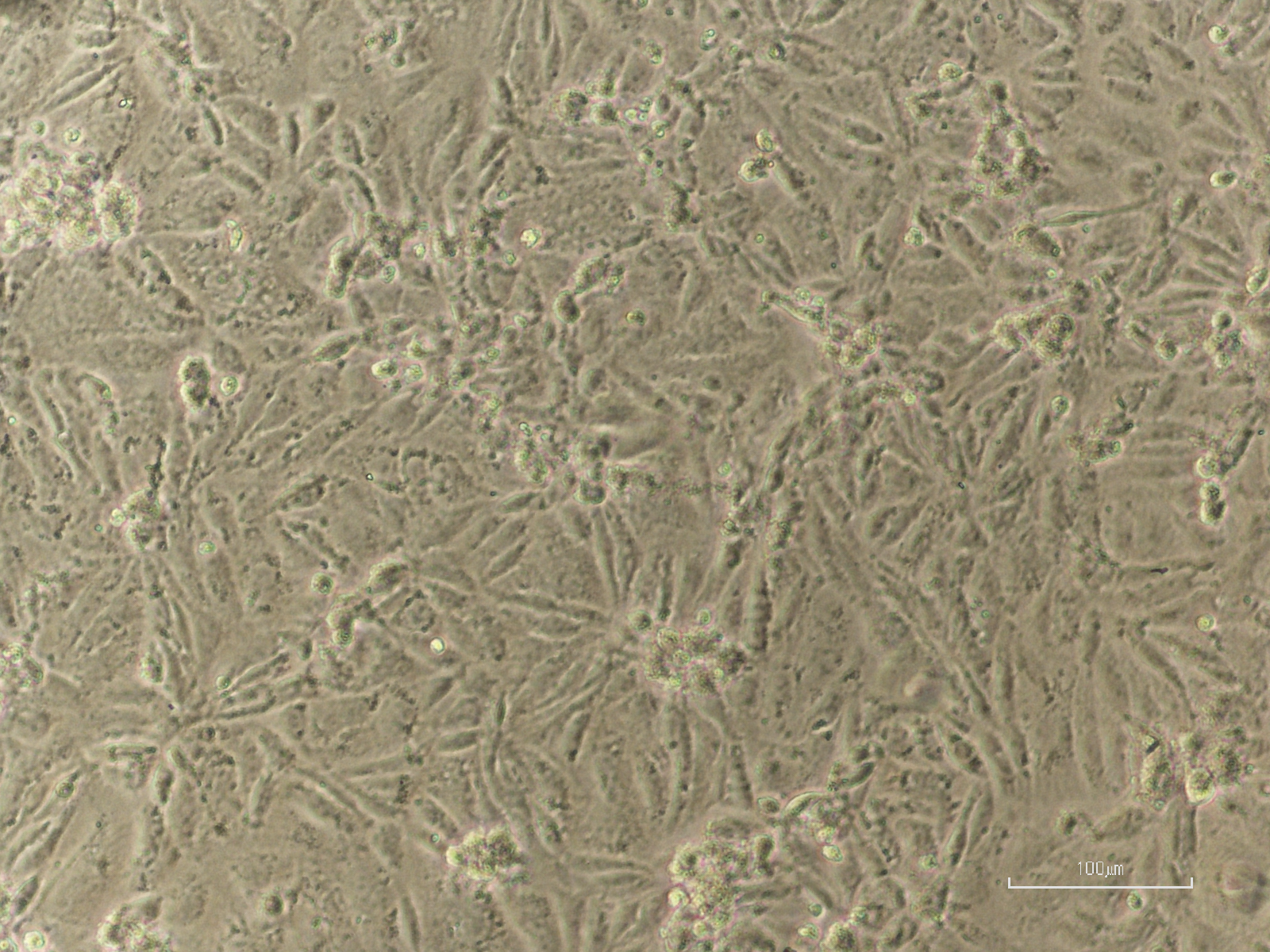

Supplement: Supplementary file 4 — Source data Fig. 3 [file 44321_2024_69_MOESM4_ESM.zip › Figure 3/IMAGES for Fig.3G/PT-res ITGA6 KO.TIF]

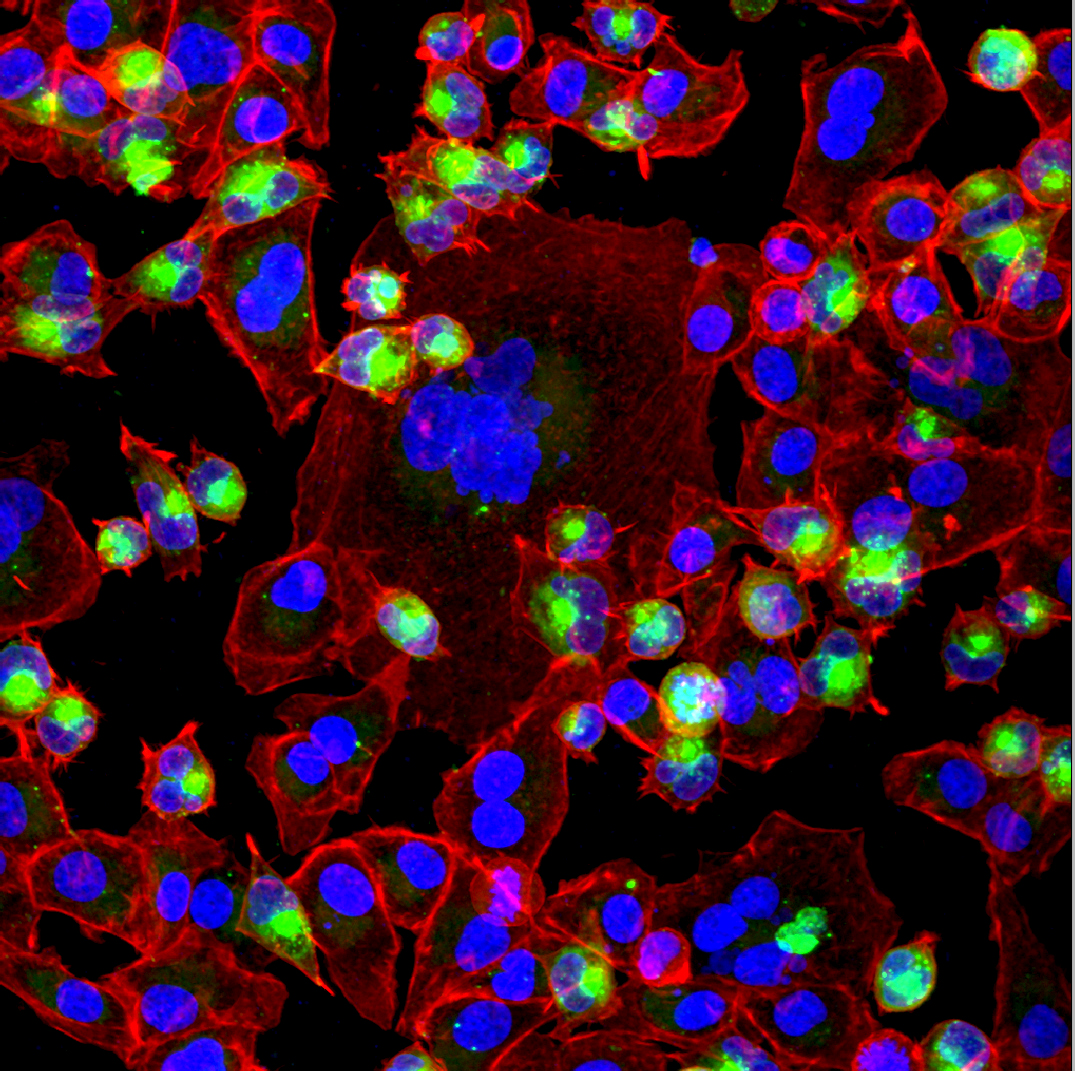

Supplement: Supplementary file 4 — Source data Fig. 3 [file 44321_2024_69_MOESM4_ESM.zip › Figure 3/IMAGES for Fig.3C/TOV PT-res Ig ctrl.jpg]

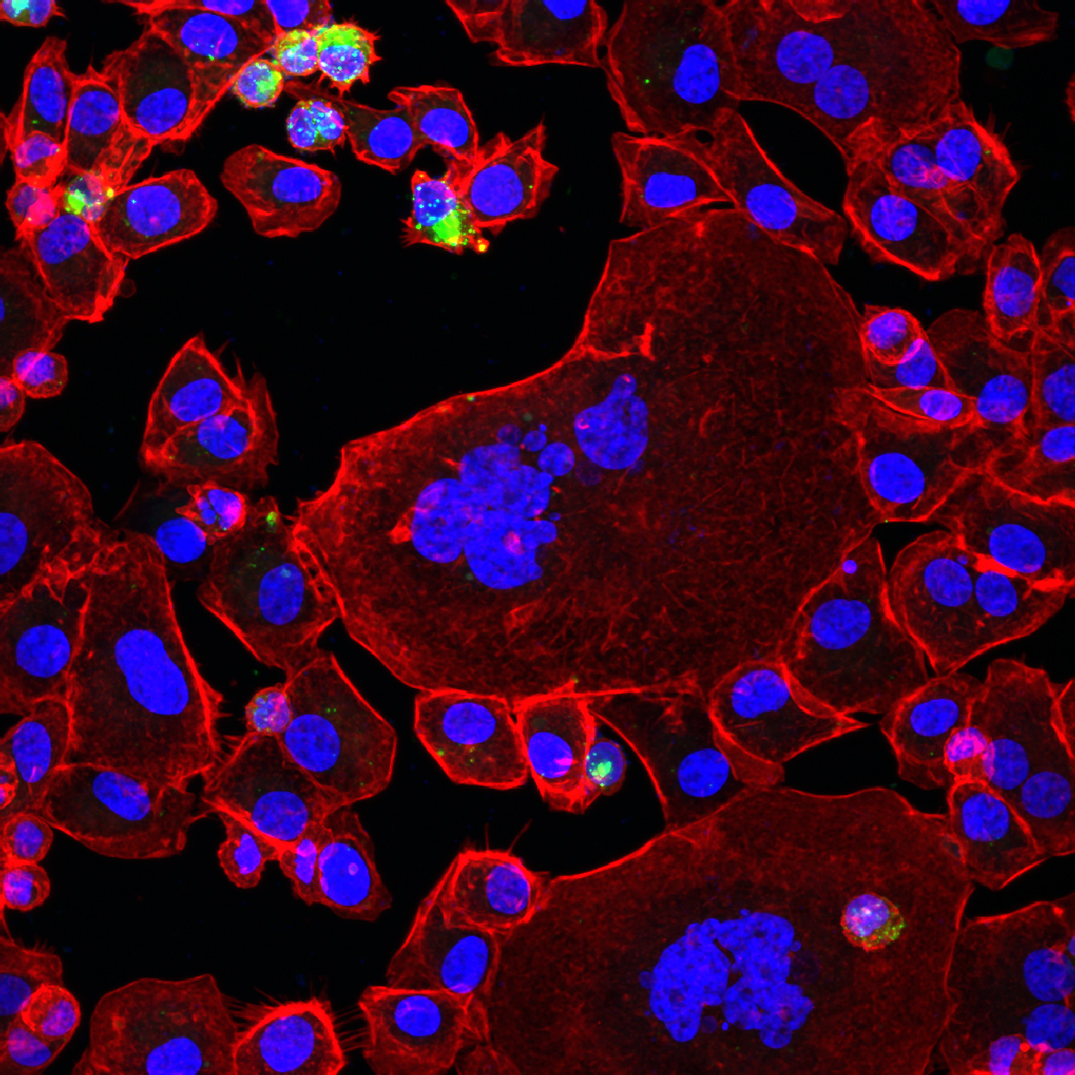

Supplement: Supplementary file 4 — Source data Fig. 3 [file 44321_2024_69_MOESM4_ESM.zip › Figure 3/IMAGES for Fig.3C/TOV PT-sen Ig ctrl.jpg]

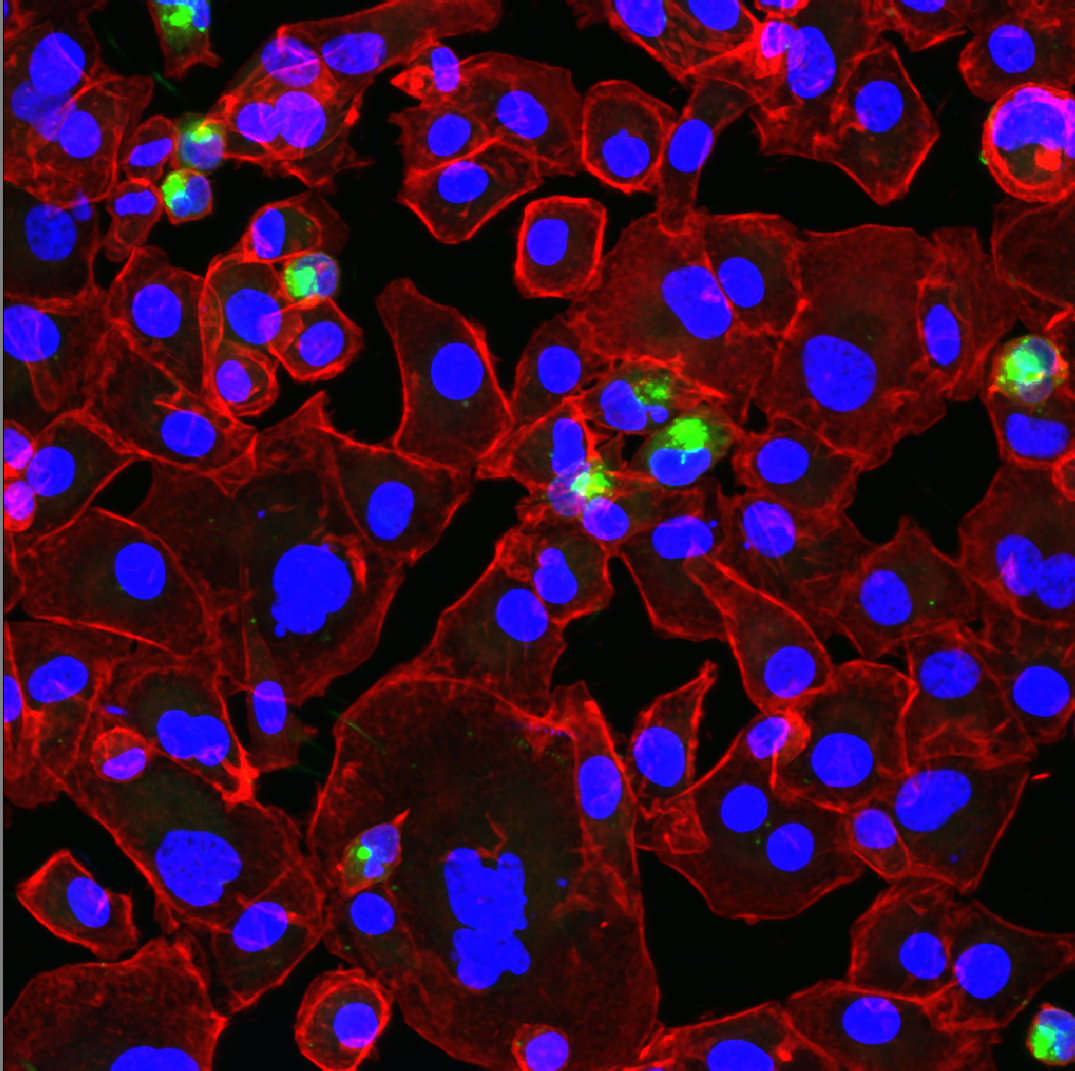

Supplement: Supplementary file 4 — Source data Fig. 3 [file 44321_2024_69_MOESM4_ESM.zip › Figure 3/IMAGES for Fig.3C/TOV PT-res Ig GoH3.jpg]

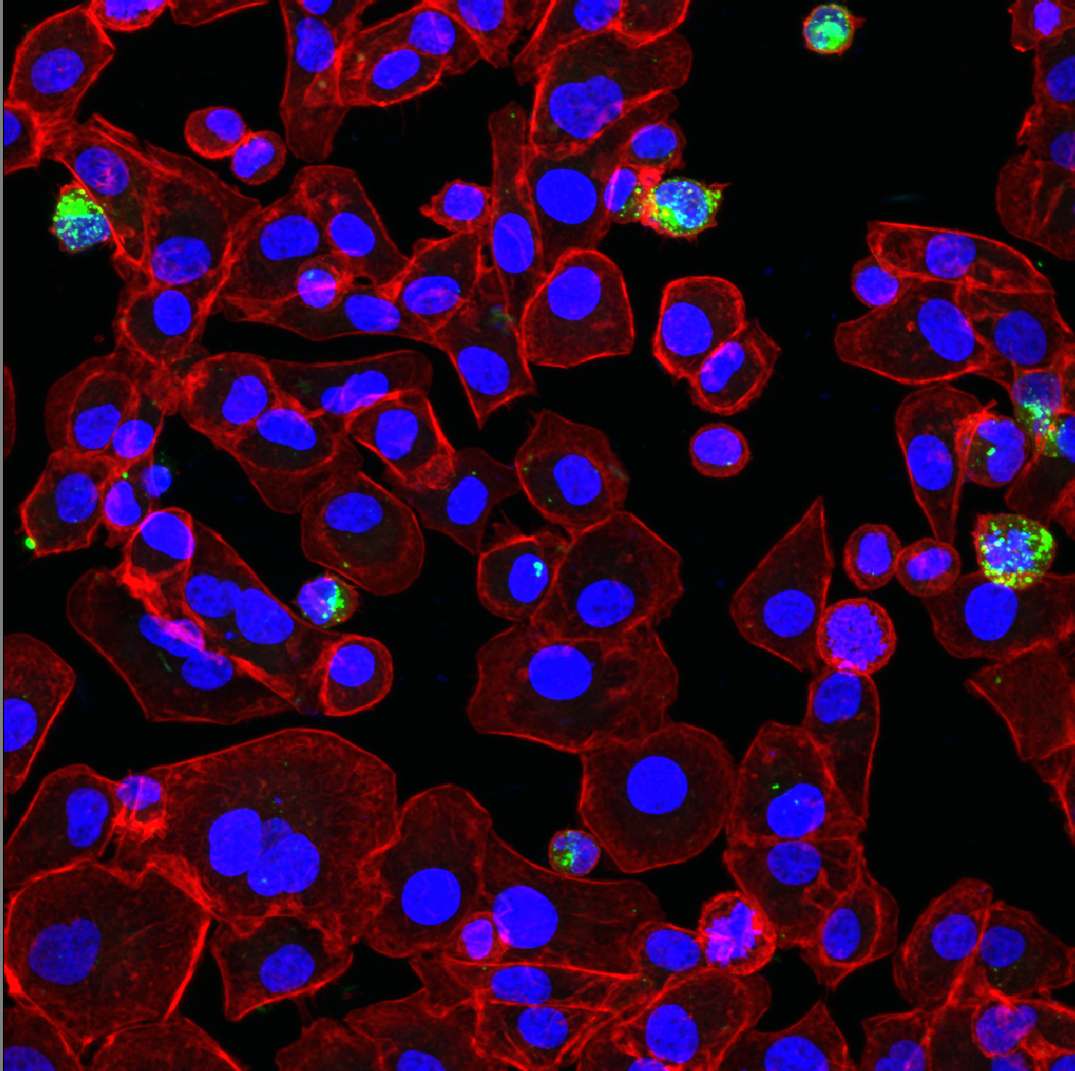

Supplement: Supplementary file 4 — Source data Fig. 3 [file 44321_2024_69_MOESM4_ESM.zip › Figure 3/IMAGES for Fig.3C/TOV PT-sen Ig GoH3.jpg]

**Figure 4**

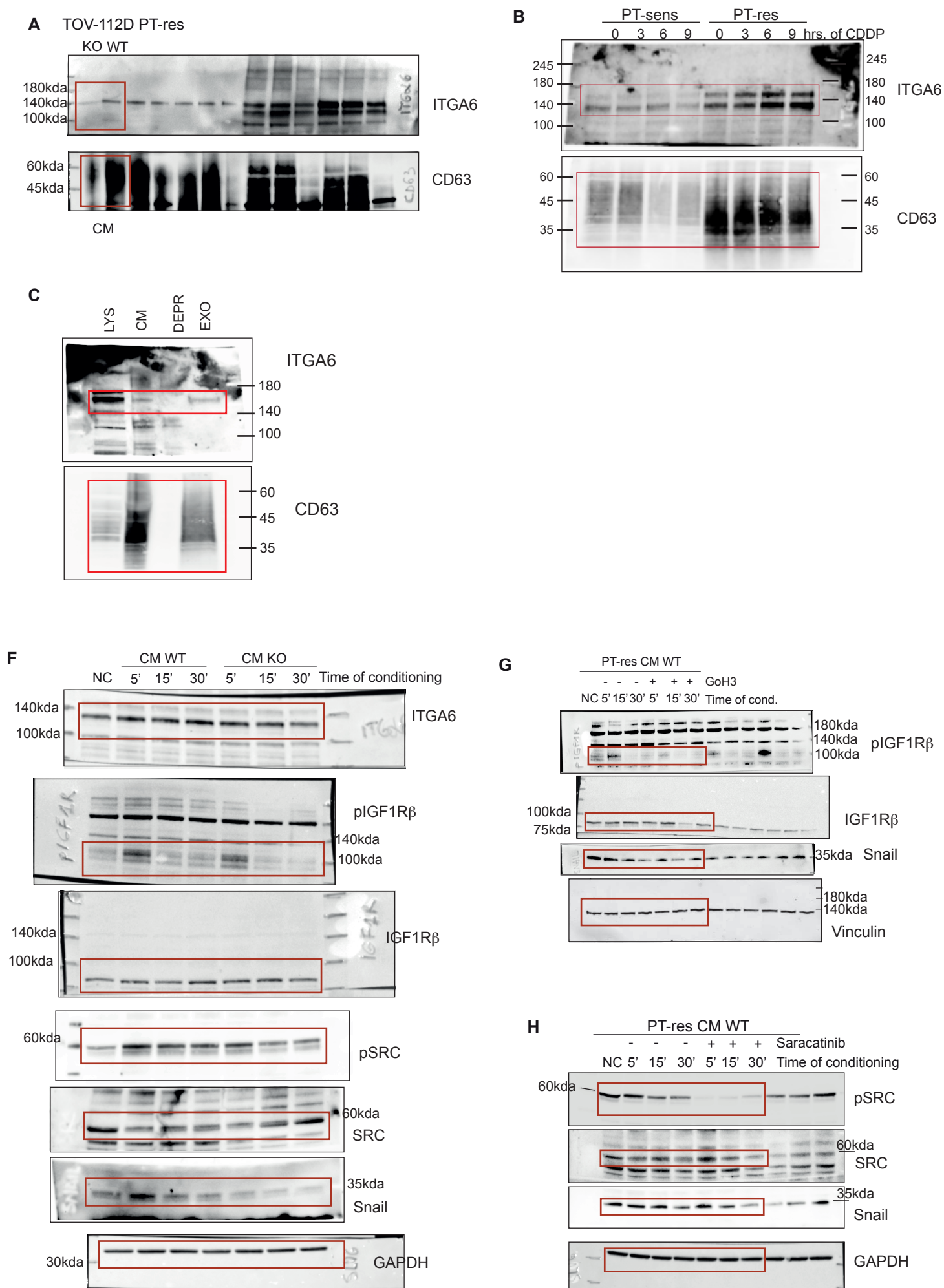

Supplement: Supplementary file 5 — Source data Fig. 4 [file 44321_2024_69_MOESM5_ESM.zip › Figure 4/Figure4 (1) source data.pdf]

Figure 4

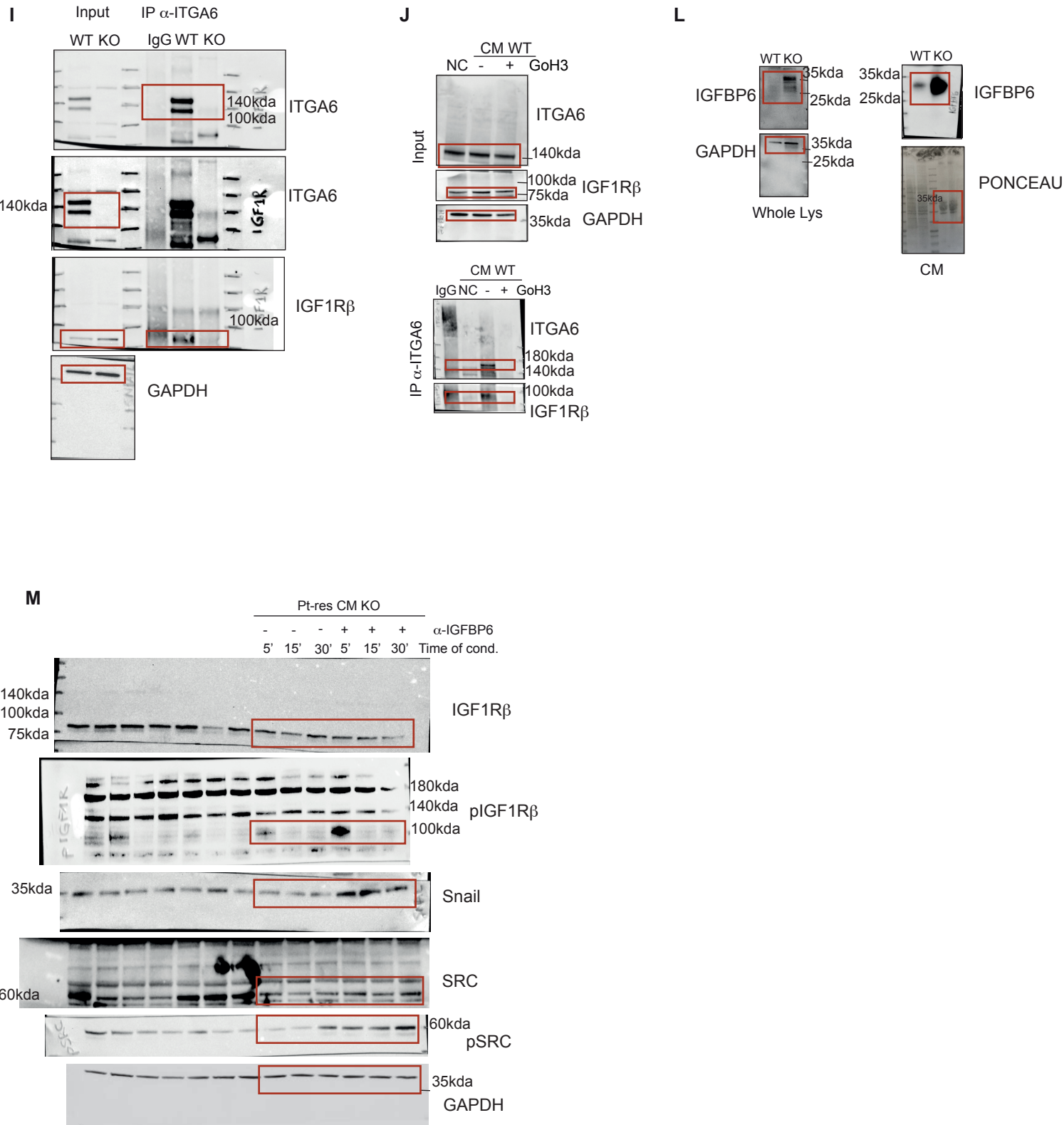

Supplement: Supplementary file 5 — Source data Fig. 4 [file 44321_2024_69_MOESM5_ESM.zip › Figure 4/Figure4 (2) source data.pdf]

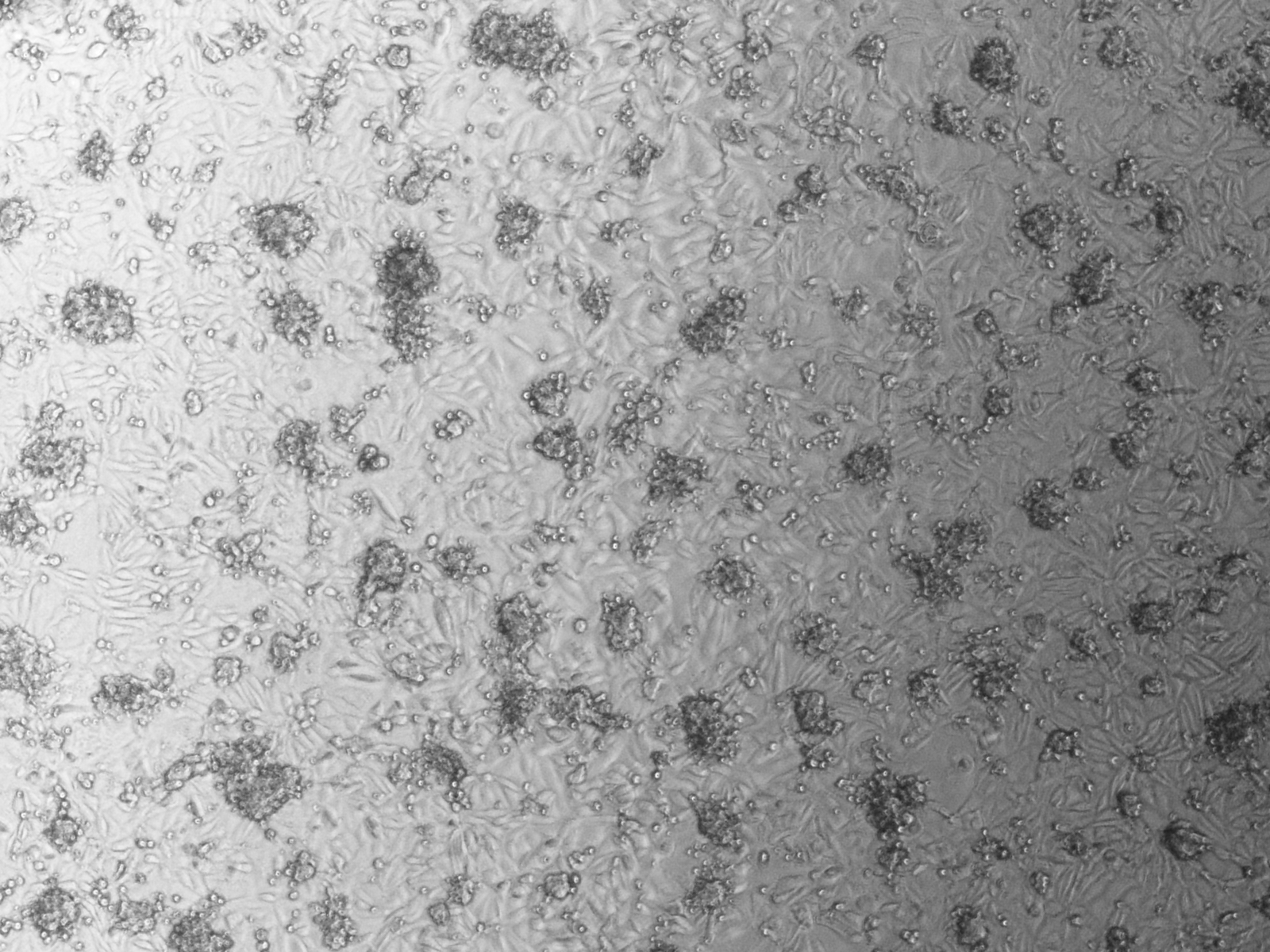

Supplement: Supplementary file 5 — Source data Fig. 4 [file 44321_2024_69_MOESM5_ESM.zip › Figure 4/IMAGES for Fig.4E/PT-sen CM WT.tif]

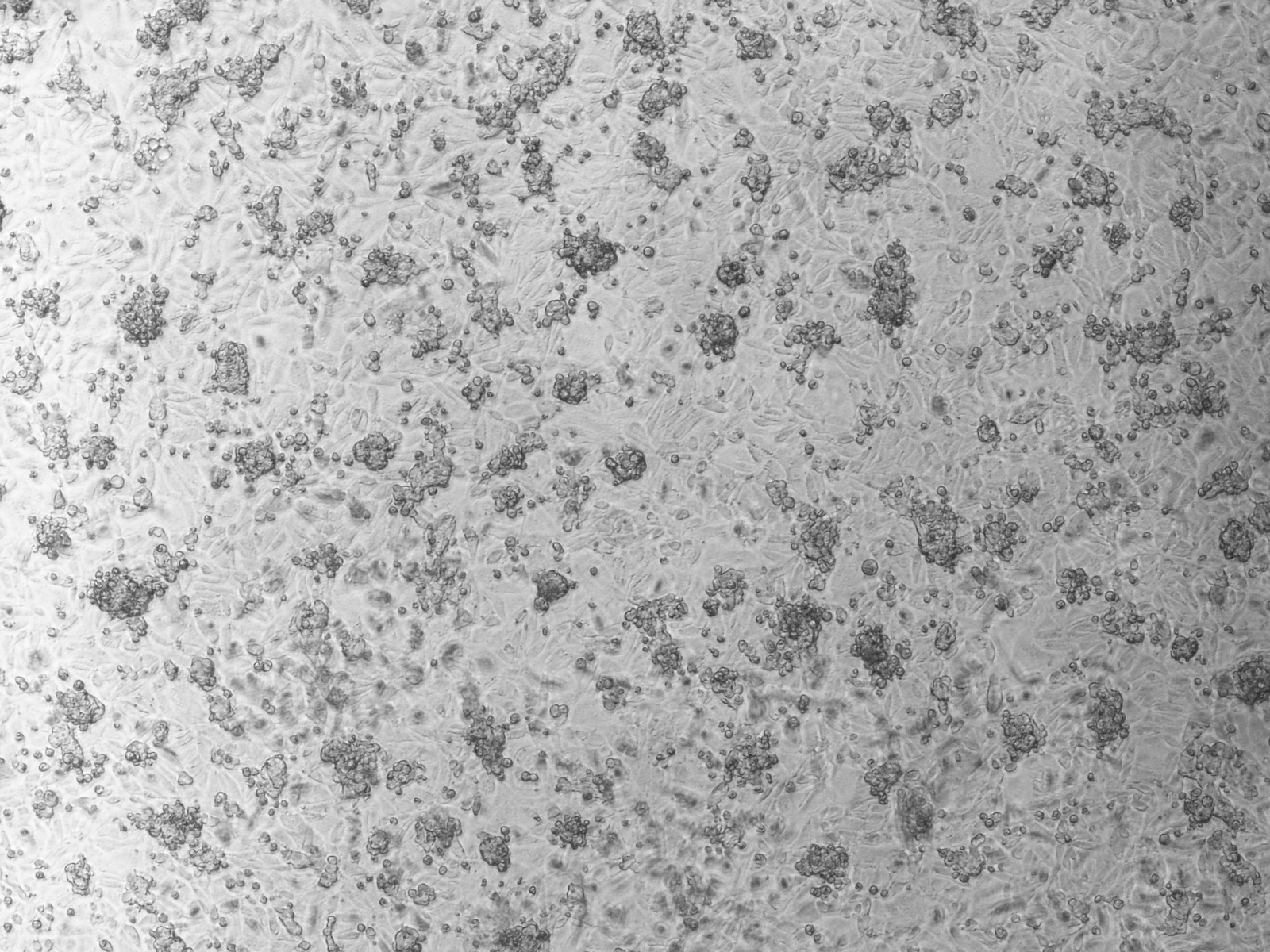

Supplement: Supplementary file 5 — Source data Fig. 4 [file 44321_2024_69_MOESM5_ESM.zip › Figure 4/IMAGES for Fig.4E/PT-sen not CM.tif]

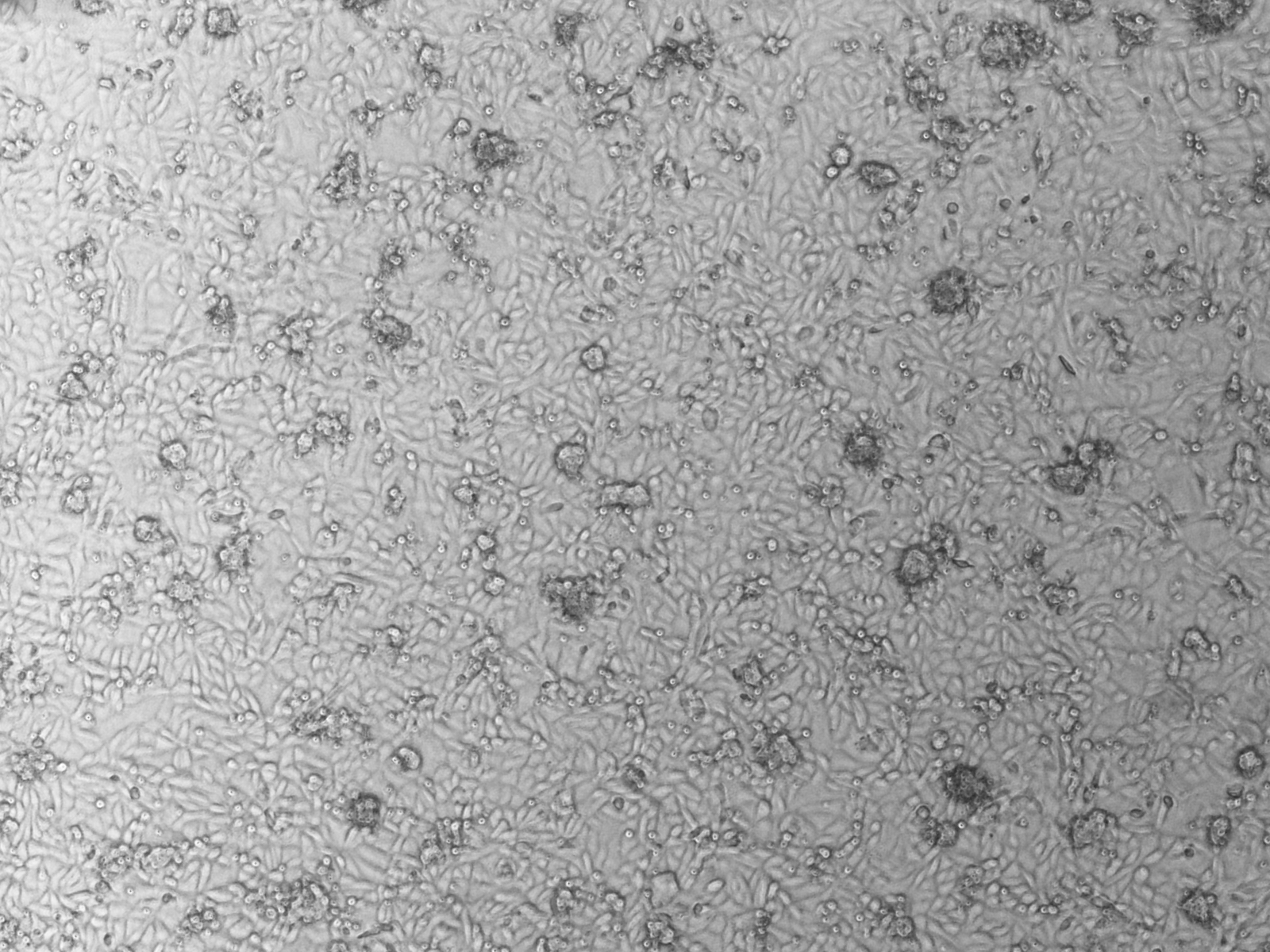

Supplement: Supplementary file 5 — Source data Fig. 4 [file 44321_2024_69_MOESM5_ESM.zip › Figure 4/IMAGES for Fig.4E/PT-sen CM ko (7).tif]

Figure 5

C

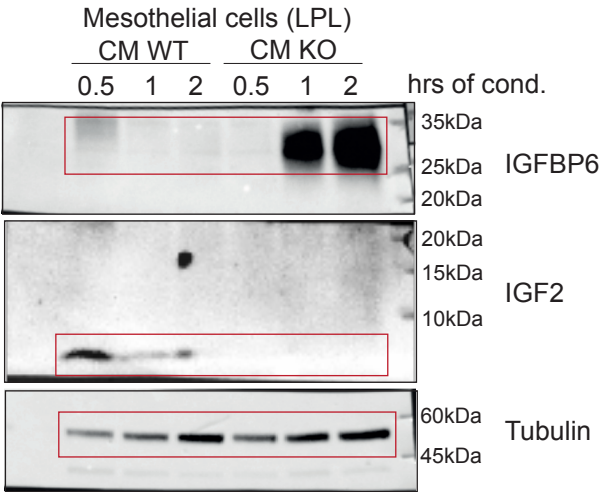

D

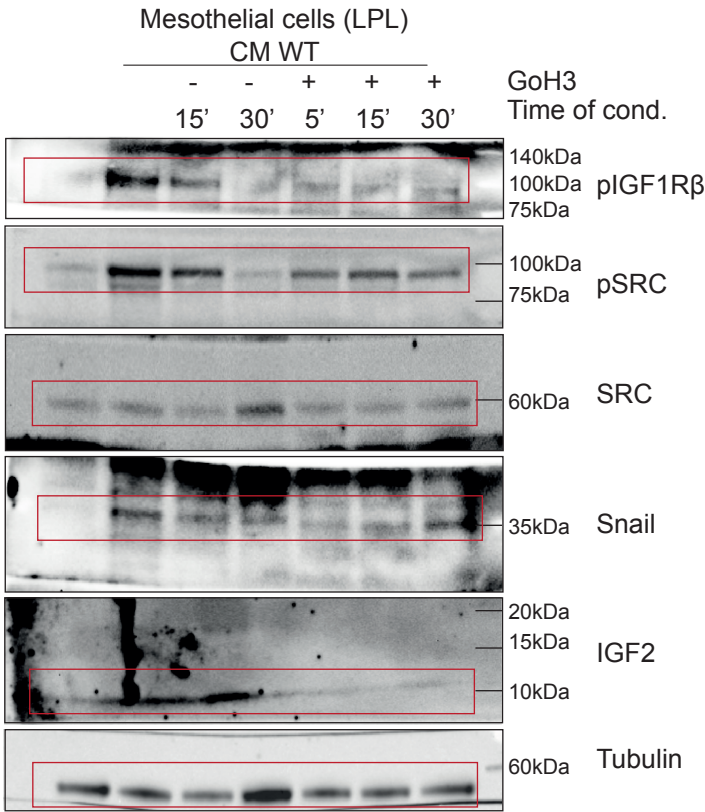

Supplement: Supplementary file 6 — Source data Fig. 5 [file 44321_2024_69_MOESM6_ESM.zip › Figure5 source data.pdf]

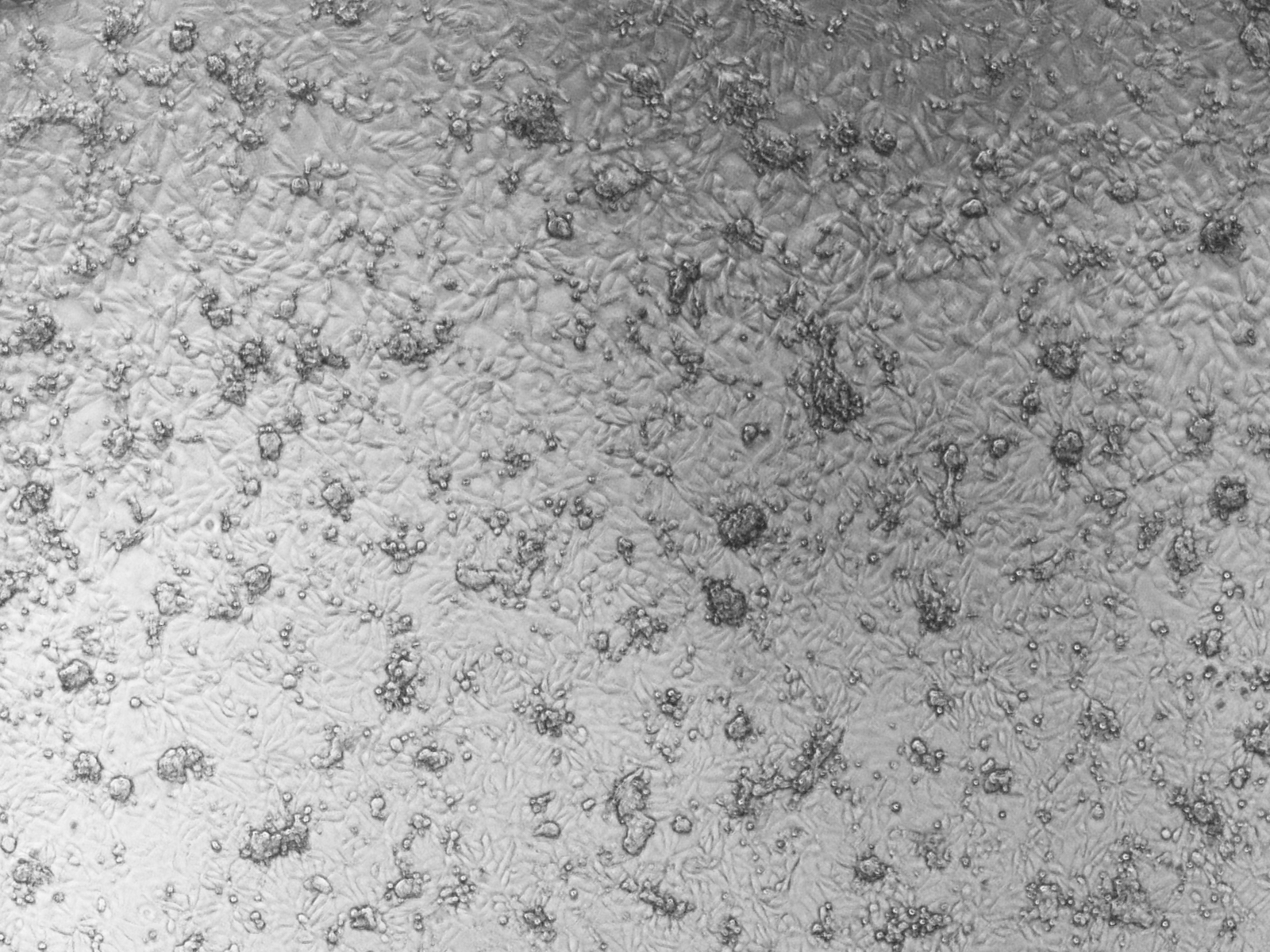

Supplement: Supplementary file 6 — Source data Fig. 5 [file 44321_2024_69_MOESM6_ESM.zip › IMAGES for Fig.5B/Mesothelial NOT CM.tif]

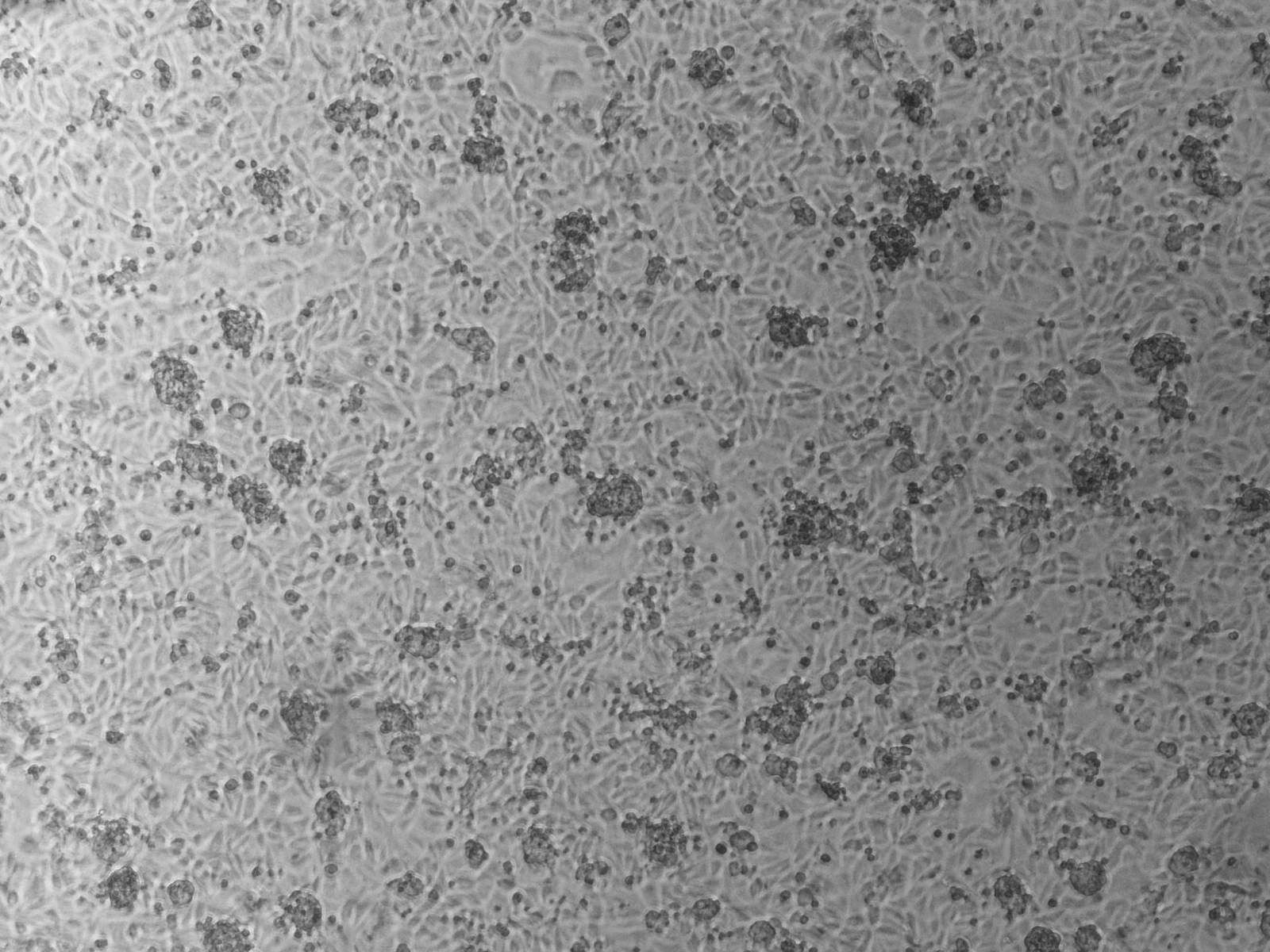

Supplement: Supplementary file 6 — Source data Fig. 5 [file 44321_2024_69_MOESM6_ESM.zip › IMAGES for Fig.5B/Mesothelial CM KO.tif]

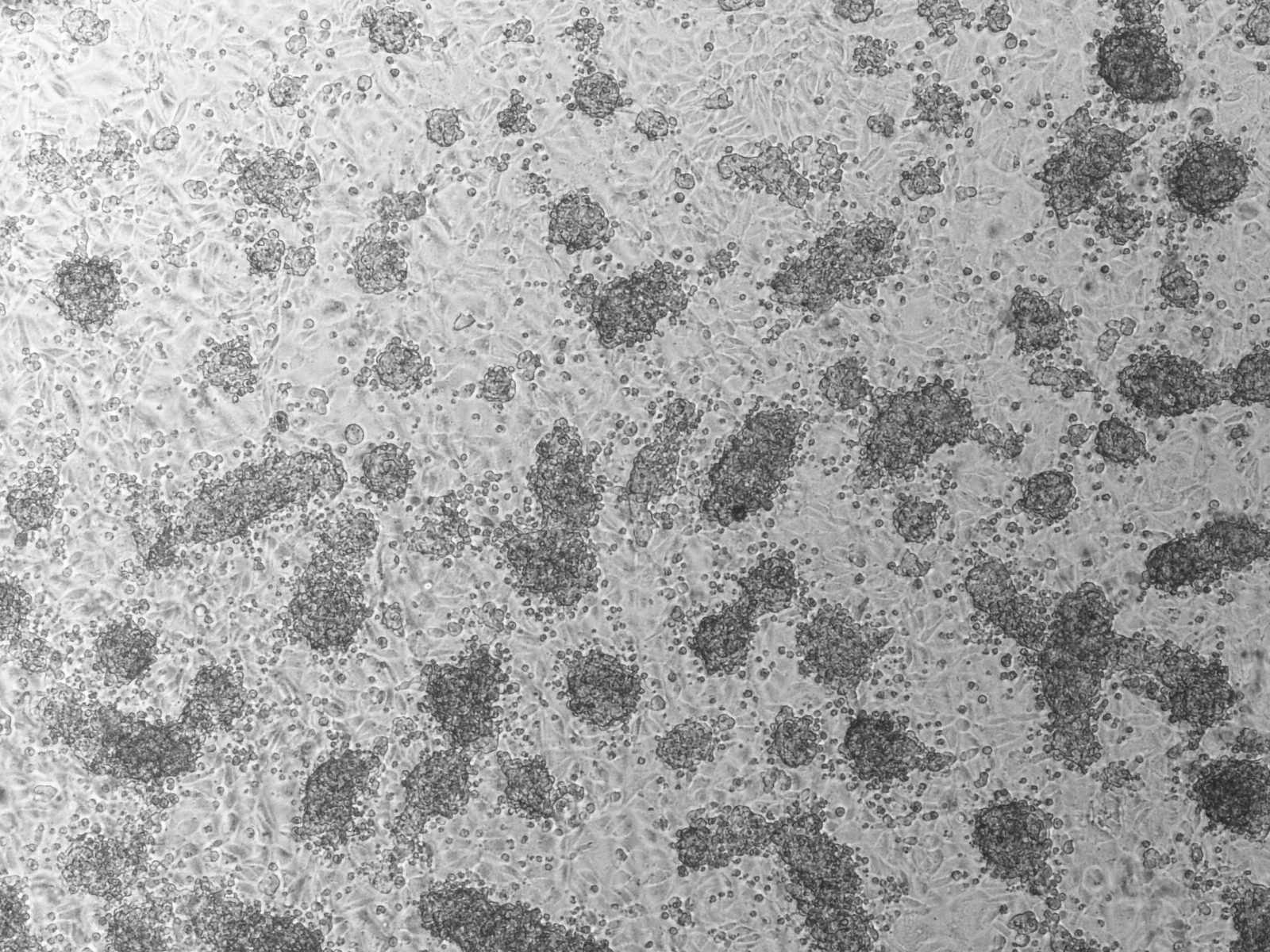

Supplement: Supplementary file 6 — Source data Fig. 5 [file 44321_2024_69_MOESM6_ESM.zip › IMAGES for Fig.5B/Mesothelial CM WT.tif]

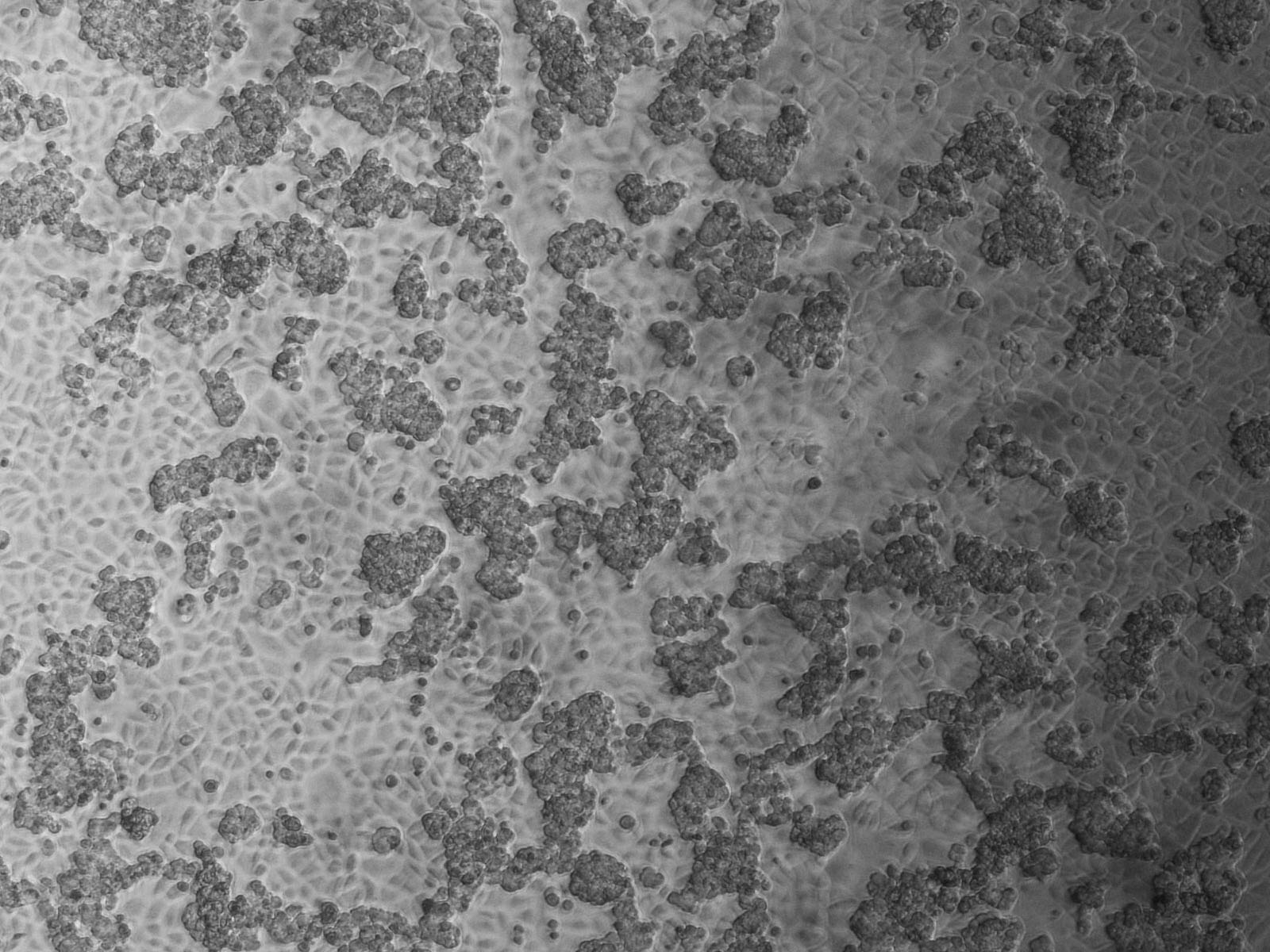

Supplement: Supplementary file 6 — Source data Fig. 5 [file 44321_2024_69_MOESM6_ESM.zip › IMAGES for Fig.5G/Mesoth. CM Asc.OV221+IgG.jpg]

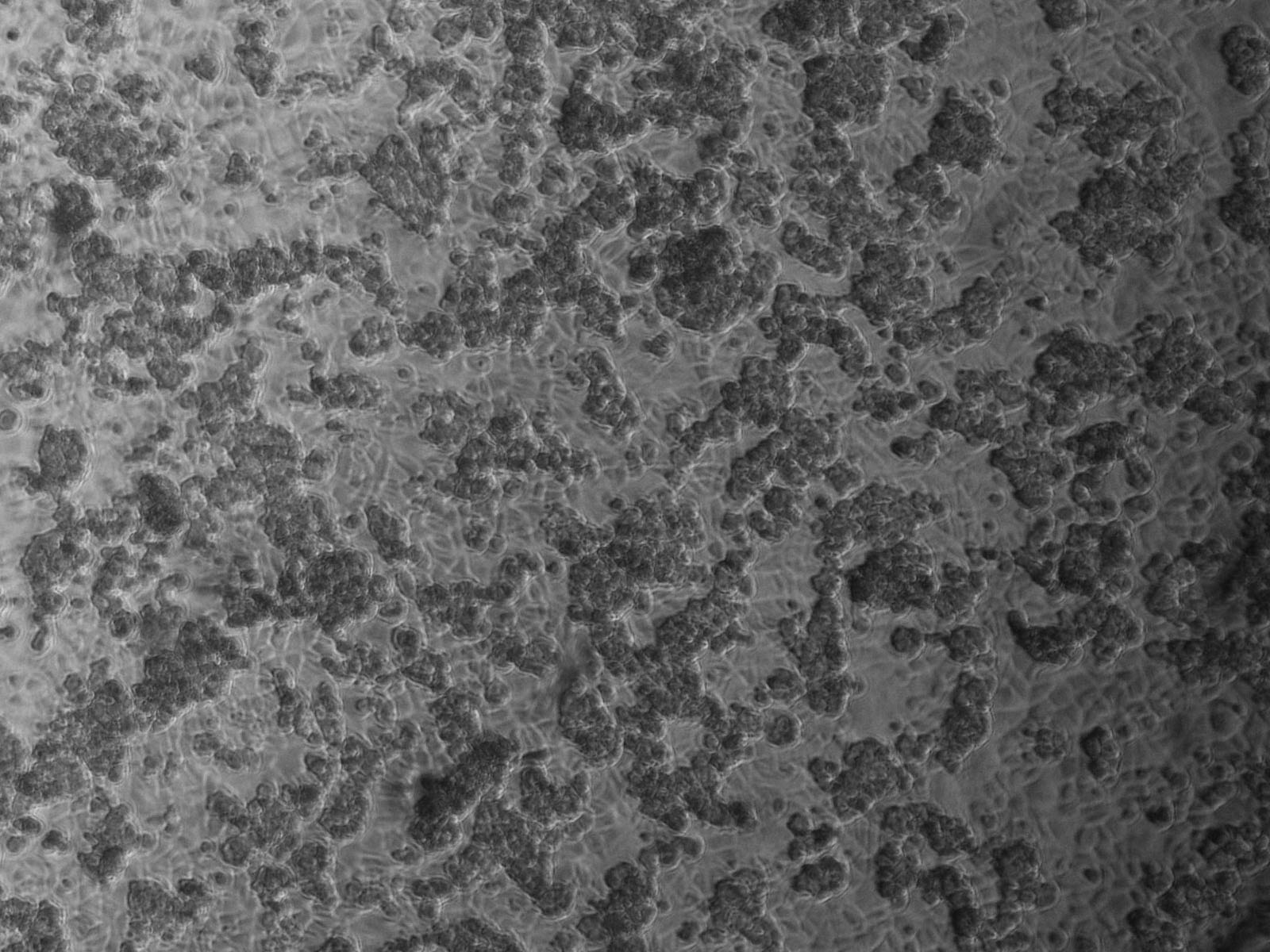

Supplement: Supplementary file 6 — Source data Fig. 5 [file 44321_2024_69_MOESM6_ESM.zip › IMAGES for Fig.5G/Mesoth. CM Asc.OV239+IgG.jpg]

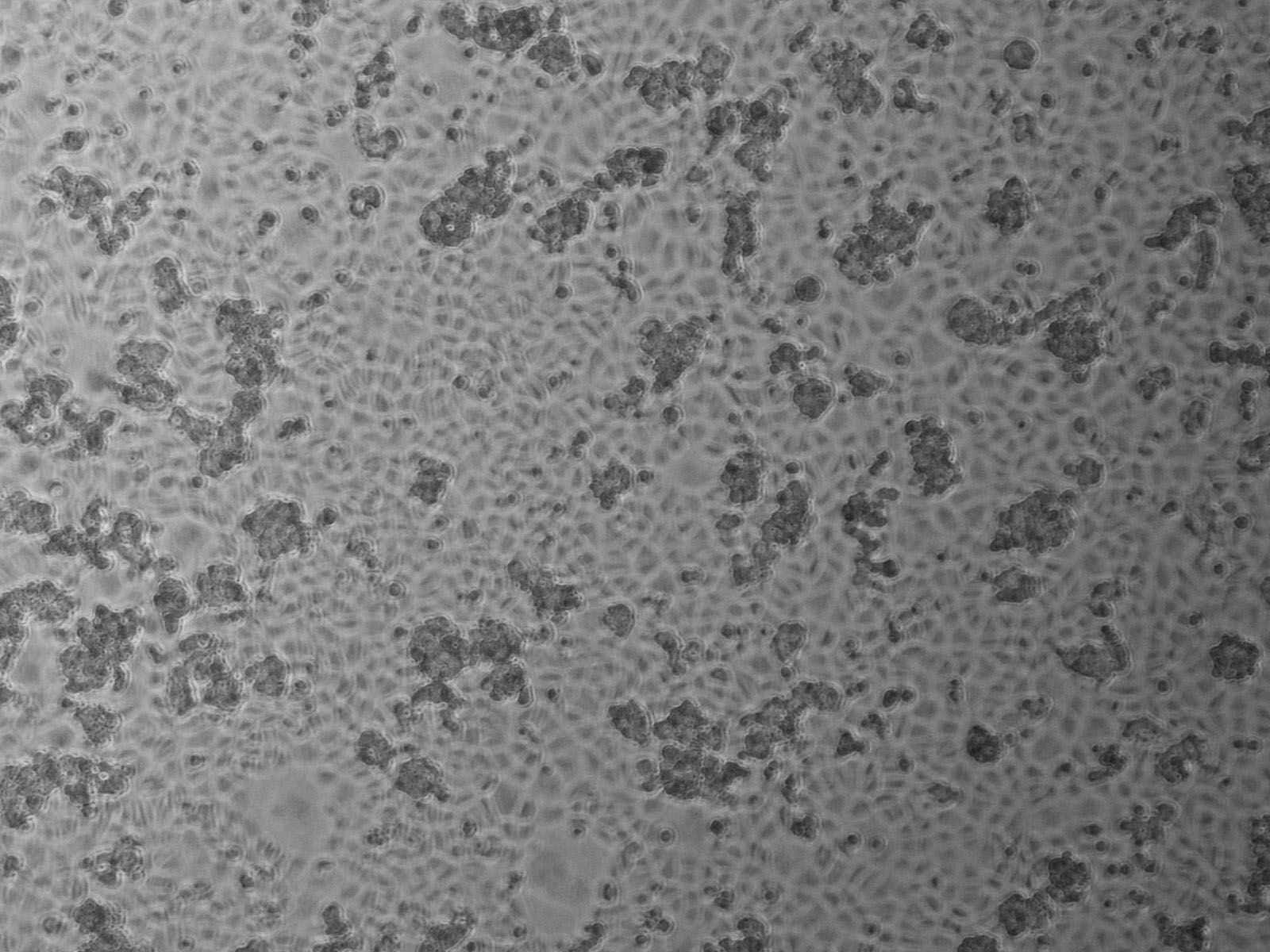

Supplement: Supplementary file 6 — Source data Fig. 5 [file 44321_2024_69_MOESM6_ESM.zip › IMAGES for Fig.5G/Mesoth. CM Asc.OV221+GoH3.jpg]

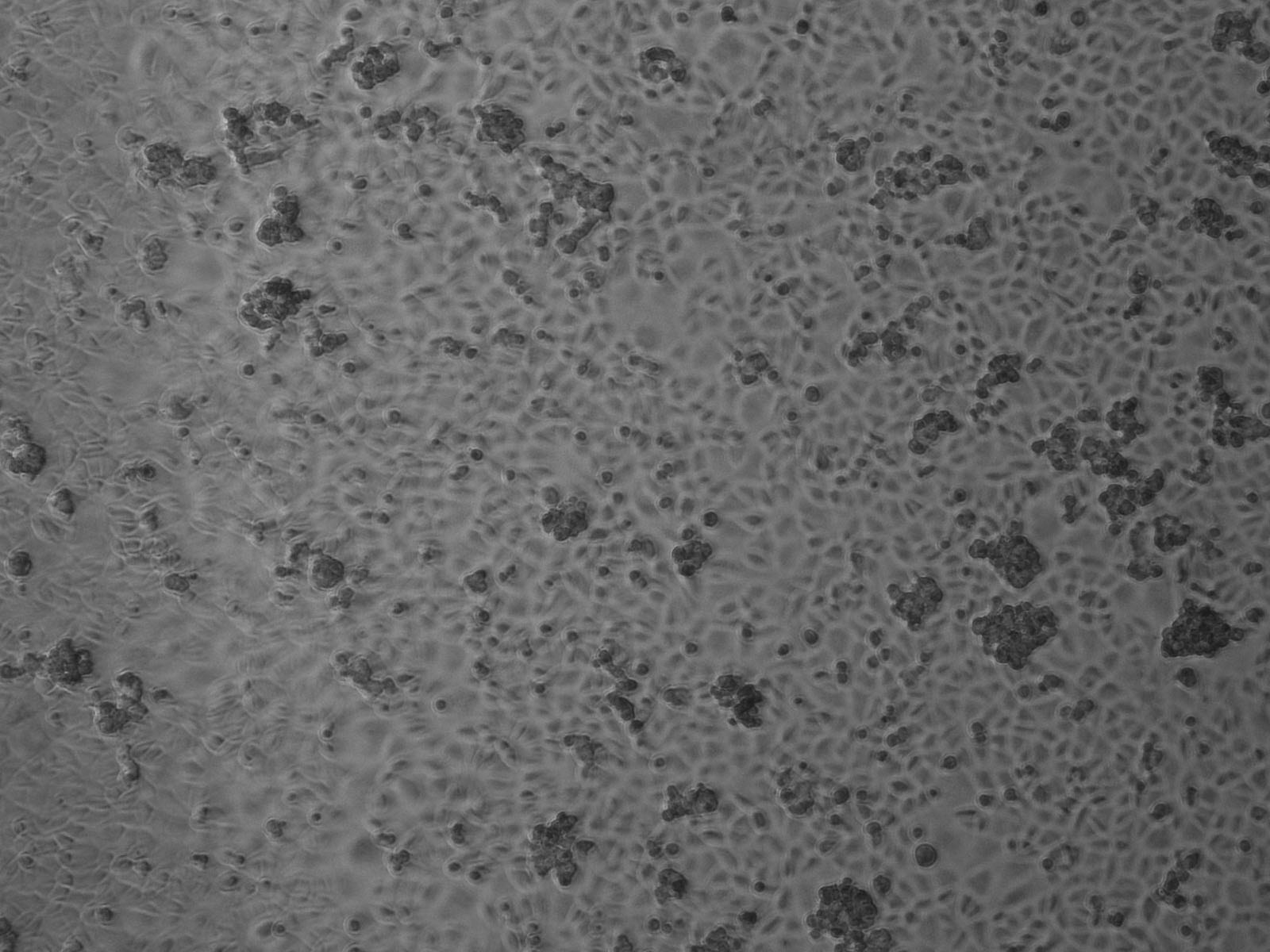

Supplement: Supplementary file 6 — Source data Fig. 5 [file 44321_2024_69_MOESM6_ESM.zip › IMAGES for Fig.5G/Mesoth. CM Asc.OV239+GoH3.jpg]

Figure 6

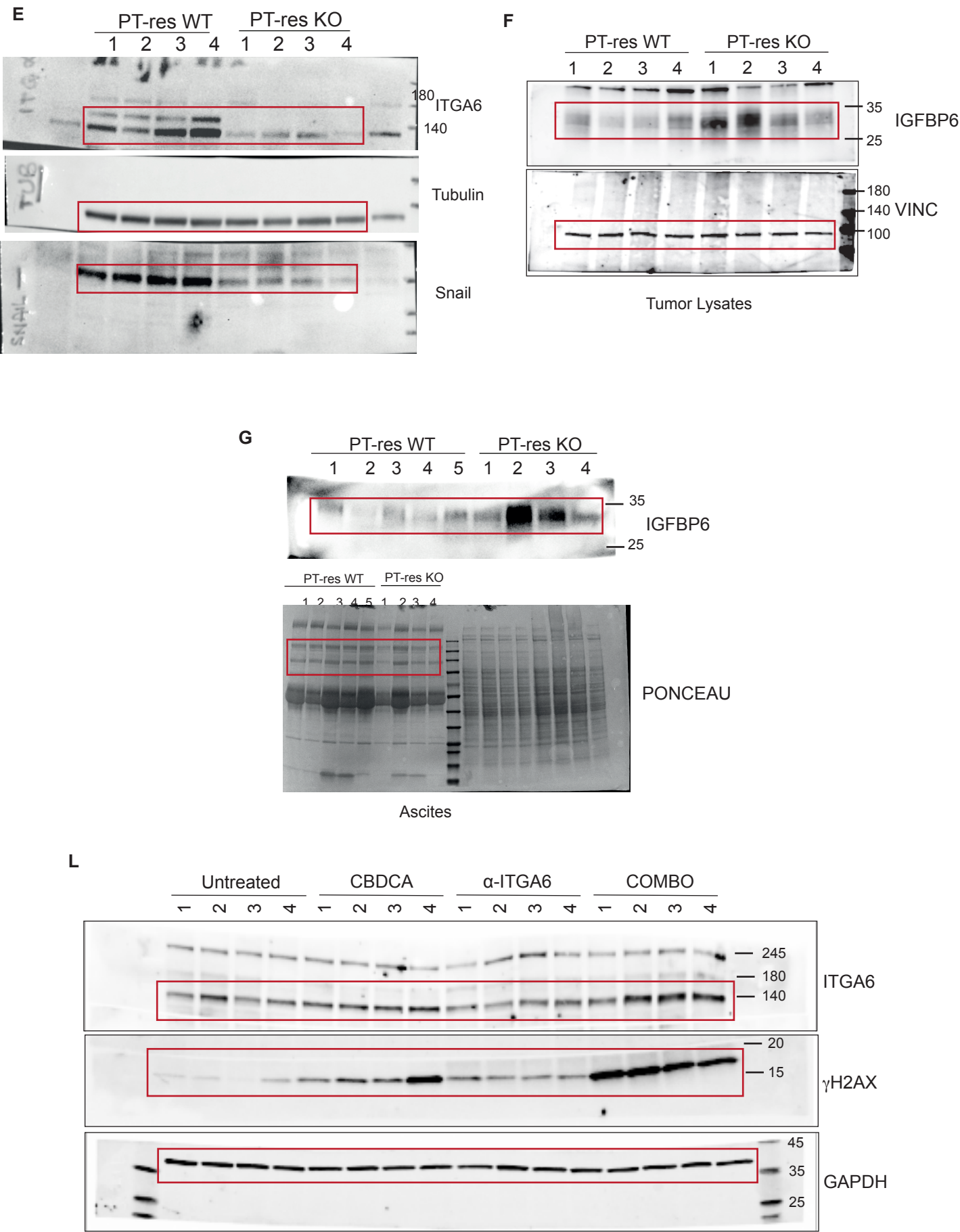

Supplement: Supplementary file 7 — Source data Fig. 6 [file 44321_2024_69_MOESM7_ESM.zip › Figure 6/Figure6 source data.pdf]

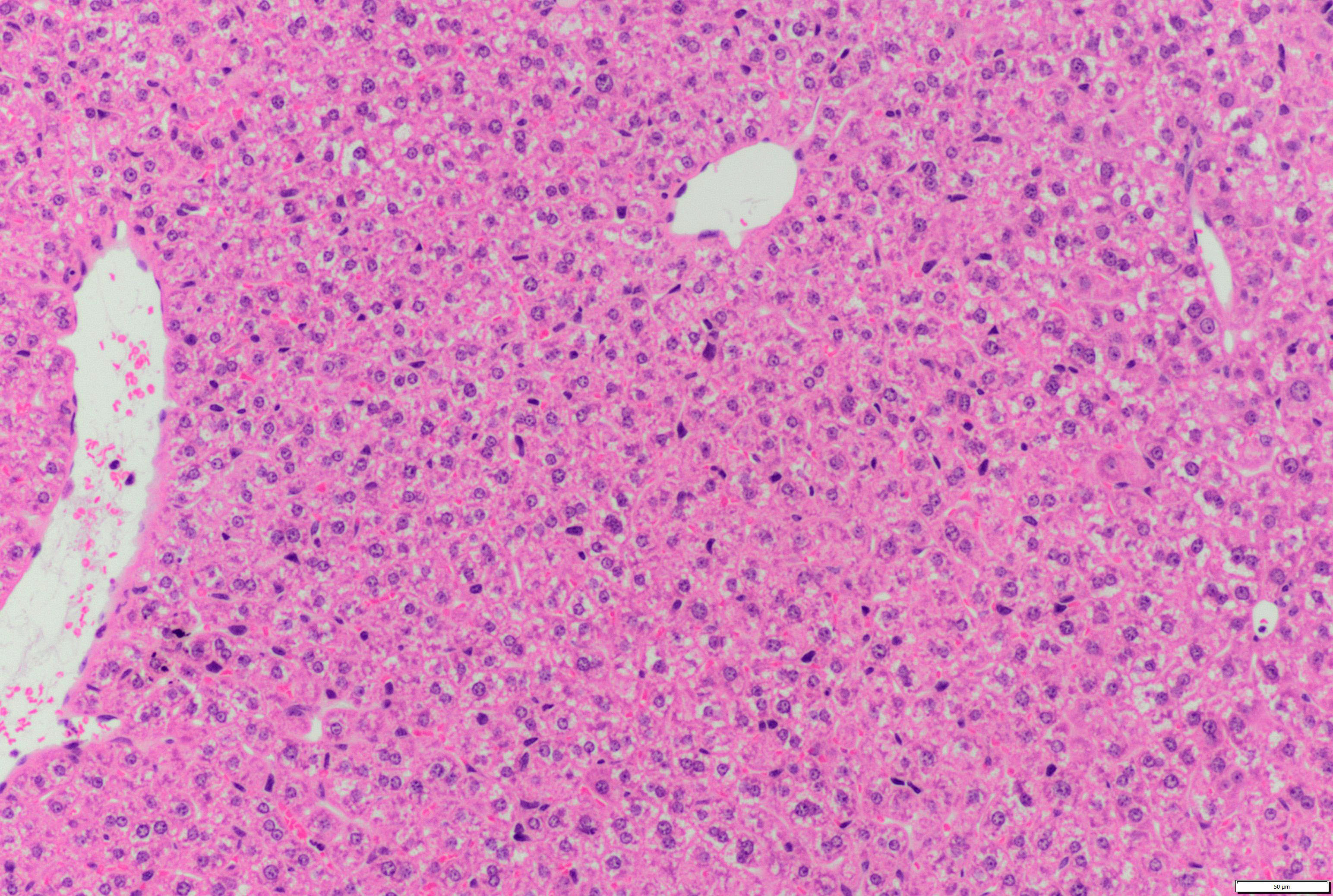

Supplement: Supplementary file 7 — Source data Fig. 6 [file 44321_2024_69_MOESM7_ESM.zip › Figure 6/IMAGES for Fig.6D/H&E Liver KO CBDCA 10x.jpg]

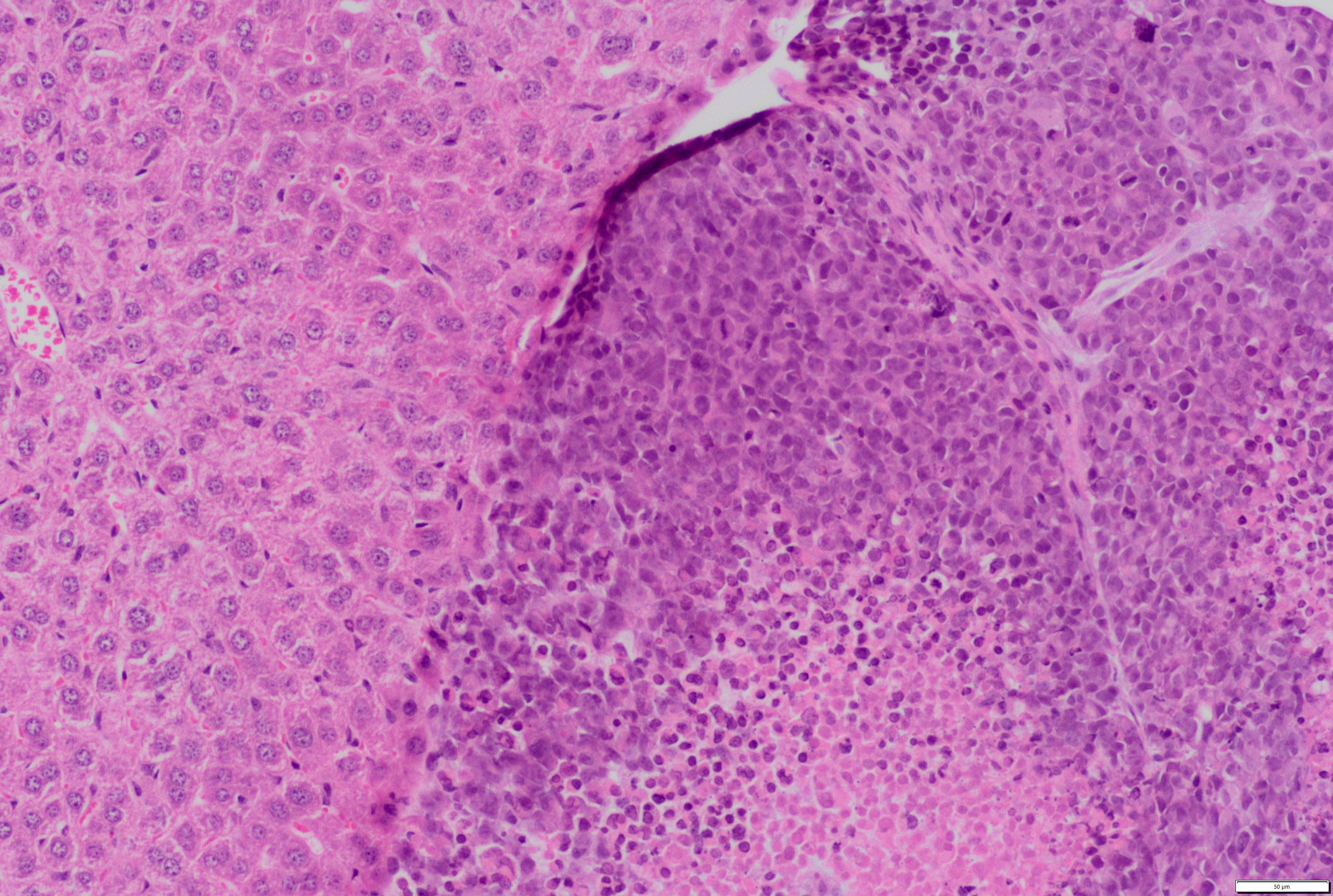

Supplement: Supplementary file 7 — Source data Fig. 6 [file 44321_2024_69_MOESM7_ESM.zip › Figure 6/IMAGES for Fig.6D/H&E Liver WT UNT 10x.jpg]

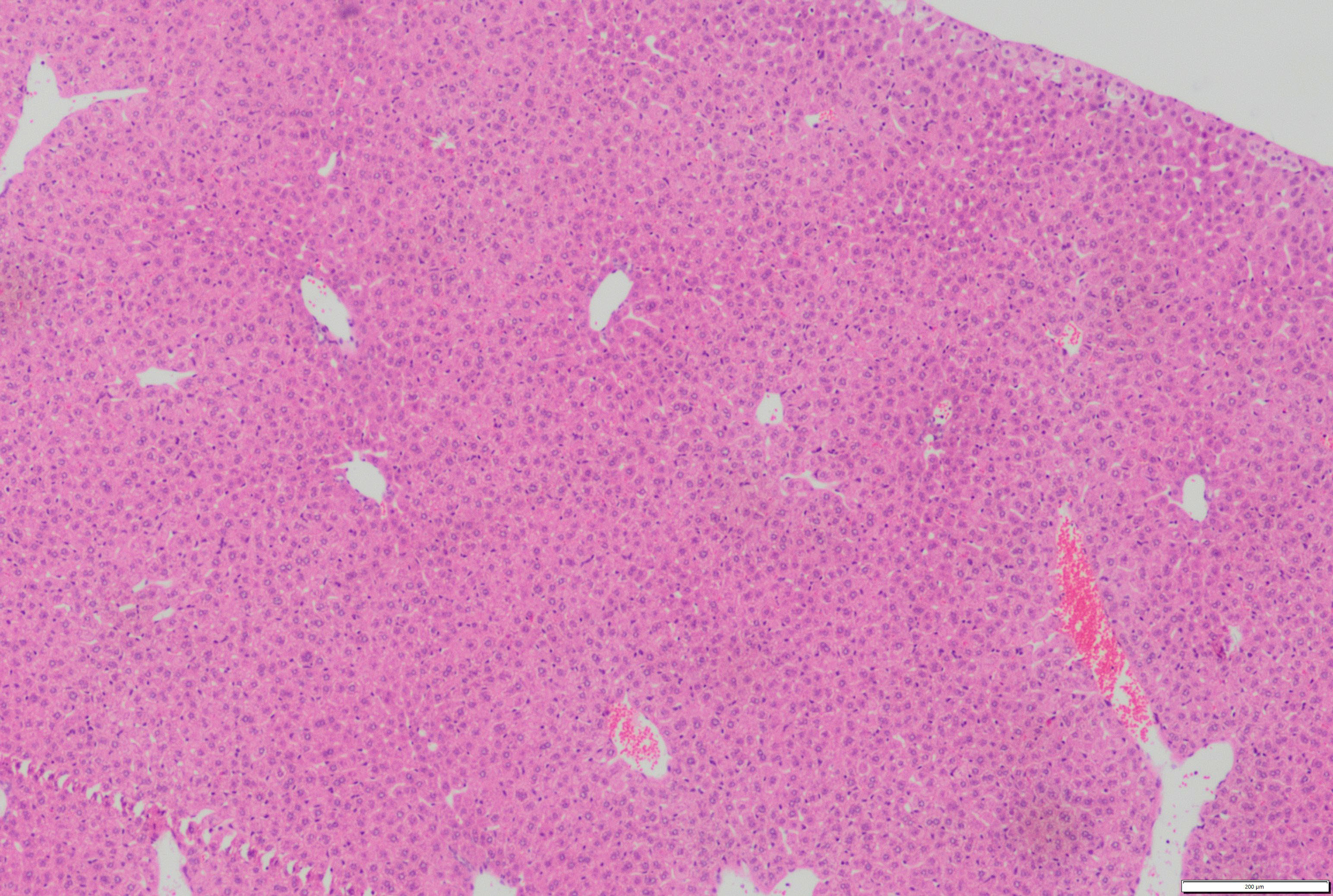

Supplement: Supplementary file 7 — Source data Fig. 6 [file 44321_2024_69_MOESM7_ESM.zip › Figure 6/IMAGES for Fig.6D/H&E Liver KO UNT 5x.jpg]

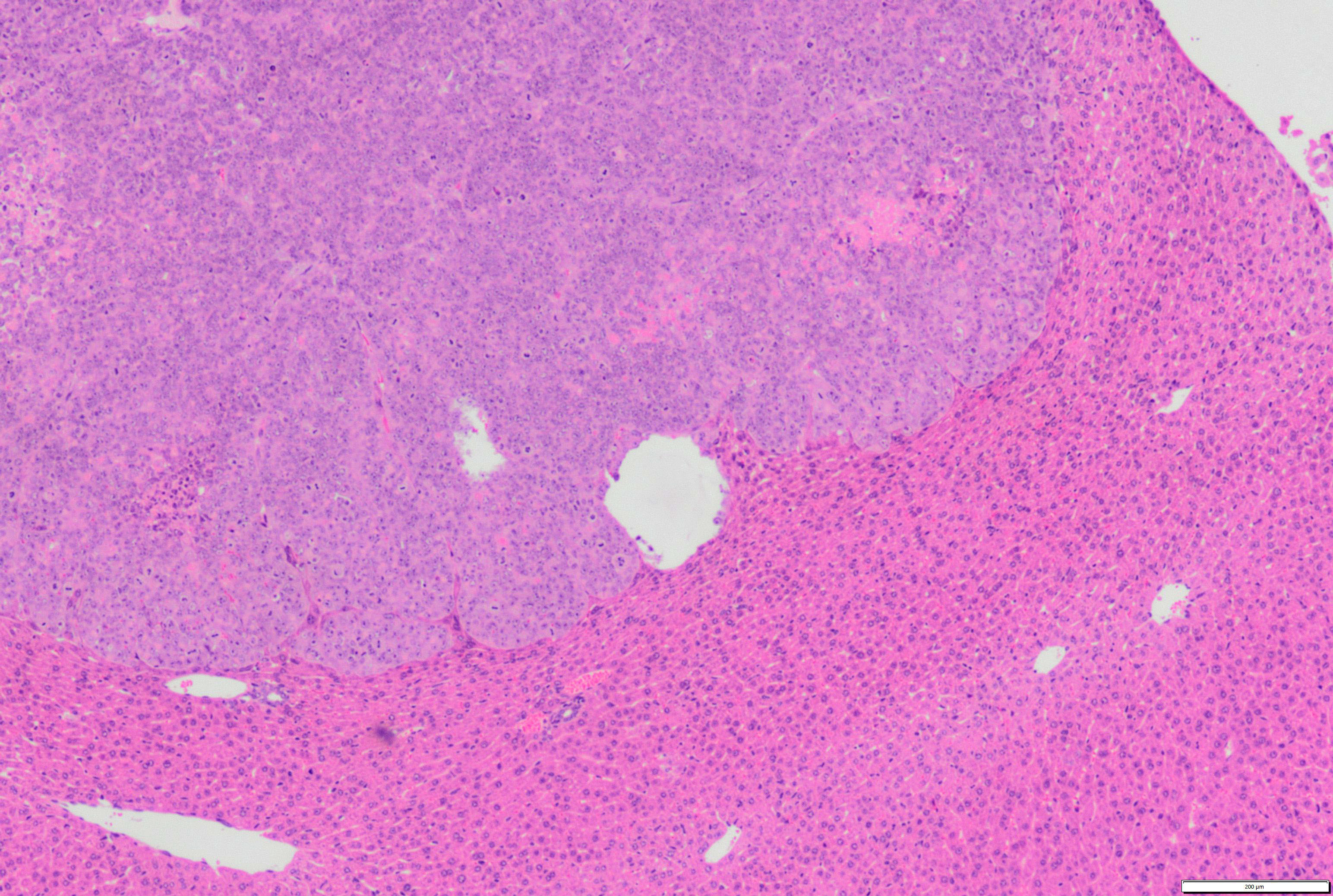

Supplement: Supplementary file 7 — Source data Fig. 6 [file 44321_2024_69_MOESM7_ESM.zip › Figure 6/IMAGES for Fig.6D/H&E Liver WT CBDCA 5x.jpg]

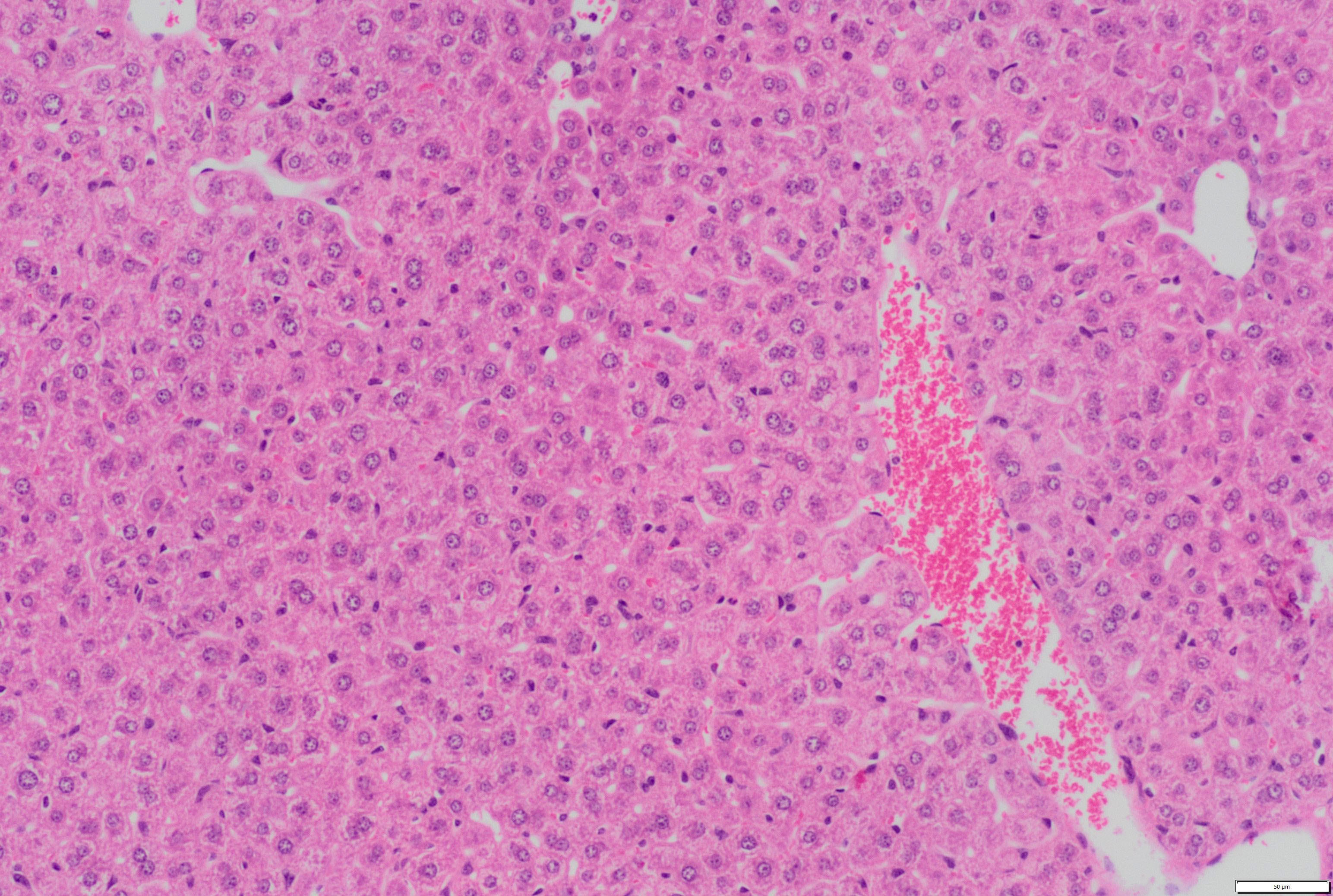

Supplement: Supplementary file 7 — Source data Fig. 6 [file 44321_2024_69_MOESM7_ESM.zip › Figure 6/IMAGES for Fig.6D/H&E Liver KO UNT 10x.jpg]

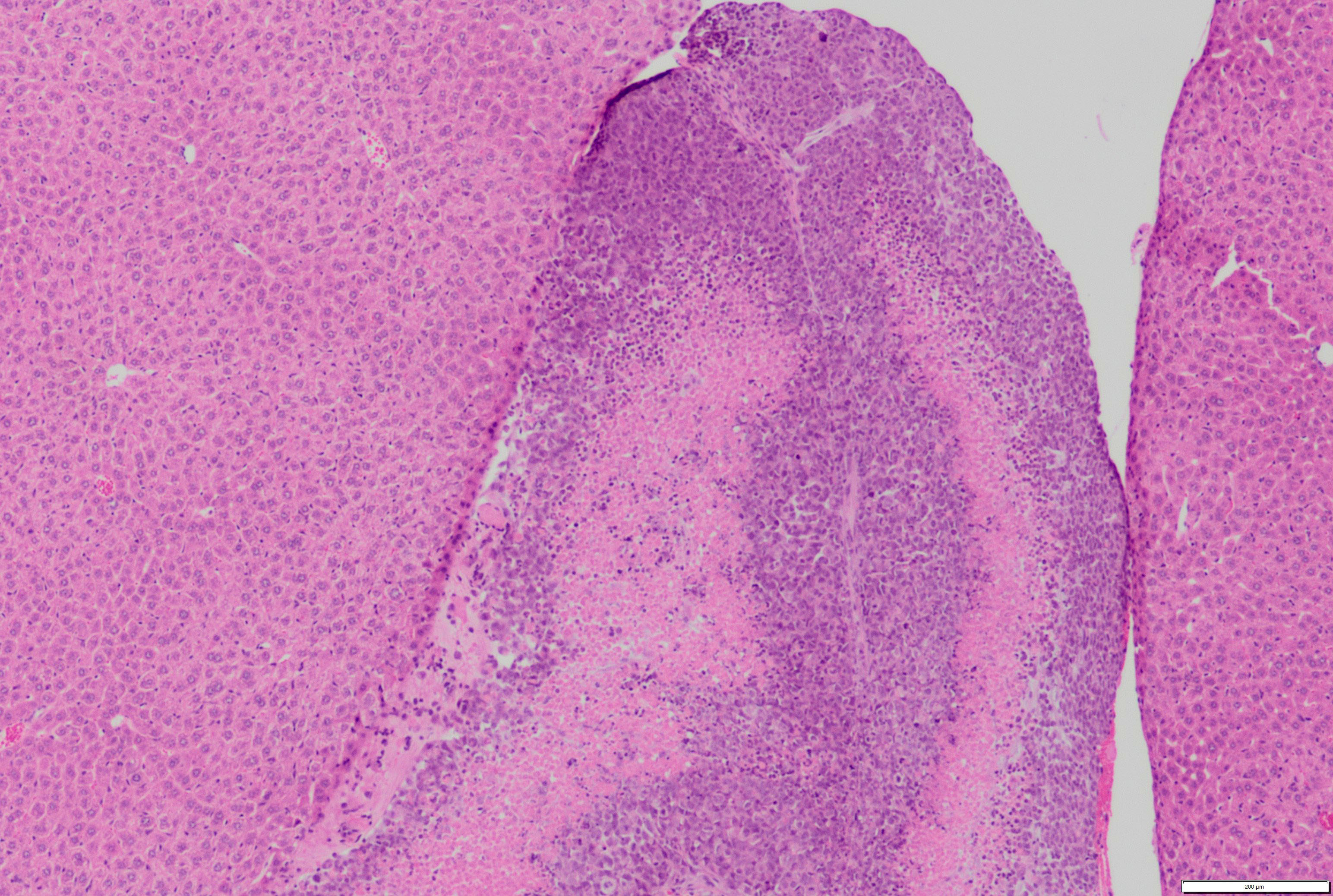

Supplement: Supplementary file 7 — Source data Fig. 6 [file 44321_2024_69_MOESM7_ESM.zip › Figure 6/IMAGES for Fig.6D/H&E Liver WT UNT 5x.jpg]

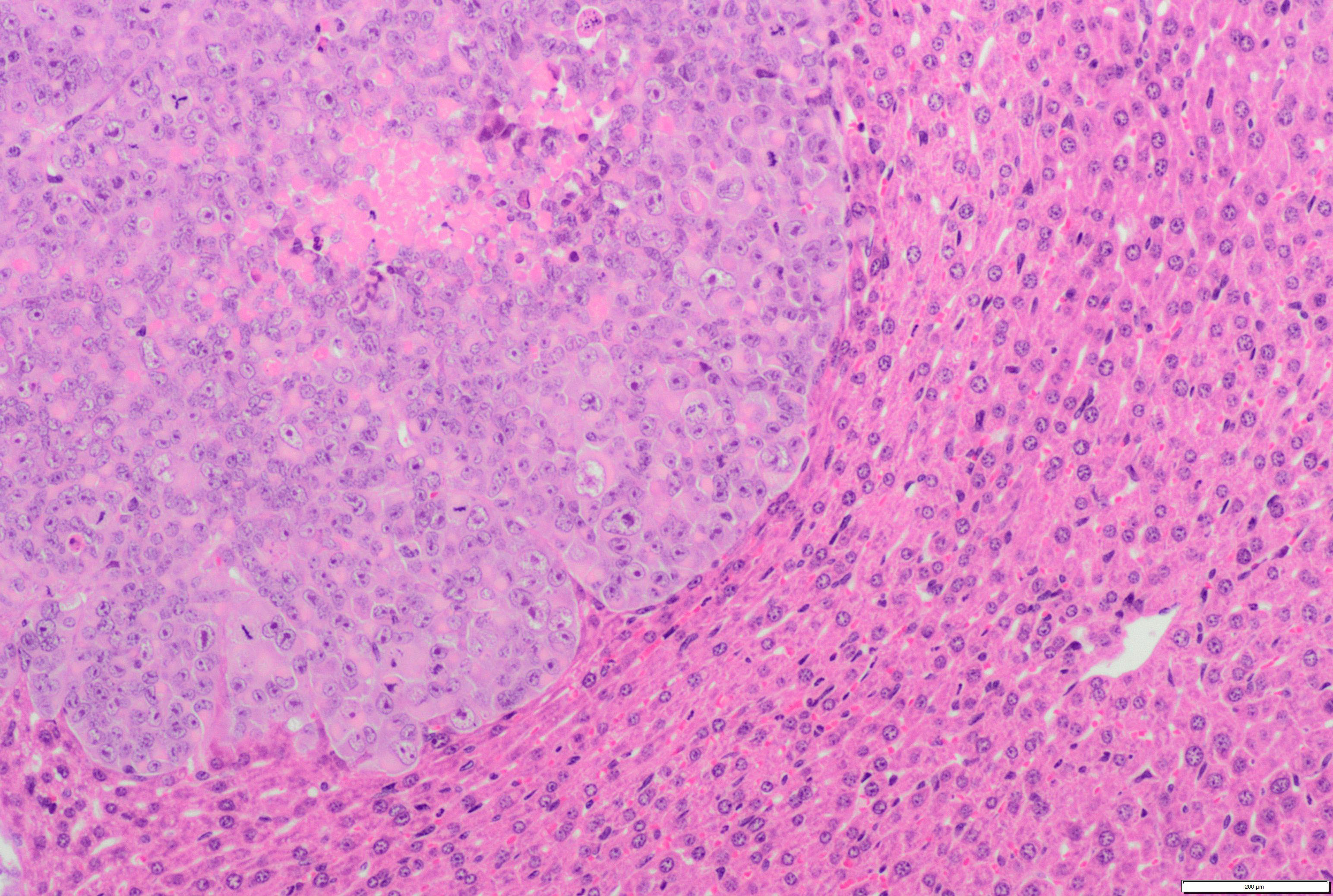

Supplement: Supplementary file 7 — Source data Fig. 6 [file 44321_2024_69_MOESM7_ESM.zip › Figure 6/IMAGES for Fig.6D/H&E Liver WT CBDCA 10x.jpg]

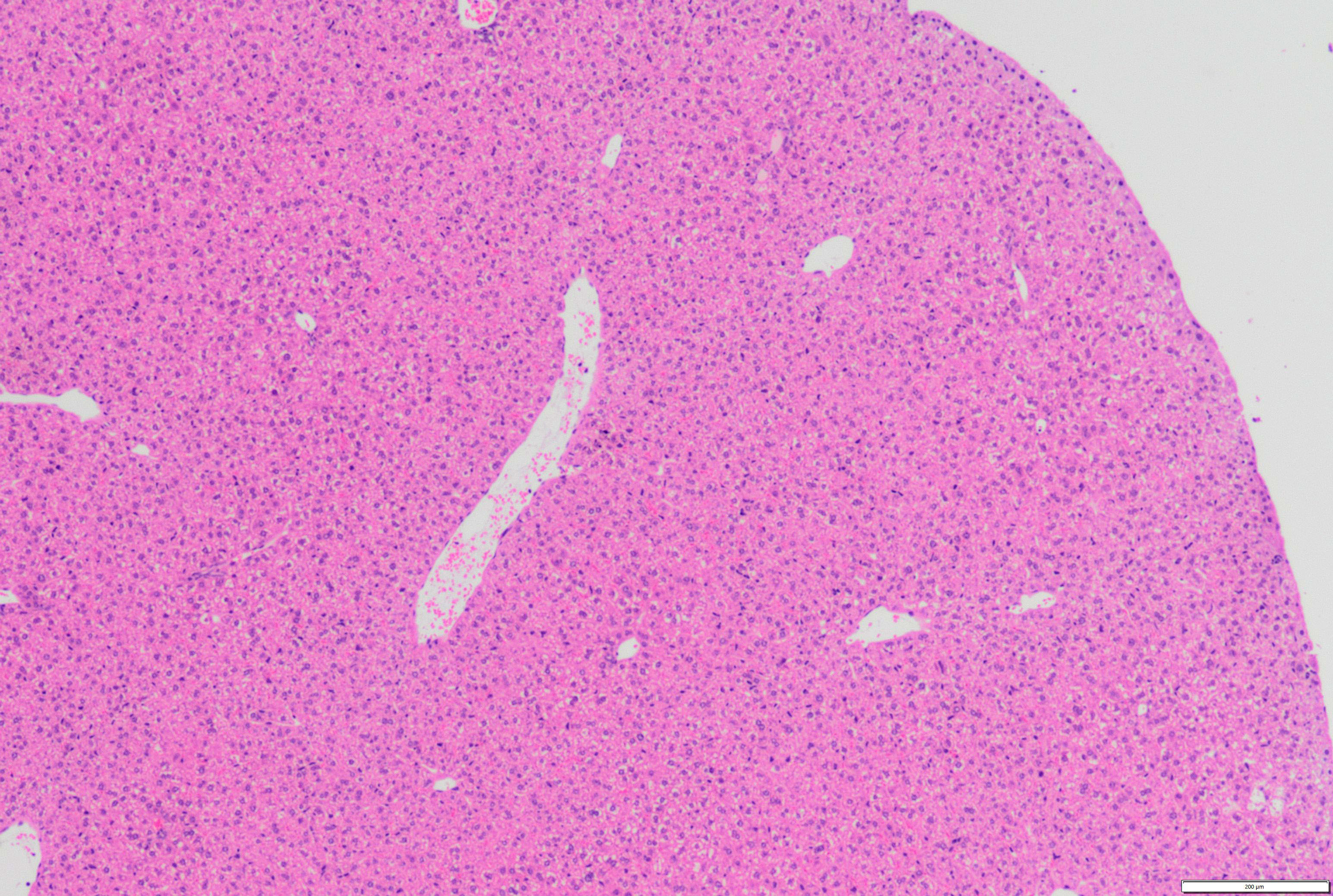

Supplement: Supplementary file 7 — Source data Fig. 6 [file 44321_2024_69_MOESM7_ESM.zip › Figure 6/IMAGES for Fig.6D/H&E Liver KO CBDCA 5x.jpg]

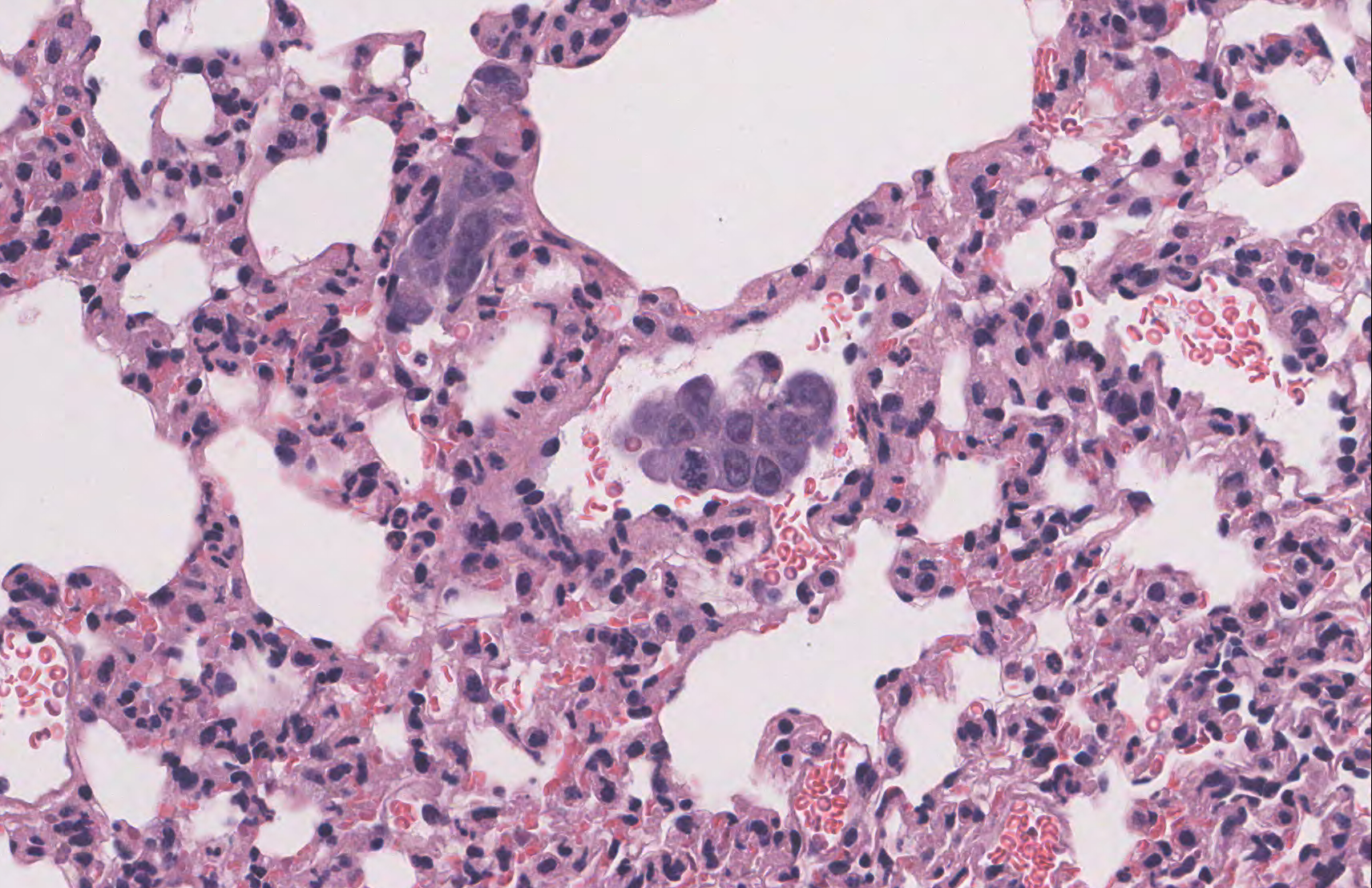

Supplement: Supplementary file 7 — Source data Fig. 6 [file 44321_2024_69_MOESM7_ESM.zip › Figure 6/IMAGES for Fig.6J/PDX UNT lung 40x.tif]

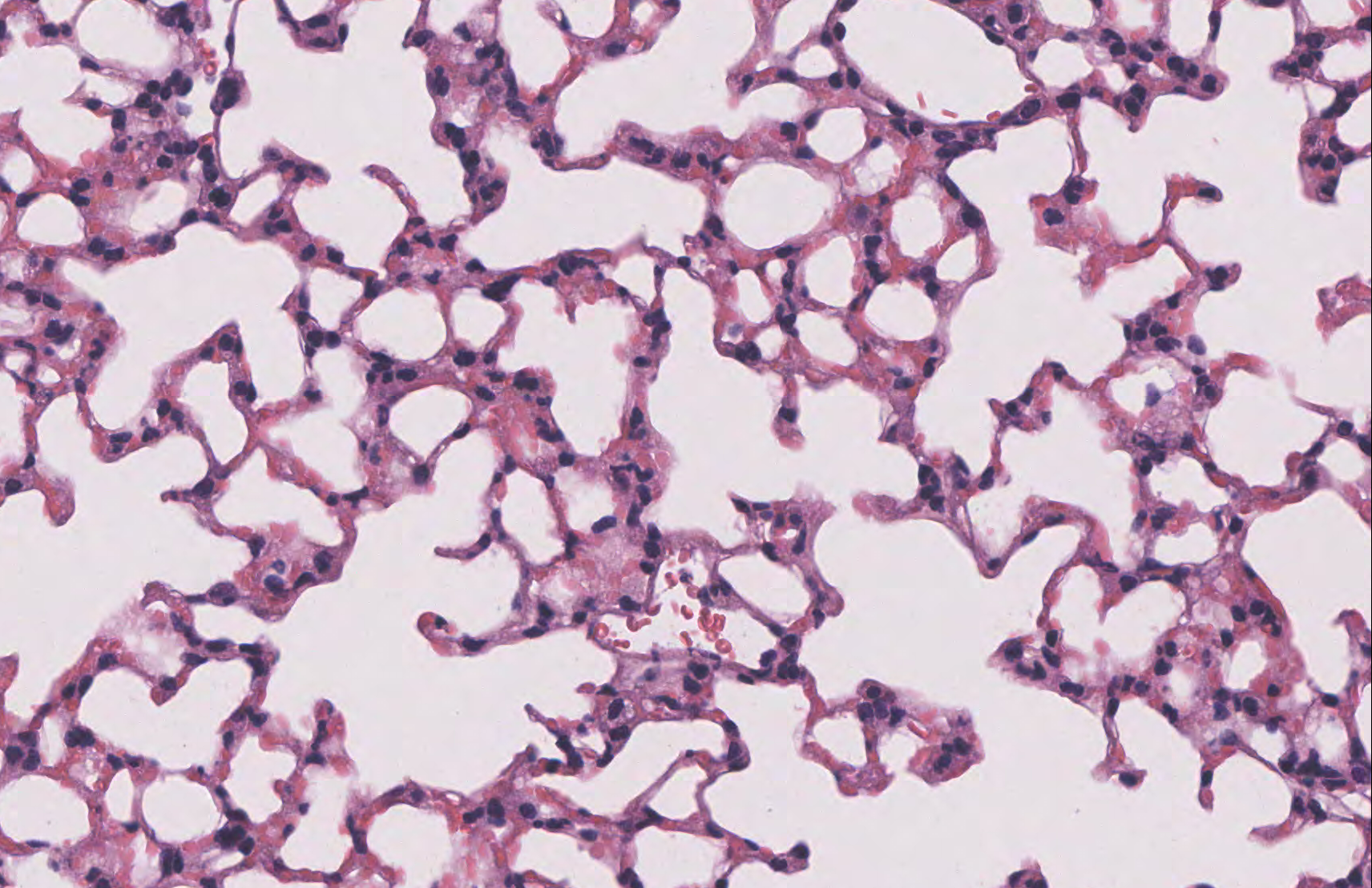

Supplement: Supplementary file 7 — Source data Fig. 6 [file 44321_2024_69_MOESM7_ESM.zip › Figure 6/IMAGES for Fig.6J/PDX COMBO lung 40x.tif]

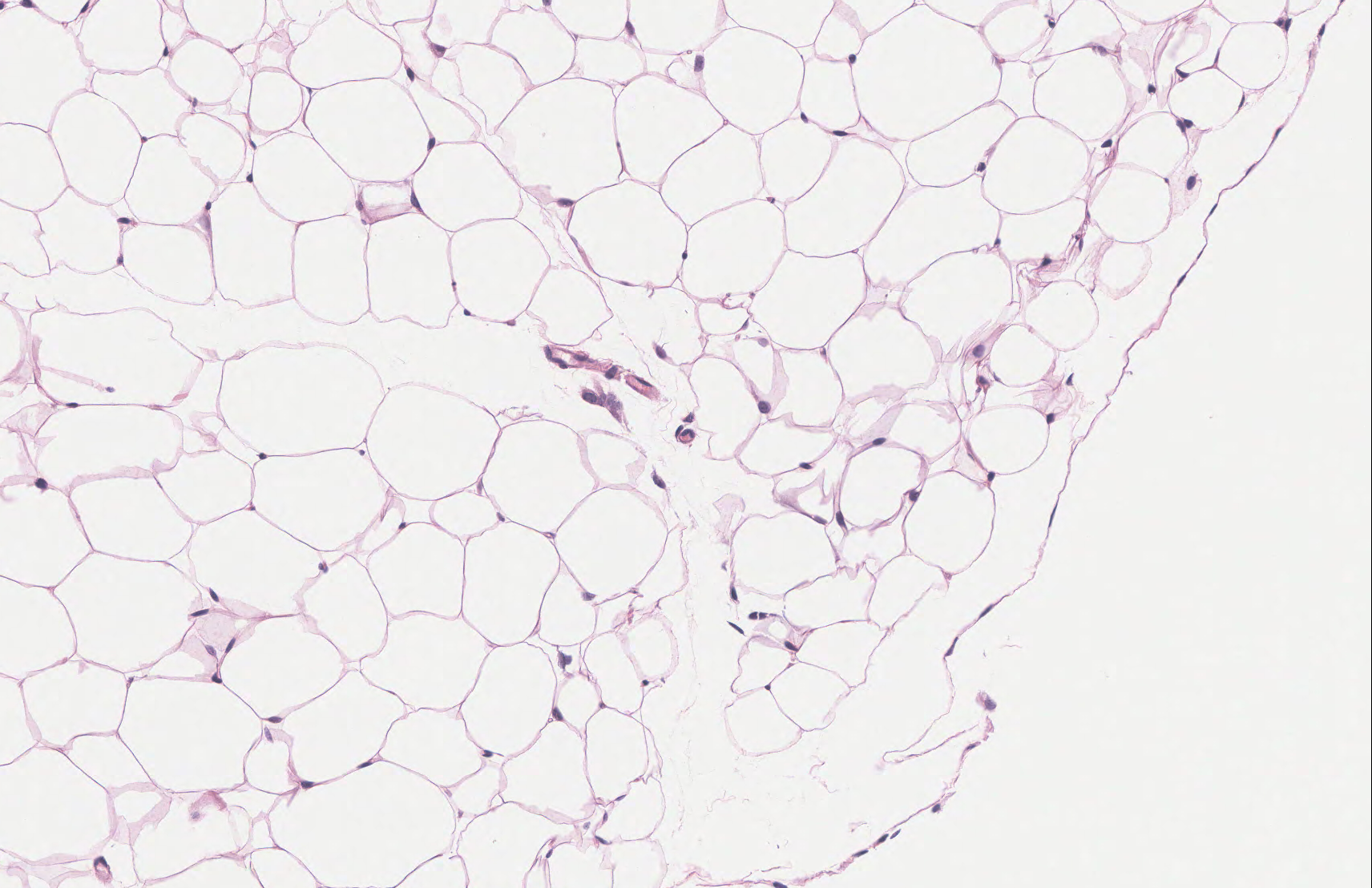

Supplement: Supplementary file 7 — Source data Fig. 6 [file 44321_2024_69_MOESM7_ESM.zip › Figure 6/IMAGES for Fig.6J/PDX COMBO omentum 20x.tif]

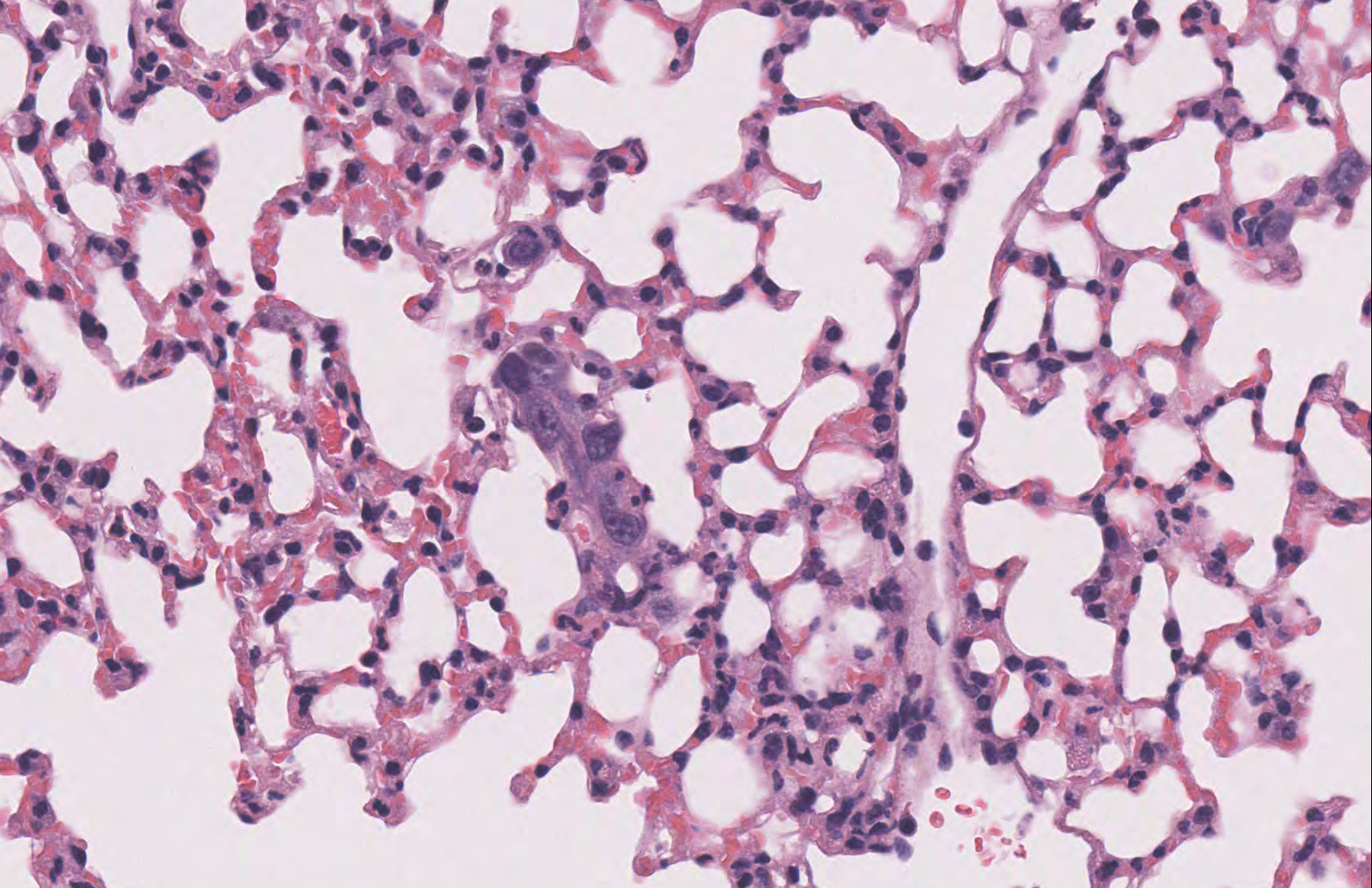

Supplement: Supplementary file 7 — Source data Fig. 6 [file 44321_2024_69_MOESM7_ESM.zip › Figure 6/IMAGES for Fig.6J/PDX Blocking Ab lung 40x.tif]

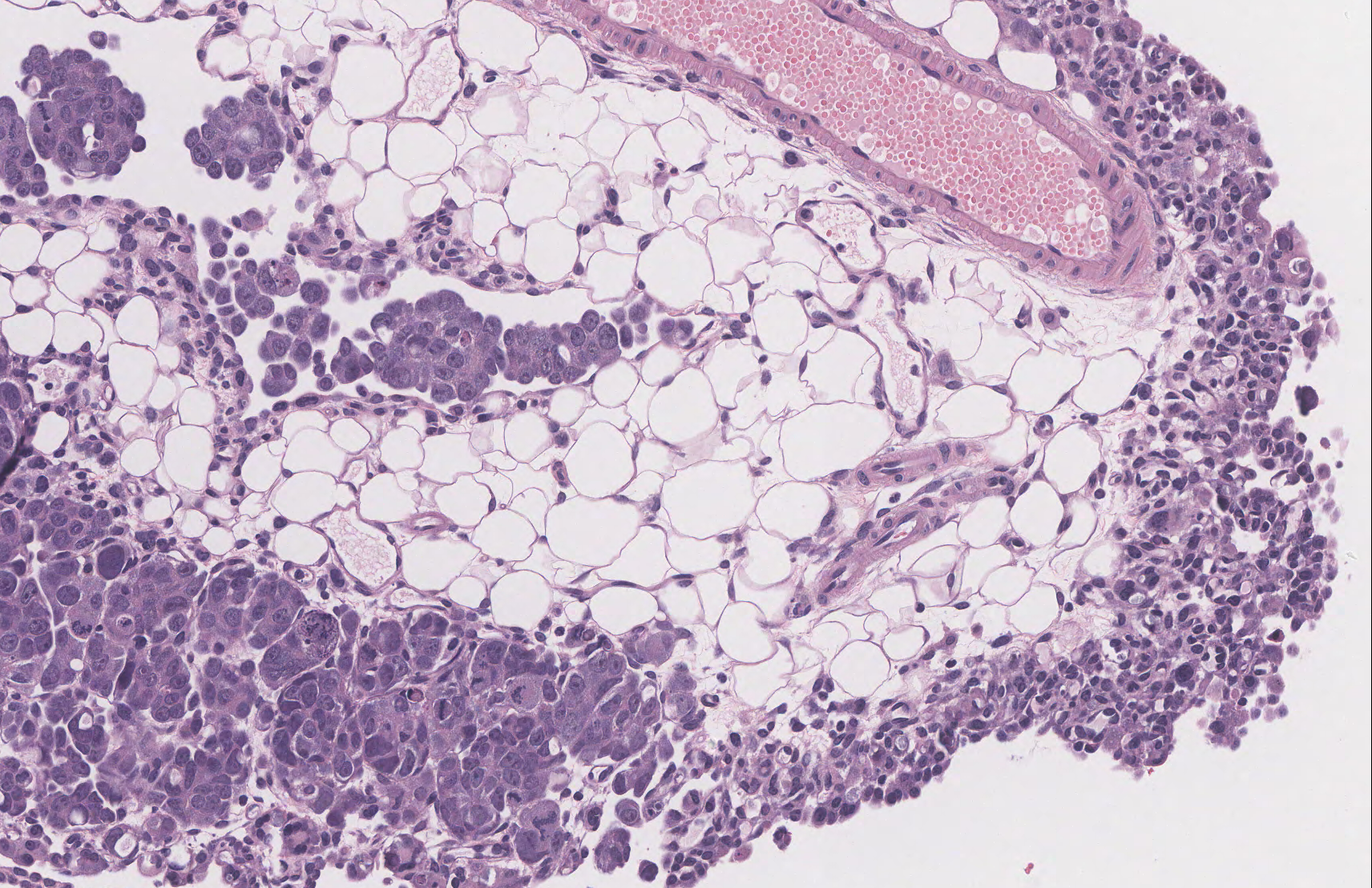

Supplement: Supplementary file 7 — Source data Fig. 6 [file 44321_2024_69_MOESM7_ESM.zip › Figure 6/IMAGES for Fig.6J/PDX CBDCA omentum 20x.tif]

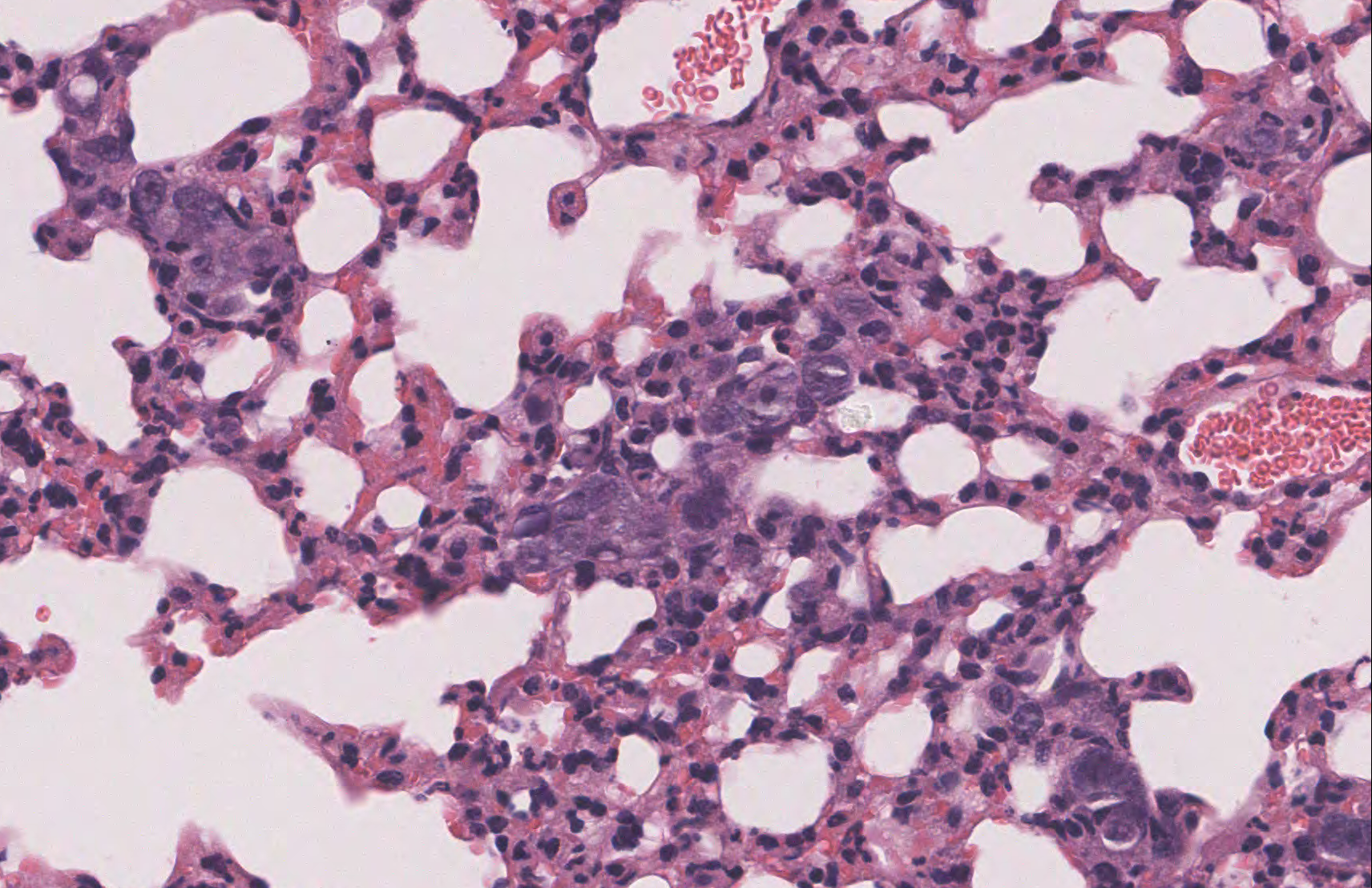

Supplement: Supplementary file 7 — Source data Fig. 6 [file 44321_2024_69_MOESM7_ESM.zip › Figure 6/IMAGES for Fig.6J/PDX CBDCA lung 40x.tif]

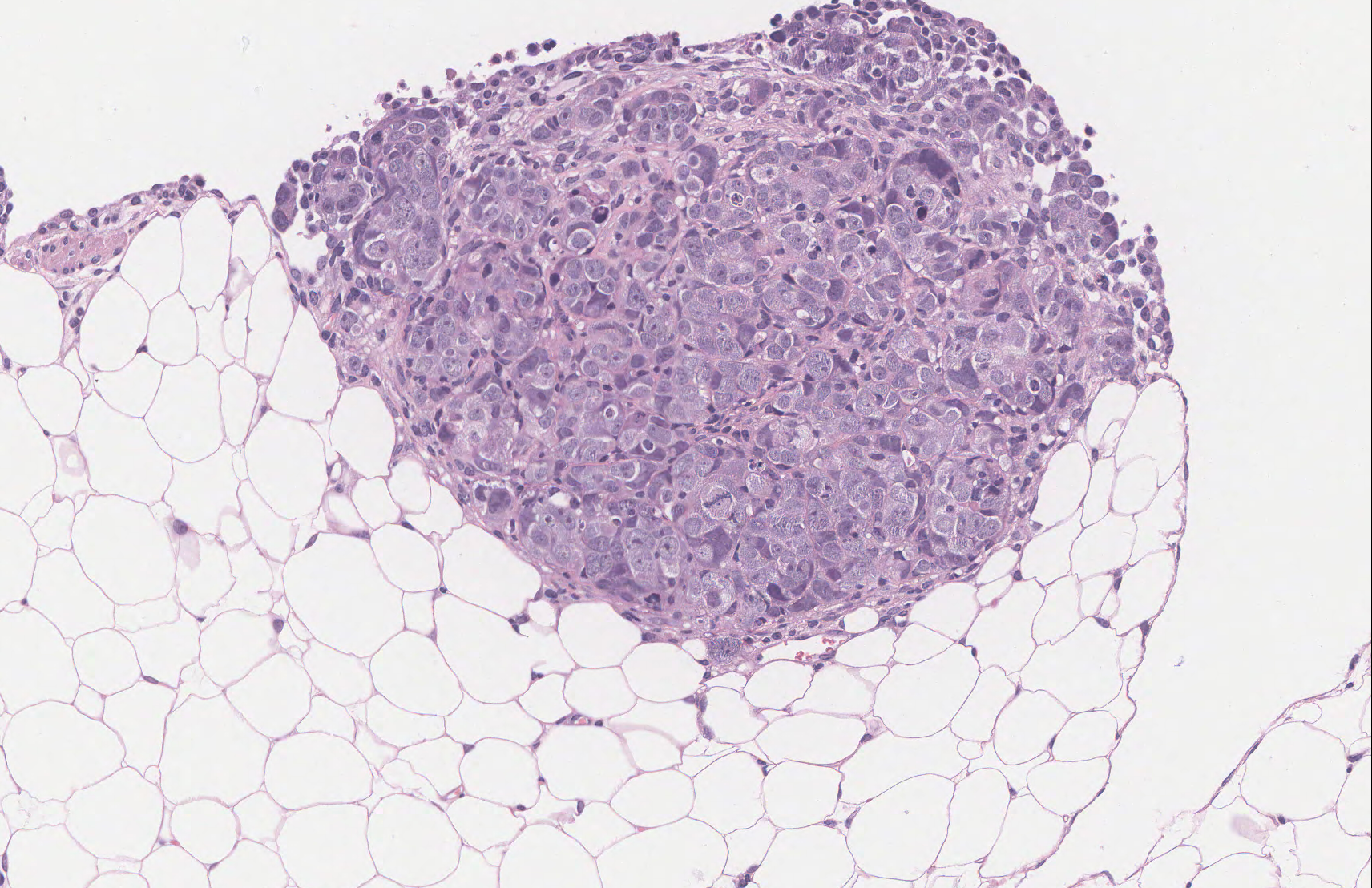

Supplement: Supplementary file 7 — Source data Fig. 6 [file 44321_2024_69_MOESM7_ESM.zip › Figure 6/IMAGES for Fig.6J/PDX Blocking Ab omentum 20x.tif]

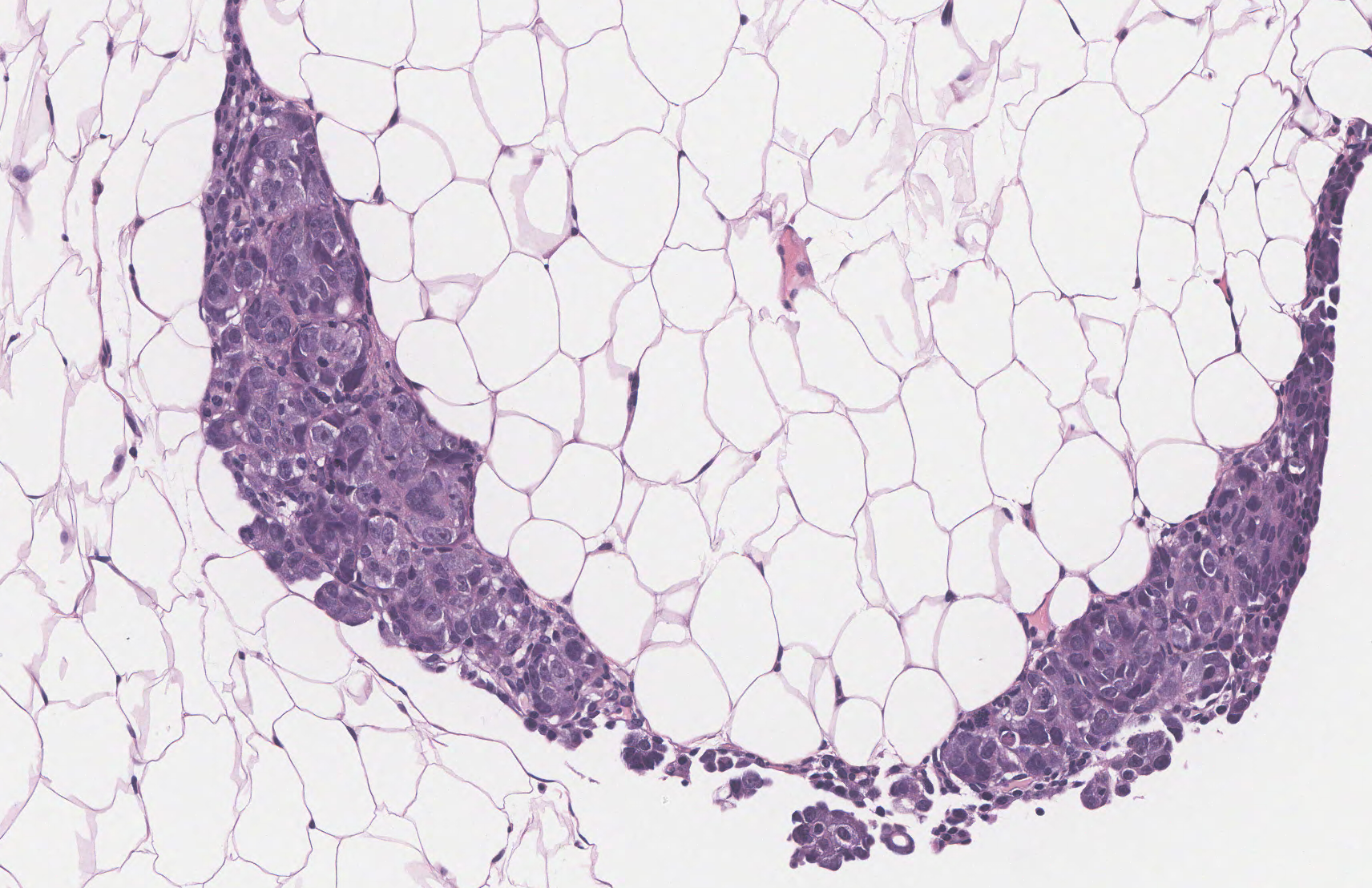

Supplement: Supplementary file 7 — Source data Fig. 6 [file 44321_2024_69_MOESM7_ESM.zip › Figure 6/IMAGES for Fig.6J/PDX UNT omentum 20x.tif]

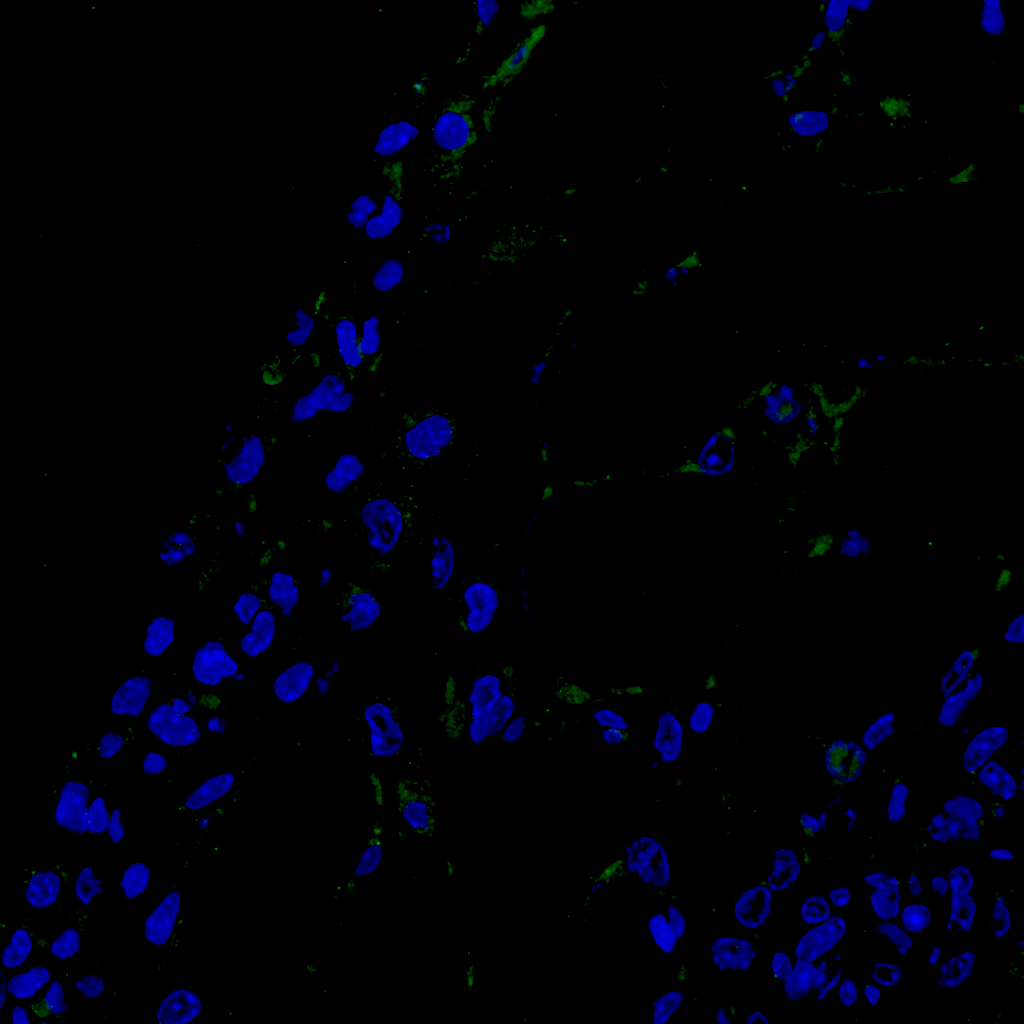

Supplement: Supplementary file 7 — Source data Fig. 6 [file 44321_2024_69_MOESM7_ESM.zip › Figure 6/IMAGES for Fig.6M/COMBO b MERGE.png]

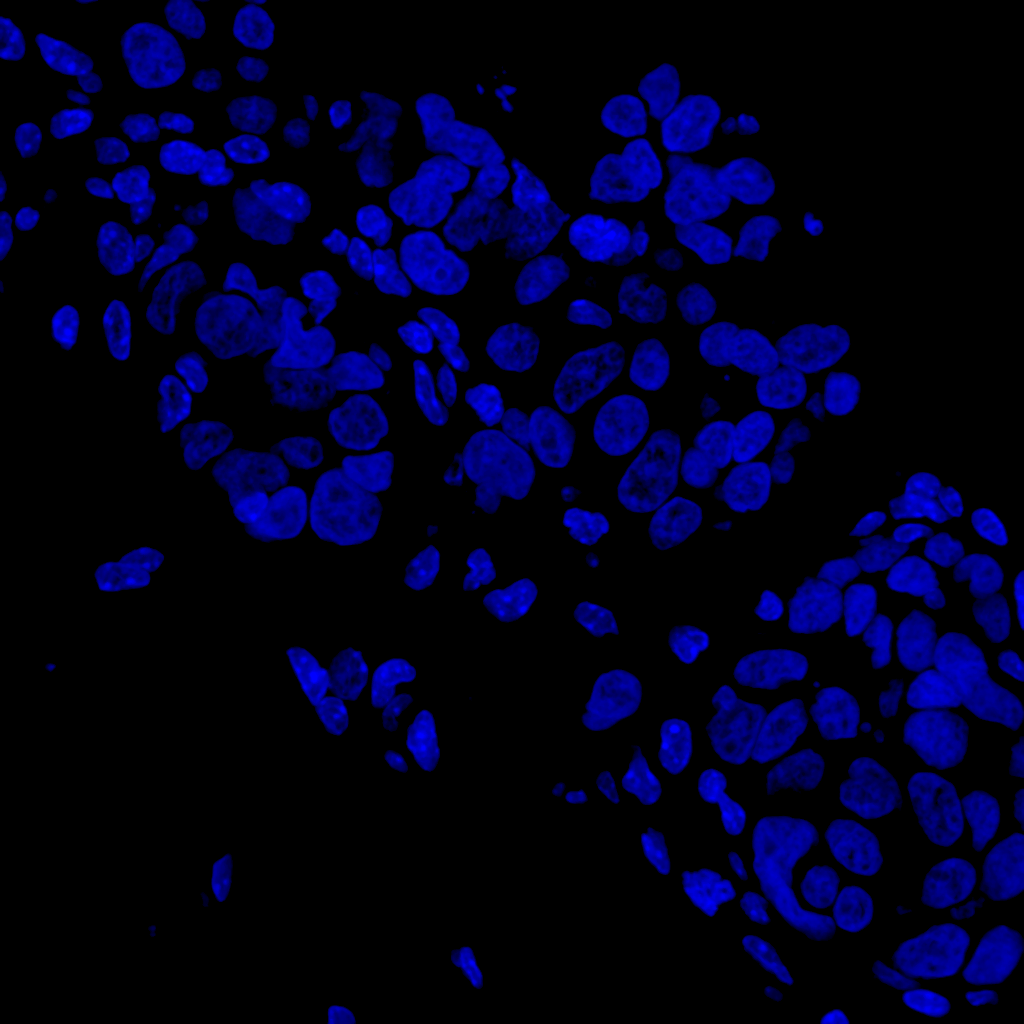

Supplement: Supplementary file 7 — Source data Fig. 6 [file 44321_2024_69_MOESM7_ESM.zip › Figure 6/IMAGES for Fig.6M/CBDCA f topro.png]

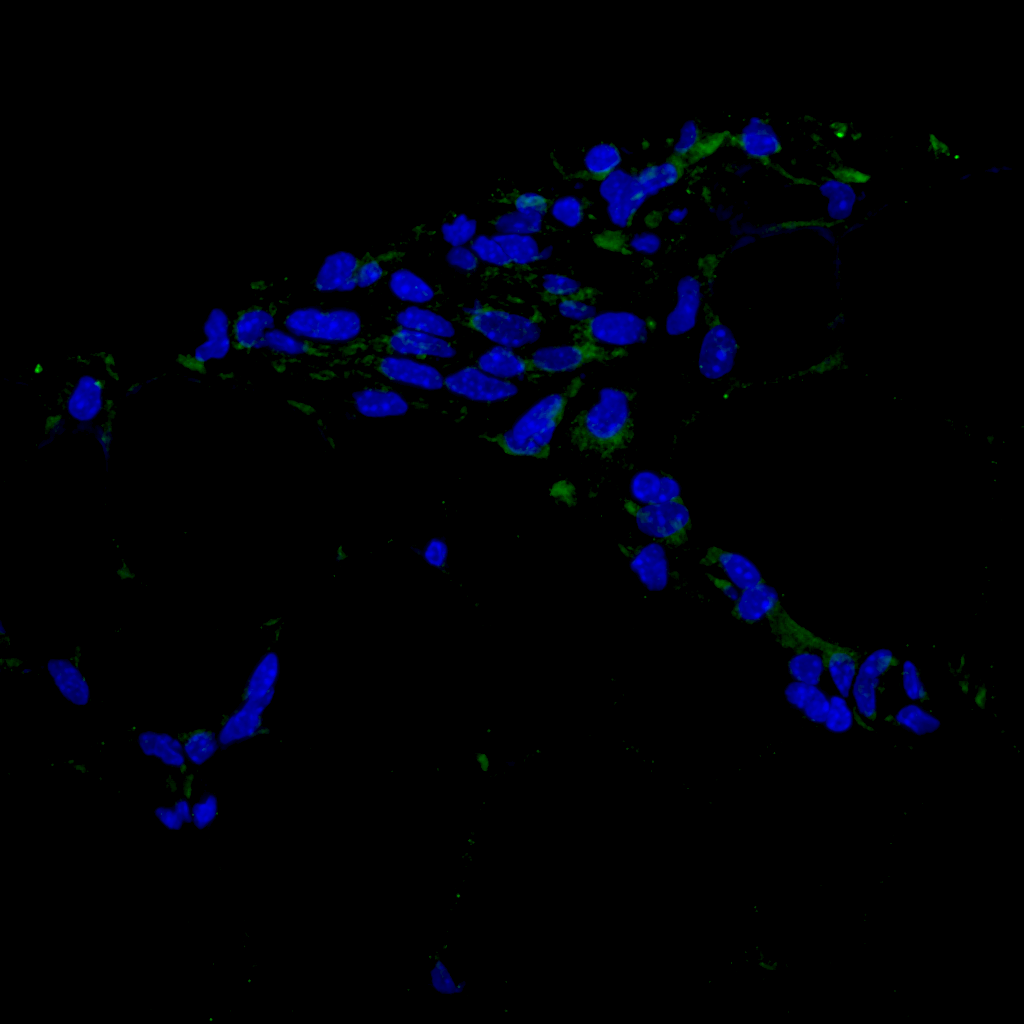

Supplement: Supplementary file 7 — Source data Fig. 6 [file 44321_2024_69_MOESM7_ESM.zip › Figure 6/IMAGES for Fig.6M/P5G a merge.png]

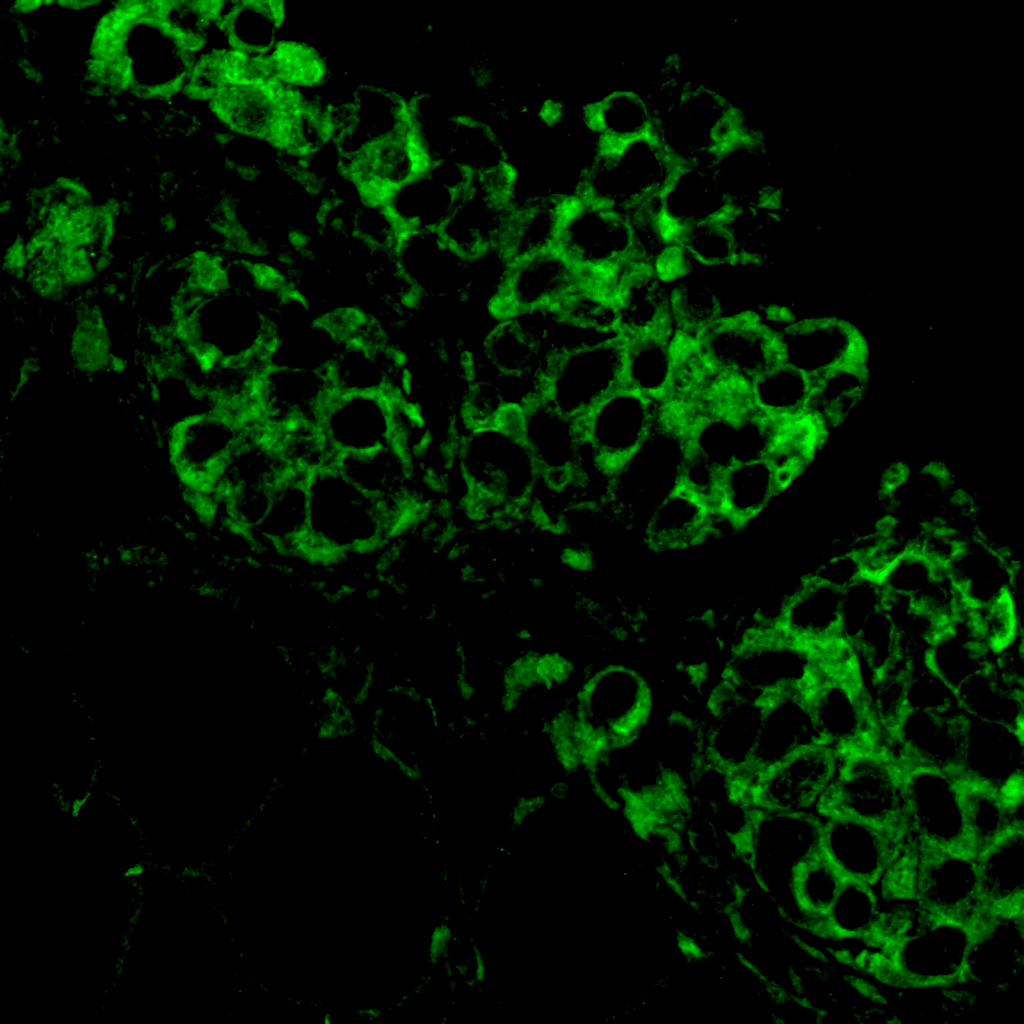

Supplement: Supplementary file 7 — Source data Fig. 6 [file 44321_2024_69_MOESM7_ESM.zip › Figure 6/IMAGES for Fig.6M/CBDCA f snail.png]

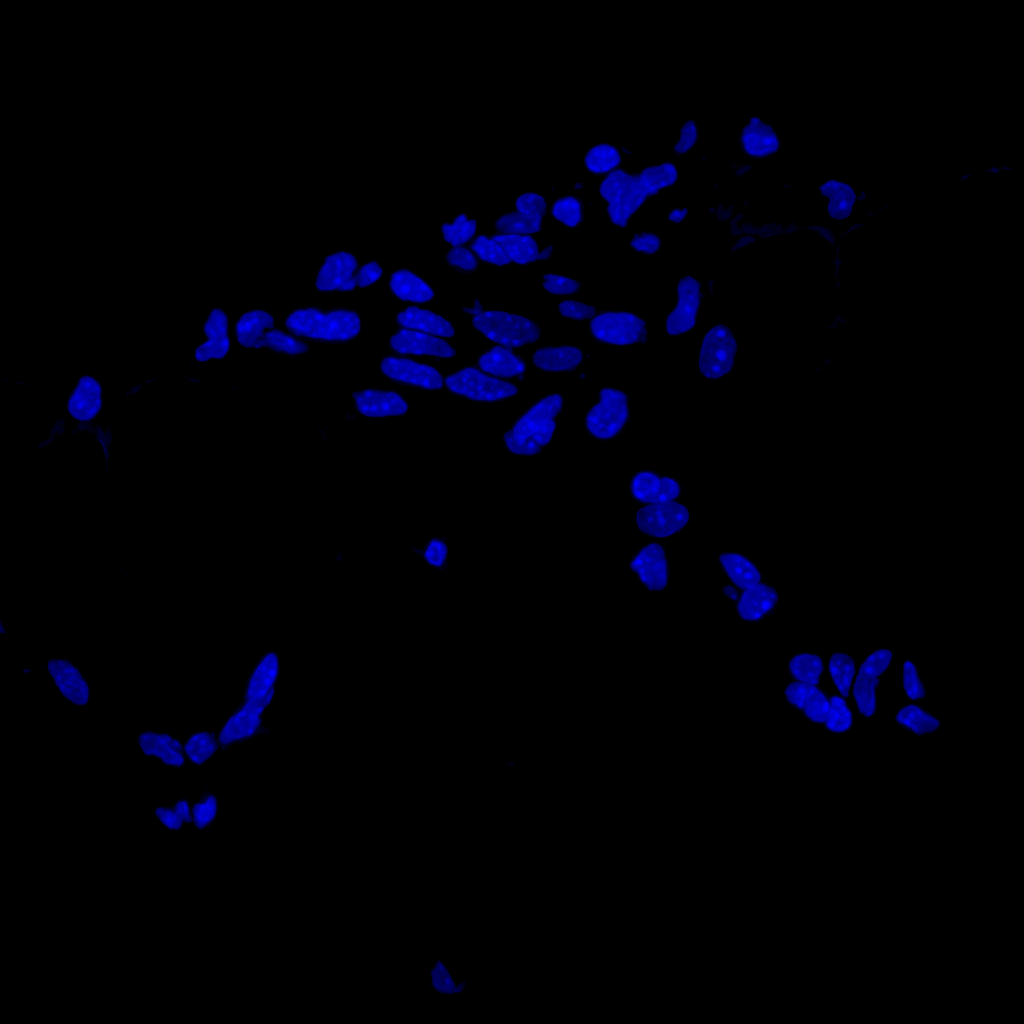

Supplement: Supplementary file 7 — Source data Fig. 6 [file 44321_2024_69_MOESM7_ESM.zip › Figure 6/IMAGES for Fig.6M/P5G a topro.png]

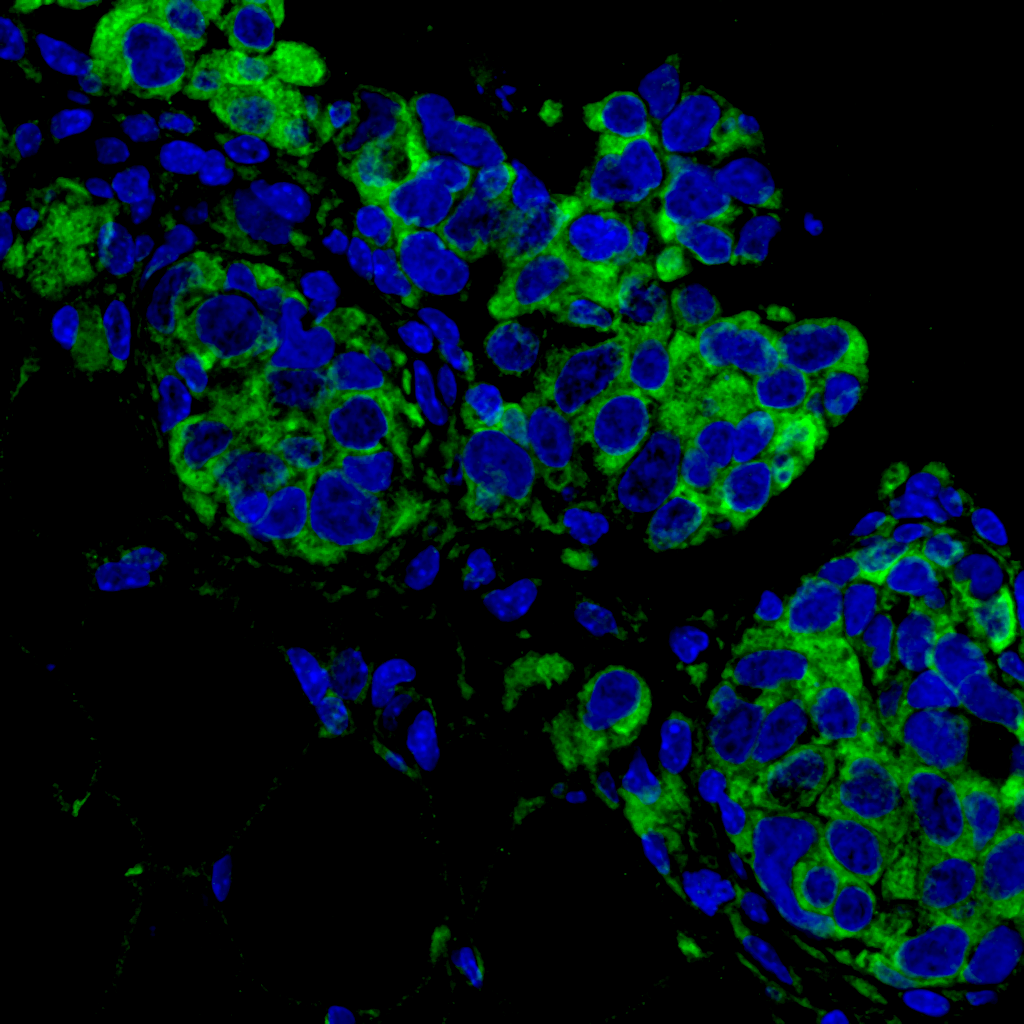

Supplement: Supplementary file 7 — Source data Fig. 6 [file 44321_2024_69_MOESM7_ESM.zip › Figure 6/IMAGES for Fig.6M/CBDCA f merge.png]

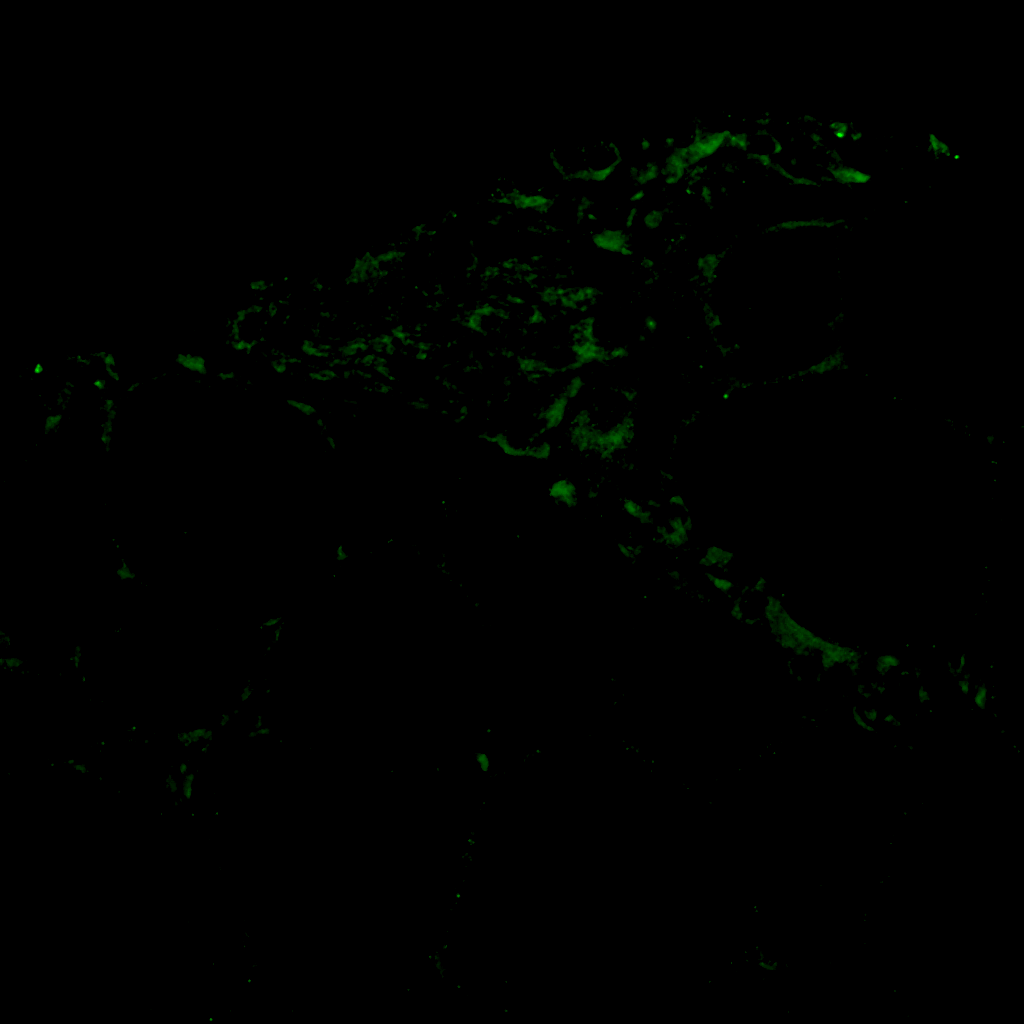

Supplement: Supplementary file 7 — Source data Fig. 6 [file 44321_2024_69_MOESM7_ESM.zip › Figure 6/IMAGES for Fig.6M/P5G a snail.png]

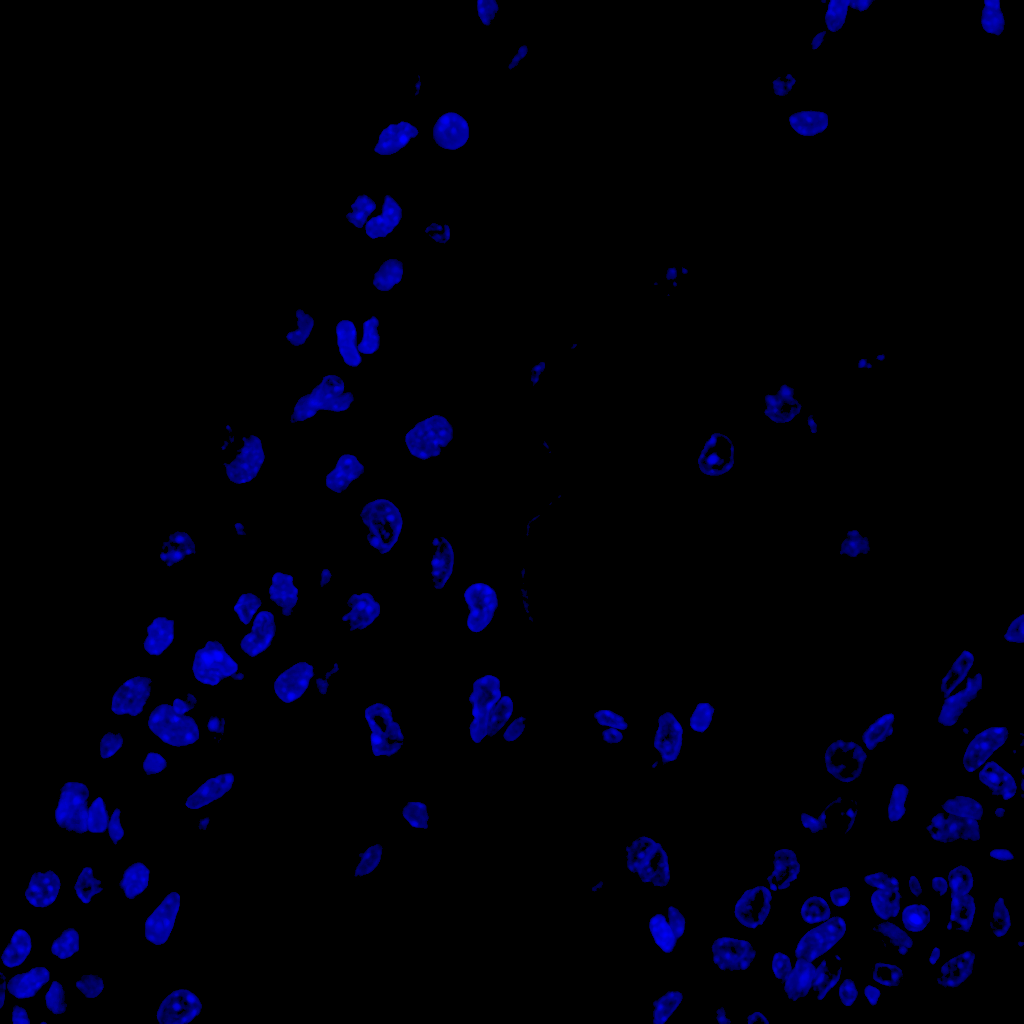

Supplement: Supplementary file 7 — Source data Fig. 6 [file 44321_2024_69_MOESM7_ESM.zip › Figure 6/IMAGES for Fig.6M/COMBO b topro.png]

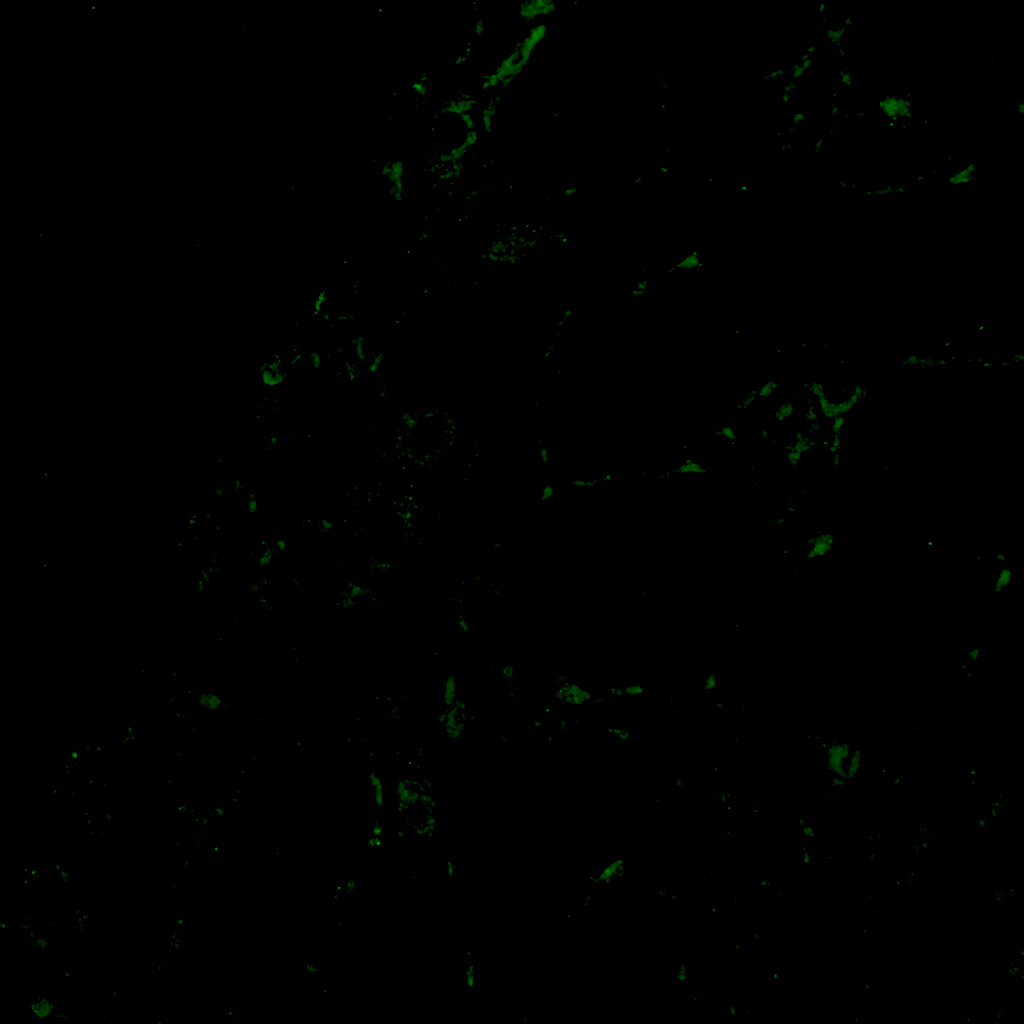

Supplement: Supplementary file 7 — Source data Fig. 6 [file 44321_2024_69_MOESM7_ESM.zip › Figure 6/IMAGES for Fig.6M/COMBO b SNAIL.png]

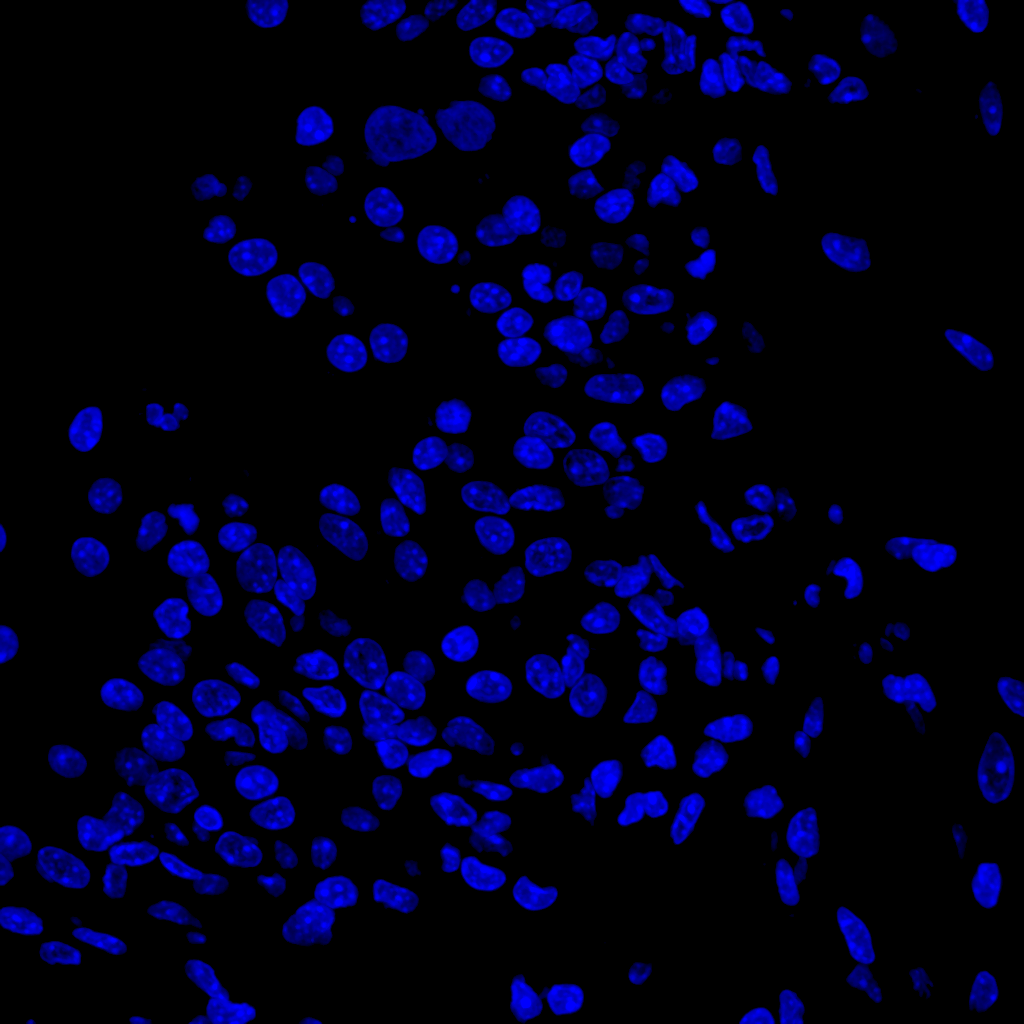

Supplement: Supplementary file 7 — Source data Fig. 6 [file 44321_2024_69_MOESM7_ESM.zip › Figure 6/IMAGES for Fig.6M/UNT g topro.png]

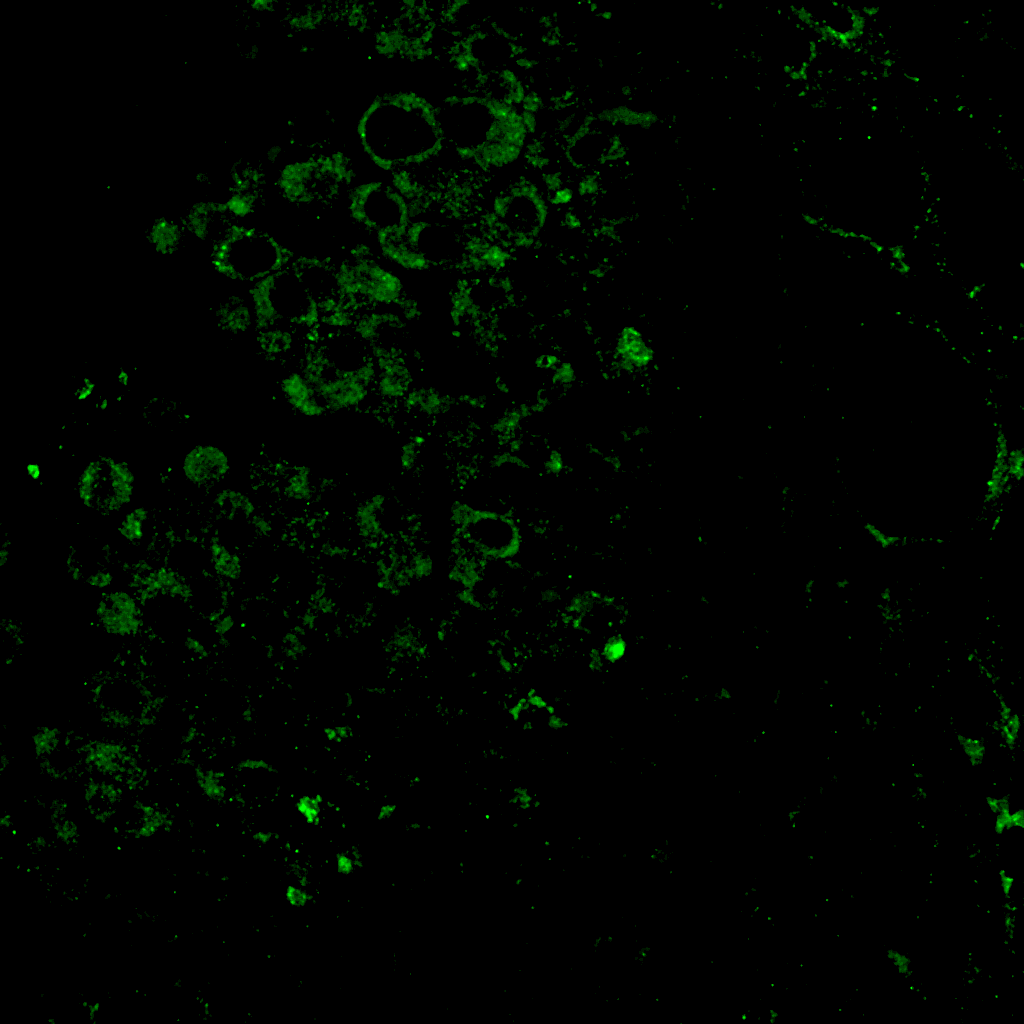

Supplement: Supplementary file 7 — Source data Fig. 6 [file 44321_2024_69_MOESM7_ESM.zip › Figure 6/IMAGES for Fig.6M/UNT g snail.png]

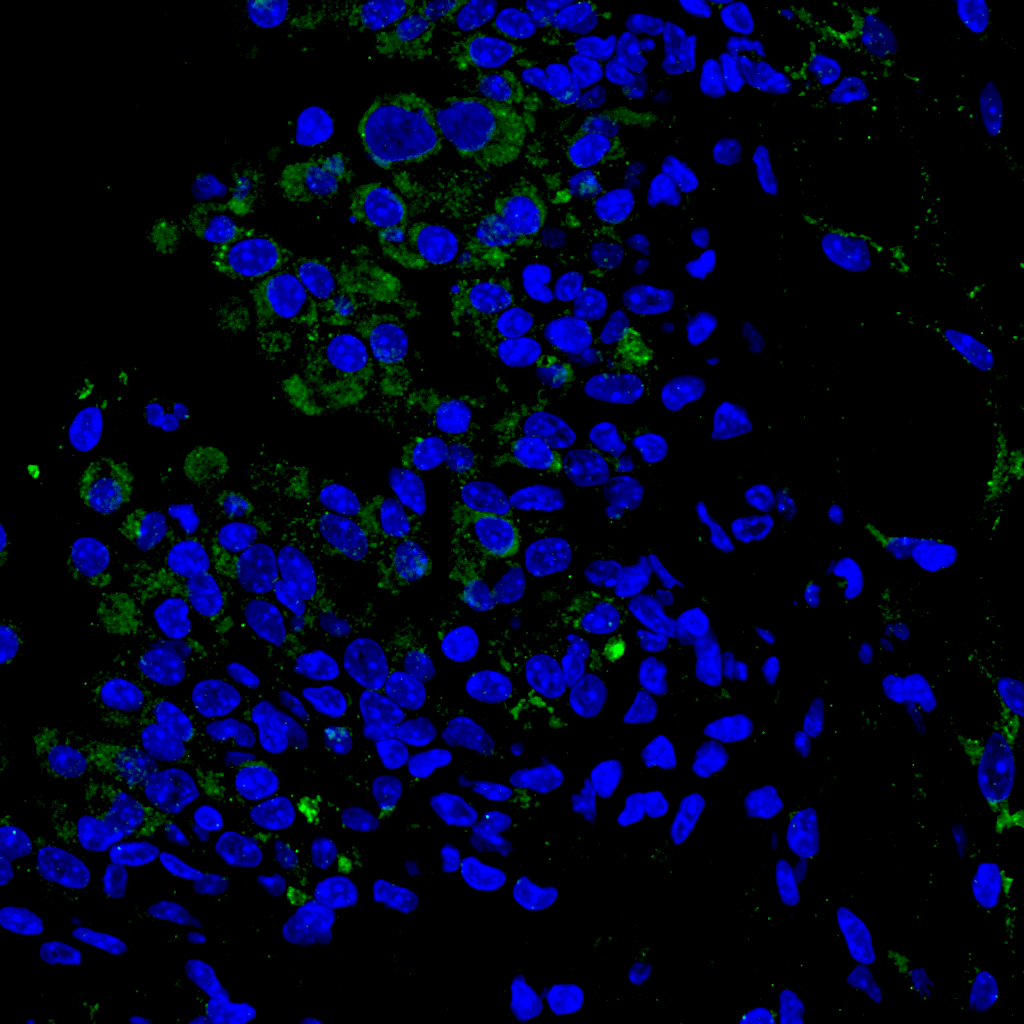

Supplement: Supplementary file 7 — Source data Fig. 6 [file 44321_2024_69_MOESM7_ESM.zip › Figure 6/IMAGES for Fig.6M/UNT g merge.png]
