# Supplementary material for: Platinum-induced upregulation of ITGA6 promotes chemoresistance and spreading in ovarian cancer
Source: EMBO Mol Med. 2024 Apr 24;16(5):7. doi: 10.1038/s44321-024-00069-3 (PMC11099142; doi:10.1038/s44321-024-00069-3)
Supplement: Supplementary file 10 — Expanded View Figures [file 44321_2024_69_MOESM10_ESM.pdf]

Expanded View Figures

Figure EV1. ITGA6 is commonly overexpressed in EOC PT-res cells.

(**A**) Graphs reporting the percentage of cell adhesion on different extracellular matrices of TOV-112D and OVSAHO PT-sen and PT-res pools measured by the quantitative cell adhesion CAFCA assay ($n = 2$ in sextuplicate). Coll IV = Type IV Collagen; Coll I = Type I Collagen; FN = Fibronectin; LNs = Laminins; VN= Vitronectin. (**B**) Tables reporting the percentage of cells positive for the indicated integrins as measured by flow cytometry (FACS) in PT-sen and PT-res pools of TOV-112D and OVSAHO cells (ITGA1 = Integrin α1, ITGA2 = Integrin α2, ITGA5 = Integrin α5, ITGA6 = Integrin α6, ITGAV = Integrin αV, ITGB1 = Integrin β1, ITGB3 = Integrin β3, ITGB4 = Integrin β4). (**C**) FACS analyses of the expression profile of ITGB1, ITGB4, and ITGA6 integrins in OVSAHO PT-sen and PT-res clones. (**D**) Graphs reporting the percentage of cell adhesion on different extracellular matrices of the indicated TOV-112D and OVSAHO PT-sen and PT-res clones. ($n = 2$ in sextuplicate). (**E**) Western blot analysis evaluating the expression of ITGA6 protein in tumor samples from the same patients taken at diagnosis (Pre) or at recurrence after chemotherapy (Post). Tubulin was used as loading control. (**F**) qRT-PCR analysis of ITGA6 mRNA expression in different EOC PT-sen and PT-res cells. (**G**) Western blot analysis of ITGA6 expression in TOV-112D PT-sen and PT-res clone exposed to a time-course treatment with CHX for the indicated times. Vinculin was used as loading control. (**H**) Graph reporting the expression of ITGA6 in OVSAHO PT-sen and PT-res cells treated or not with CDDP for 2 h. mRNA was analyzed by qRT-PCR at the indicated time points after CDDP removal. (**I**) Graphs reporting qRT-PCR analysis of mRNA expression of ITGA6 in TOV-112D ITGA6^{HIGH} and ITGA6^{LOW} subpopulations treated or not with CDDP. In (**F**), (**H**), and (**I**), mRNA levels were analyzed in triplicate and normalized to actin housekeeping gene and expressed in arbitrary units. In all the graph of the figure statistical significance was determined by a two-tailed, unpaired Student's t-test (Exact *p* values were reported on graphs). Bars represent Standard Deviation.

© The Author(s)

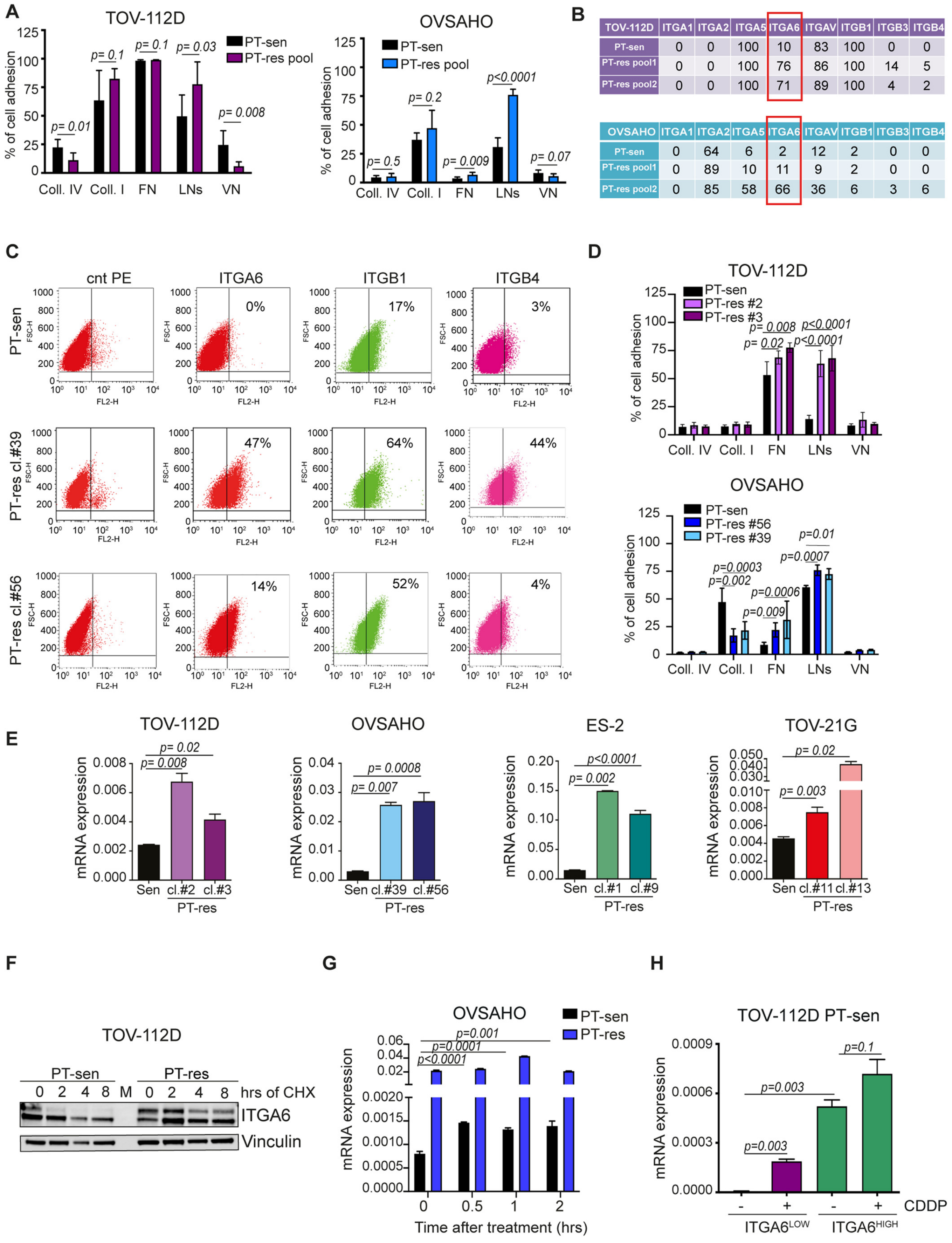

TOV-112D	ITGA1	ITGA2	ITGA5	ITGA6	ITGAV	ITGB1	ITGB3	ITGB4
PT-sen	0	0	100	10	83	100	0	0
PT-res pool1	0	0	100	76	86	100	14	5
PT-res pool2	0	0	100	71	89	100	4	2

OVSAHO	ITGA1	ITGA2	ITGA5	ITGA6	ITGAV	ITGB1	ITGB3	ITGB4
PT-sen	0	64	6	2	12	2	0	0
PT-res pool1	0	89	10	11	9	2	0	0
PT-res pool2	0	85	58	66	36	6	3	6

© The Author(s)

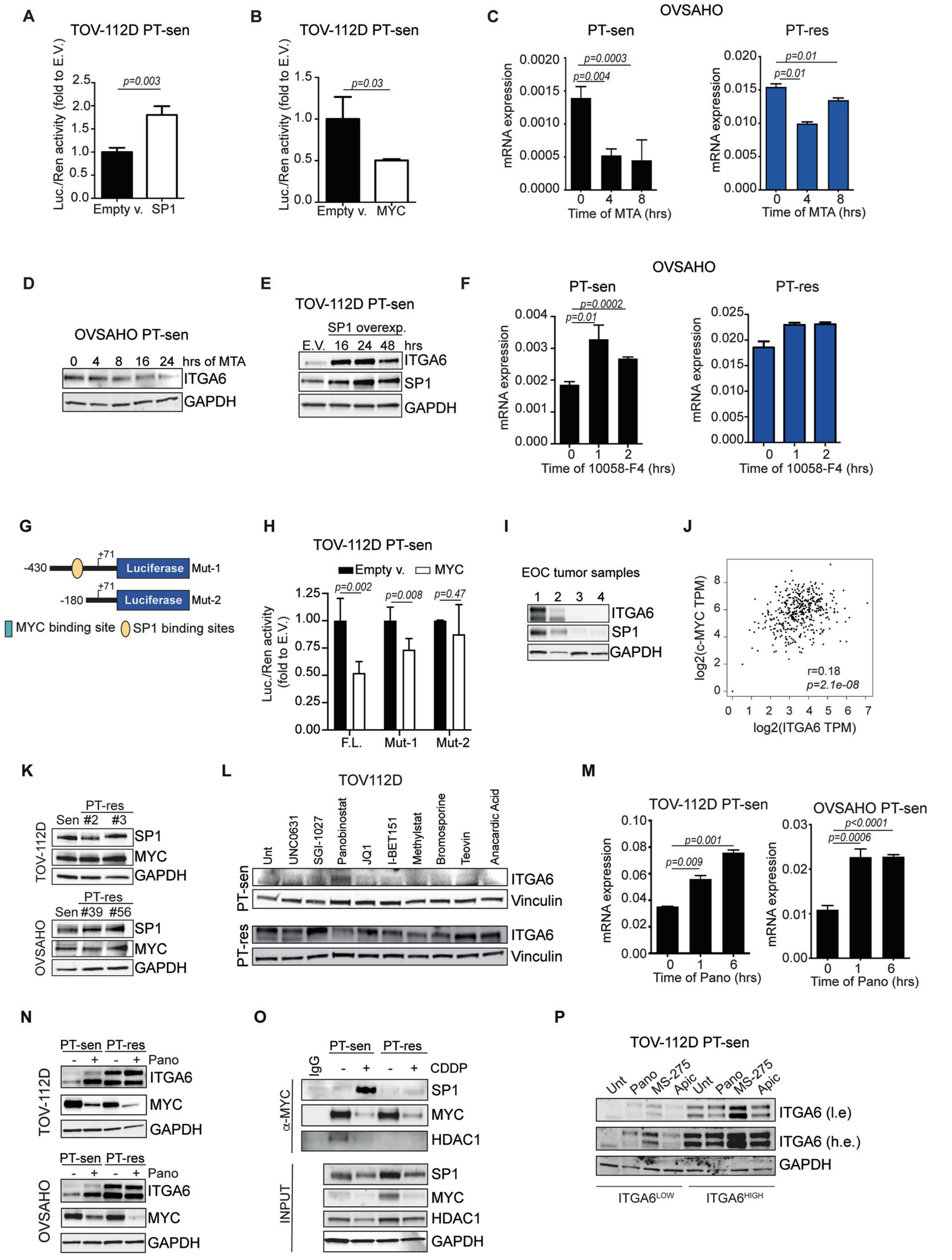

A
TOV-112D PT-sen
Luc./Ren activity (fold to E.V.)
p=0.003
Empty v. SP1
B
TOV-112D PT-sen
Luc./Ren activity (fold to E.V.)
p=0.03
Empty v. MYC
C
OVSAHO
PT-sen
PT-res
mRNA expression
p=0.0003
p=0.004
p=0.01
p=0.01
Time of MTA (hrs)
D
OVSAHO PT-sen
0 4 8 16 24 hrs of MTA
ITGA6
GAPDH
E
TOV-112D PT-sen
SP1 overexp.
E.V. 16 24 48 hrs
ITGA6
SP1
GAPDH
F
OVSAHO
PT-sen
PT-res
mRNA expression
p=0.0002
p=0.01
Time of 10058-F4 (hrs)
G
-430
+71
Luciferase
Mut-1
-180
Mut-2
MYC binding site
SP1 binding sites
H
TOV-112D PT-sen
Empty v.
MYC
Luc./Ren activity (fold to E.V.)
p=0.002
p=0.008
p=0.47
F.L. Mut-1 Mut-2
I
EOC tumor samples
1 2 3 4
ITGA6
SP1
GAPDH
J
log2(c-MYC TPM)
log2(ITGA6 TPM)
r=0.18
p=2.1e-08
K
PT-res
Sen #2 #3
TOV-112D
SP1
MYC
GAPDH
Sen #39 #56
OVSAHO
L
TOV112D
Unt
UNC0631
SGI-1027
Panobinostat
JQ1
I-BET151
Methylstat
Bromosporine
Teovin
Anacardic Acid
PT-sen
PT-res
ITGA6
Vinculin
M
TOV-112D PT-sen
OVSAHO PT-sen
mRNA expression
p=0.001
p=0.009
p<0.0001
p=0.0006
Time of Pano (hrs)
N
PT-sen PT-res
Pano
TOV-112D
OVSAHO
ITGA6
MYC
GAPDH
O
IgG
PT-sen PT-res
CDDP
α-MYC
INPUT
SP1
MYC
HDAC1
GAPDH
P
TOV-112D PT-sen
Unt Pano MS-275 Apic Unt Pano MS-275 Apic
ITGA6 (l.e)
ITGA6 (h.e.)
GAPDH
ITGA6LOW
ITGA6HIGH

Figure EV2. SP1/c-Myc/HDAC1 axis regulates ITGA6 transcription after CDDP treatment.

(**A**, **B**) Graphs reporting the luciferase activity measured in TOV-112D PT-sen transfected with ITGA6 promoter full length (FL) together with SP1 (**A**) or c-Myc (MYC) (**B**) vectors. In A and B data are the mean (±SD) of three independent experiments. (**C**) Graphs reporting the expression of ITGA6 in OVSAHO PT-sen (left) and PT-res (right) cells treated with SP1 inhibitor (Mithramycin, MTA). mRNA expression was analyzed by qRT-PCR at the indicated time points. (**D**) Western blot analysis of ITGA6 expression in OVSAHO PT-sen cells treated with SP1 inhibitor, MTA for the indicated time points. (**E**) Western blot analysis of ITGA6 expression in TOV-112D PT-sen cells transfected with SP1 vector. Whole lysates were collected at the indicated time points after transfection. (**F**) Graphs reporting the expression of ITGA6 in OVSAHO PT-sen (left) and PT-res (right) cells treated with the c-Myc inhibitor (10058-F4)). mRNA was analyzed by qRT-PCR at the indicated time points. (**G**) ITGA6 promoter deletion mutants lacking c-Myc (Mut-1) or both c-Myc and SP1 binding sites (Mut-2) generated for this study. (**H**) Graph reporting the luciferase activity measured in TOV-112D PT-sen co-transfected with the full length (F.L.) or deletion mutants and the empty (black bars) or c-Myc (MYC, white bars) expression vectors. Data are the mean (±SD) of three independent experiments. (**I**) Western blot analysis of ITGA6 and SP1 protein expression in EOC tumor samples collected in our Institute (see Appendix Table S1). (**J**) Spearman's correlation analysis between ITGA6 and c-Myc mRNA expression in TCGA ovarian cancer dataset ($n = 489$ samples) using the GEPIA online tool. (**K**) Western blot analysis of c-Myc and SP1 protein expression in TOV-112D and OVSAHO PT-sen and PT-res clones. (**L**) Western blot analysis of ITGA6 protein expression in TOV-112D PT-sen and PT-res cells treated with a panel of epigenetic modifiers inhibitors for 24 h (UNC0631 and SGI-1027 = methyl transferase inhibitors; Panobinostat = pan-HDAC inhibitor; JQ1, iBET151 and Bromosporine = BET inhibitors; Methylstat = de-methyltransferase inhibitor; Tenovin= SIRTs inhibitor; Anacardic acid = HATs inhibitor). (**M**) Graph reporting the mRNA expression of ITGA6 in indicated PT-sen EOC cells treated with Panobinostat (Pano) for 1 and 6 h. mRNA expression was analyzed in triplicate in two biological replicates. (**N**) Western blot analysis of c-Myc (MYC) and ITGA6 protein expression in TOV-112D and OVSAHO PT-sen and PT-res clones treated or not with Panobinostat for 24 h. (**O**) Co-immunoprecipitation (Co-IP) analysis of c-Myc, HDAC1, and SP1 in TOV-112D PT-sen and PT-res cells treated or not with CDDP. Input shows the expression of the indicated proteins in the lysates used for IP experiments; IgG represents the control IP using an unrelated antibody. (**P**) Western blot analysis of ITGA6 expression in ITGA6^{HIGH} and ITGA6^{LOW} subpopulations treated with different HDACs inhibitors for 24 h (MS-275 preferentially inhibits HDAC1; Apicidin preferentially inhibits HDAC1 and HDAC3). In all western blots of the figure, GAPDH or Vinculin were used as loading control, as indicated. In (**C**), (**F**), and (**M**), mRNA expression was analyzed in triplicate and normalized to actin housekeeping gene and expressed in arbitrary units. In all the graphs of the figure, statistical significance was determined by a two-tailed, unpaired Student's t-test (Exact *p* values were reported on graphs). Bars represent Standard Deviation.

 © The Author(s)

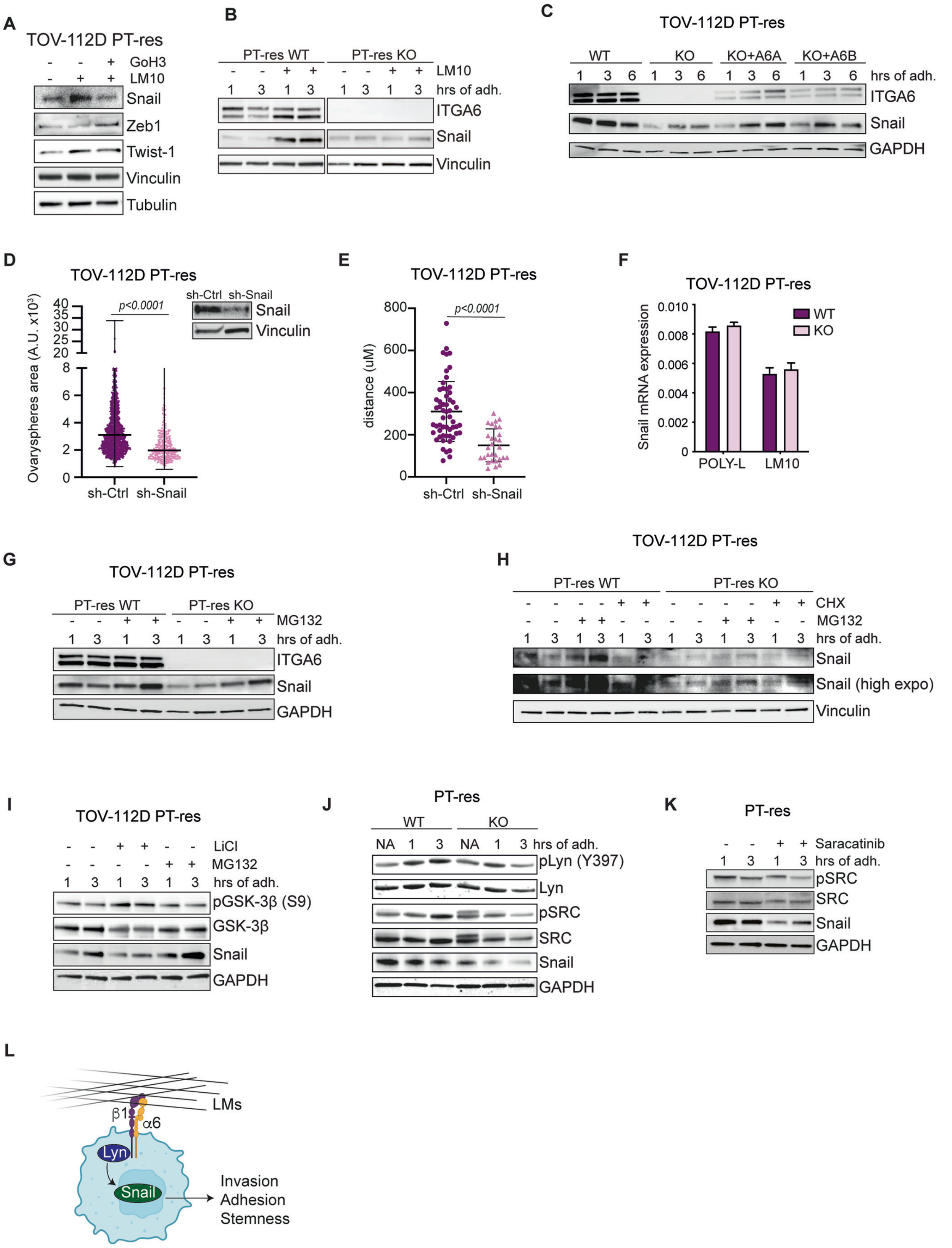
A
TOV-112D PT-res
GoH3
LM10
Snail
Zeb1
Twist-1
Vinculin
Tubulin
B
PT-res WT
PT-res KO
LM10
hrs of adh.
ITGA6
Snail
Vinculin
C
TOV-112D PT-res
WT
KO
KO+A6A
KO+A6B
hrs of adh.
ITGA6
Snail
GAPDH
D
TOV-112D PT-res
Ovaryspheres area (A.U. x10³)
p<0.0001
sh-Ctrl
sh-Snail
Snail
Vinculin
E
TOV-112D PT-res
distance (uM)
p<0.0001
sh-Ctrl
sh-Snail
F
TOV-112D PT-res
Snail mRNA expression
WT
KO
POLY-L
LM10
G
TOV-112D PT-res
PT-res WT
PT-res KO
MG132
hrs of adh.
ITGA6
Snail
GAPDH
H
TOV-112D PT-res
PT-res WT
PT-res KO
CHX
MG132
hrs of adh.
Snail
Snail (high expo)
Vinculin
I
TOV-112D PT-res
LiCl
MG132
hrs of adh.
pGSK-3β (S9)
GSK-3β
Snail
GAPDH
J
PT-res
WT
KO
NA
hrs of adh.
pLyn (Y397)
Lyn
pSRC
SRC
Snail
GAPDH
K
PT-res
Saracatinib
hrs of adh.
pSRC
SRC
Snail
GAPDH
L
LMs
β1
α6
Lyn
Snail
Invasion
Adhesion
Stemness

Figure EV3. ITGA6 engagement modulates Snail expression.

(**A**) Western blot analysis of the indicated proteins in whole lysates of TOV-112D PT-res cells plated on LM10 coated dishes in the presence of IgG (as control) or of GoH3 Ab. (**B**) Western blot analysis evaluating ITGA6 and Snail expression in whole lysates of TOV-112D PT-res ITGA6WT and ITGA6KO cells plated or not on LM10 coated dishes for 1 and 3 h. (**C**) Western blot analysis evaluating the expression of ITGA6 and Snail in TOV112D PT-res ITGA6WT and KO cells and ITGA6KO transfected with vectors expressing the two isoforms of ITGA6 (isoform A, A6A and isoform B, A6B). Whole lysates were analyzed at the indicated time points after LM10 adhesion. (**D**) Graph reporting the area of ovaryspheres formed by TOV-112D PT-res cells stably transduced with control shRNA (sh-ctrl) or Snail shRNAs and plated on a mesothelial cell monolayer. In the inset Western blot reports Snail expression in the used cells. Data represent the median (±SD) of three independent experiments performed in triplicate in which at least 150 randomly selected cells were analyzed. (**E**) Graph reporting the distance covered by the individual cells described in (**D**), in Matrigel evasion assay calculated starting from the edge of the drop (mean ± SD of three independent experiments in which at least 10 randomly selected fields were analyzed). In D and E, statistical significance was determined by a two-tailed, unpaired Student's t-test (Exact *p* values were reported on graphs). (**F**) qRT-PCR evaluating the mRNA expression of Snail in TOV-112D PT-res cells plated on Poly-lysine (negative control) or on LM10 coated dishes. mRNA expression (mean ± SD) was analyzed in triplicate and normalized to actin housekeeping gene expression. (**G**, **H**) Western blot analysis evaluating the expression of ITGA6 and Snail in TOV112D PT-res WT and KO cells plated on LM10 for 1 and 3 h and treated or not with the proteasome inhibitor, MG132 alone (**G**) or with CHX (**H**). (**I**) Western blot analysis evaluating the expression of ITGA6 and Snail in TOV112D PT-res ITGA6WT cells plated on LM10 for 1 and 3 h and treated or not with MG132 or with the GSK3β inhibitor, LiCl. (**J**) Western blot analysis evaluating the expression of the indicated proteins in lysates of TOV-112D PT-res ITGA6WT and ITGA6KO cells plated or not on LM10 coated dishes for 1 and 3 h (NA = not adherent). (**K**) Western blot analysis evaluating the expression of the indicated proteins in lysates of TOV-112D PT-res ITGA6WT cells plated on LM10 coated dishes for 1 and 3 h in the presence or not of the pan-SRC inhibitor Saracatinib. (**L**) Schematic representation of obtained results. After adhesion to LM10, ITGA6 activates Lyn/Src pathway and regulates Snail protein stability to mediate invasion, adhesion, and stemness of tumor cells. In all western blots of the figure, GAPDH, Tubulin, or Vinculin were used as loading control, as indicated.

 © The Author(s)

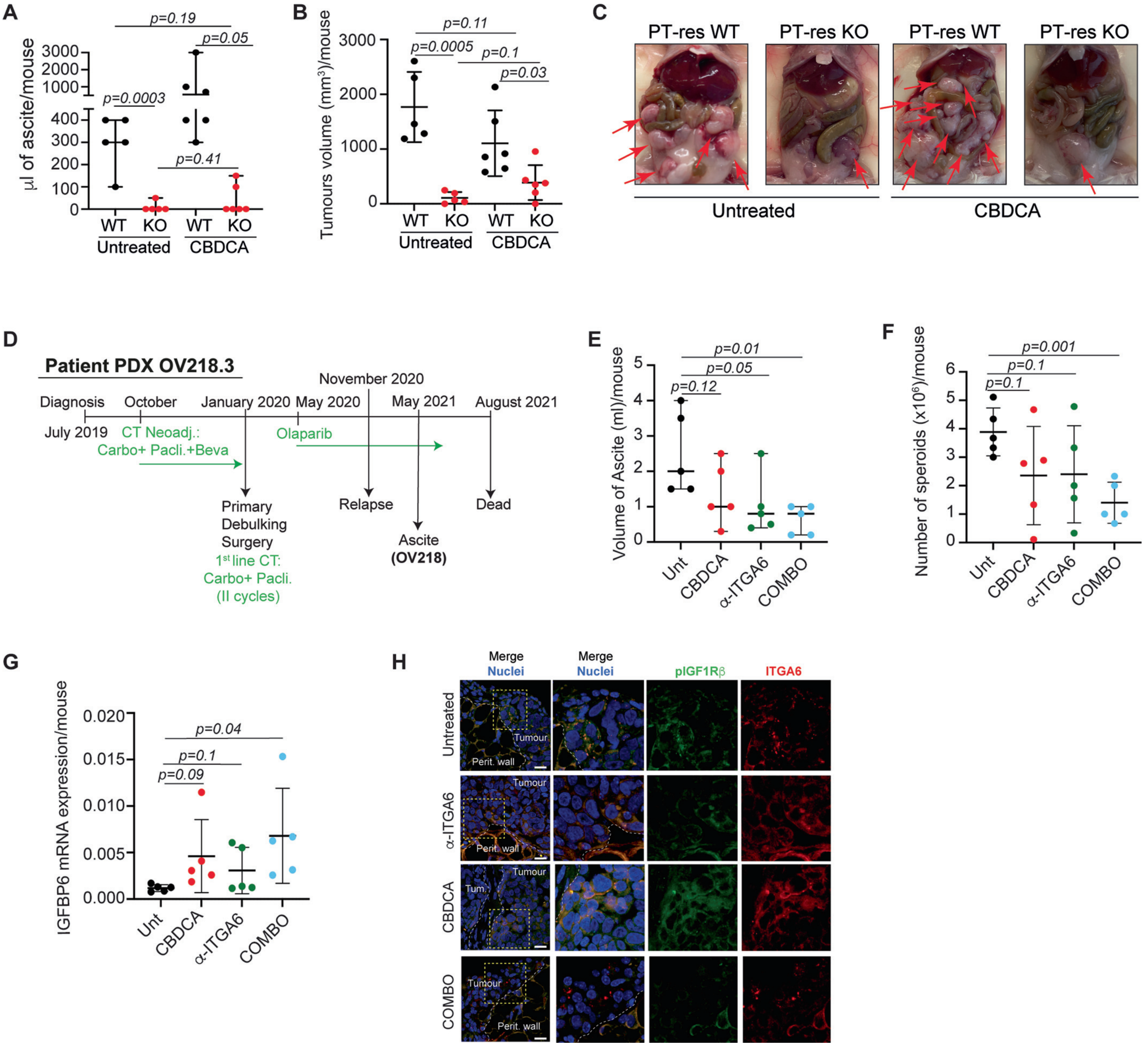

Figure EV4. ITGA6 is a druggable target necessary for in vivo cell spreading and response to PT.

(**A**, **B**) Graph reporting the volume of ascitic fluids (**A**) and of explanted macroscopically identified tumors of NSG mice injected intraperitoneally with PT-res ITGA6 WT ($n = 11$) and KO cells ($n = 11$) and treated ($n = 6$) or not ($n = 5$) with CBDCA 30 mg/kg 3 times per week for 2 weeks. (**C**) Typical images of mice described in (**A**) and (**B**). Red arrows indicated the presence of macroscopically visible tumors. (**D**) Clinical history reporting the timeline of surgery, chemotherapy treatments (in green) and ascites collection of EOC patient who donate her ascites to establish PDX OV218.3 (see Methods section). (**E**, **F**) Graph reporting the volume of ascitic fluids (**E**) and number of tumor spheroids (**F**) in NSG mice ($n = 5$/group of treatment) injected with PDX OV218.3 and treated or not with the specific anti-ITGA6 blocking antibody P5G10, with CBDCA or with the combination of both according to the scheme reported in Fig. 6H. (**G**) IGFBP6 mRNA expression in tumor cells in ascites of mice described in (**E**, **F**). mRNA expression was analyzed in triplicate and normalized to actin housekeeping gene expression. The mean (±SD) expression for each mouse is reported in the graph. (**H**) IF analyses evaluating the expression of pIGF1Rβ (green) and ITGA6 (red) on peritoneal metastases collected from mice treated as indicated (nuclei are in blue). White dashed lines indicate the boundary between tumor masses and the peritoneal wall. Yellow dashed boxes represent the areas magnified in the zoomed images, on the right. Scale bars = 20 µm. In the figure, statistical significance was determined by a two-tailed, unpaired Student's t-test (Exact *p* values were reported on graphs). Bars represent Standard Deviation.
